# High-throughput kinome-RNAi screen identifies protein kinase R activator (PACT) as a novel genetic modifier of CUG foci integrity in myotonic dystrophy type 1 (DM1)

**DOI:** 10.1371/journal.pone.0256276

**Published:** 2021-09-14

**Authors:** Nafisa Neault, Sean O’Reilly, Aiman Tariq Baig, Julio Plaza-Diaz, Mehrdad Azimi, Faraz Farooq, Stephen D. Baird, Alex MacKenzie

**Affiliations:** 1 Department of Cellular and Molecular Medicine, Faculty of Medicine, University of Ottawa, Ottawa, ON, Canada; 2 Children’s Hospital of Eastern Ontario Research Institute, Ottawa, ON, Canada; University of Texas MD Anderson Cancer Center, UNITED STATES

## Abstract

Myotonic Dystrophy Type 1 (DM1) is the most common form of adult muscular dystrophy (~1:8000). In DM1, expansion of CTG trinucleotide repeats in the 3’ untranslated region of the dystrophia myotonica protein kinase (DMPK) gene results in DMPK mRNA hairpin structures which aggregate as insoluble ribonuclear foci and sequester several RNA-binding proteins. The resulting sequestration and misregulation of important splicing factors, such as muscleblind-like 1 (MBNL1), causes the aberrant expression of fetal transcripts for several genes that contribute to the disease phenotype. Previous work has shown that antisense oligonucleotide-mediated disaggregation of the intranuclear foci has the potential to reverse downstream anomalies. To explore whether the nuclear foci are, to some extent, controlled by cell signalling pathways, we have performed a screen using a small interfering RNA (siRNA) library targeting 518 protein kinases to look at kinomic modulation of foci integrity. RNA foci were visualized by *in situ* hybridization of a fluorescent-tagged (CAG)_10_ probe directed towards the expanded DMPK mRNA and the cross-sectional area and number of foci per nuclei were recorded. From our screen, we have identified PACT (protein kinase R (PKR) activator) as a novel modulator of foci integrity and have shown that PACT knockdown can both increase MBNL1 protein levels; however, these changes are not suffcient for significant correction of downstream spliceopathies.

## Introduction

Myotonic Dystrophy Type 1 (DM1) is the most common form of adult muscular dystrophy with a prevalence of 1:8000 worldwide; in some regions of Quebec, Canada, the prevalence is as high as 1:500. DM1 is caused by a CTG repeat expansion in the 3’ untranslated region (UTR) of the dystrophia myotonica protein kinase (DMPK) gene [1]. Unaffected individuals have a range of 5–37 repeats; mild symptoms begin to manifest in adulthood for individuals with expansions of approximately 50 CTG repeats [2]. The length of the CTG repeats is directly proportional to the severity of the disease and inversely proportional to the age of onset [3, 4]. The ubiquitous expression of *DMPK* results in DM1 being a multisystemic disorder [5]; disease manifestations include myotonia, insulin resistance, cardiac defects (arrhythmias), testicular atrophy, cataracts, cognitive dysfunction, and muscle wasting, among others [2].

DM1 pathogenesis arises from an RNA gain-of-function mechanism. Expanded CTG repeats of the mutant DMPK mRNA form hairpin secondary structures through C-G base pair binding, resulting in distinct intra-nuclear foci [6, 7]. In addition to CUG hairpins, the nuclear foci contain RNA-binding proteins such as the transcription factor Muscleblind-like 1 (MBNL1) [8, 9]. MBNL1 sequestration in nuclear foci depletes the biologically active form in the nucleoplasm and cytoplasm, resulting in dysregulated splicing for several target genes. This leads to diverse DM1 features–for example, chloride channel (CLC1) spliceopathy accounts for myotonia, insulin receptor (IR) mis-splicing leads to insulin resistance, cardiac troponin T (cTNT) spliceopathy is linked to cardiac myopathies, and aberrant alternative splicing of sarcoplasmic/endoplasmic reticulum calcium ATPase (SERCA1) potentially plays a role muscle wasting [10–15]. It has been shown that overexpression of MBNL1 protein corrects DM1-associated aberrant splicing and certain physical signs in DM1 animal models, such as myotonia [16, 17]. We hypothesize that shrinking the number and/or size of the foci will restore nucleoplasmic MBNL1 levels and thus have beneficial effects on splicing dysregulation, such as has been documented following DMPK mRNA-directed antisense oligonucleotide (ASO)-mediated foci shrinkage [18].

Advances in sequencing and genomics have enabled the compilation of whole kinome/genome RNA interference (RNAi) libraries for many model organisms; high-throughput RNAi screens mimicking single gene knockdowns using platforms similar to those used for large scale chemical screening have become commonplace [19]. We present a kinome RNAi screen performed on DM1 patient fibroblasts using DM1 RNA foci (size and number) as a measure of disease pathophysiology. Through this screen, we have identified the kinase activator PACT (protein kinase R (PKR) activator) as a modulator of foci integrity in DM1 patient fibroblasts.

## Materials and methods

### Cell culture

All DM1 patient human fibroblasts (HF) and Control HF 4 were obtained from Coriell Biorepository (Control HF4, GM04603; DM1_66CTG_, GM06076; DM1_500CTG_, GM03987; DM1_1000CTG_, GM04033; DM1_1600CTG_, GM04602; DM1_2000CTG_ 1, GM03132; DM1_2000CTG_ 2, GM03989; DM1_2000CTG_ 3, GM03759) and the remaining Control HF cell lines were obtained from the CHEO Care for Rare repository (Control HF 1, CH3014F; Control HF 2, CH3009; Control HF 3, CH3013). All cells were maintained at 37°C and 5% CO_2_ in growth media (High Glucose Dulbecco’s Modified Eagle Media (DMEM), Gibco), supplemented with 0.292mg/mL L-glutamine in 0.0085% NaCl (HyClone), 10% fetal calf serum (FCS; HyClone) and 100units/mL penicillin/ 100μg/mL streptomycin (HyClone).

Cells were passaged by trypsinization when they reached 70–90% confluence and were split at a ratio of one-third to one-fourth to achieve 20–30% seeding density; for experimental set-up, cells were manually counted using a Bright-Line hemocytometer (Sigma) under an EVOS AMG bright-field microscope at 10x magnification (Thermo Scientific). For passaging and plating cells, old media was aspirated, and cells were washed with 1X PBS (Fisher Scientific) to remove residual serum from the FCS; the PBS (Fisher Scientific) was then aspirated, cells were incubated in 1X (0.25%) trypsin (Gibco; reconstituted in 15mM NaCl (Fisher Scientific), 0.5mM EDTA (Sigma), and 1X PBS (Fisher Scientific)) for 3-7min at 37°C to lift them from the plate, and then trypsin was inhibited using complete growth media containing fetal calf serum (FCS).

### Transfection of siRNA

A kinome subset of the Human Druggable Genome siRNA Set Version 2.0 (Qiagen) [20, 21] comprised of pools of four unique siRNA sequences targeting each specific kinase gene, was used. Fibroblasts were reverse transfected with siRNA using RNAi MAX (Invitrogen). The siRNAs were generally used at a final concentration of 5-20nM, relative to the final transfection volume (diluted siRNA, lipofectamine, cell suspension in media); and the lipofectamine was used at a ratio of 2μL per 1mL final transfection volume. The siRNA and lipofectamine were first separately diluted in optiMEM reduced serum medium (Gibco) and incubated at room temperature for 5min–each siRNA/optiMEM and lipofectamine/optiMEM dilution represented one-eighth of the final transfection volume. The diluted siRNA and lipid were mixed together and incubated at room temperature for at least 20min to allow the siRNA-lipid complex to form–this solution of siRNA-lipid complex represented one-fourth of the final transfection volume. The siRNA-lipid complex was mixed with trypsinized cells which were resuspended in complete growth media and plated in a tissue culture dish. The treatments were performed at 37°C in the presence of 5% CO_2_. Each sample set contained a negative control non-targeting siRNA (Qiagen). Transfection efficiency was assessed by reverse transfecting 20nM of the cytotoxic AllStars Death Control siRNA (Invitrogen); the degree of cell death corresponds to transfection efficiency.

### Fluorescence *in situ* hybridization (FISH)

Cells were grown and treated in 384-well falcon plates (VWR, all wells were used) were fixed by adding a 1:1 volume of media-to-formaldehyde to a final concentration of 4% formaldehyde for 10min at room temperature, following which the wells were washed 5x3min with 1X PBS. The cells were then permeabilized overnight in 70% ethanol at 4°C. Ethanol was removed and the cells were further incubated 5x3min with 1X PBS. The Alexa555-(CAG)_10_ probe (Invitrogen) was diluted to 0.3ng/μL in DIG Easy Hyb Granules Buffer (Roche) and hybridized for at least 2hrs at 37°C. The plates were washed again 5x3min with 1X PBS and subsequently incubated with 5 μg/mL Hoechst 33342 DNA stain (Sigma) diluted in 1X PBS (Fisher Scientific) at room temperature for 10-20min to stain the nuclei. The wells were washed 5x3min with 1X PBS and 50μL of 1X PBS was added to the wells for imaging. The plates were covered with adhesive, optically clear Excel Scientific Inc SealPlate (Fisher Scientific).

The Alexa555-(CAG)_10_ probe (custom oligos from Invitrogen) was resuspended from a lyophilized pellet in nuclease-free Tris-EDTA buffer, pH 8.0 (Ambion). The resuspended probe was further aliquoted into light-protective, dark tubes and stored long-term at -80°C. Hoechst 33342 (Sigma) was aliquoted into light-protective dark tubes and stored at 4°C.

### CUG foci image analysis

After FISH and Hoechst staining, the plates were scanned using the automated Opera high-content screening system (Perkin Elmer), and 16 fields were captured per well to acquire a representative sample of images (Hoechst: excitation at 346nm, emission at 460nm) and CUG foci (Alexa555-(CAG)_10_: excitation at 555nm, emission at 565nm). Images were uploaded to Columbus software (Perkin Elmer) where the RNA-foci were quantified in each cell. Cells were scored on the number of foci detected per nucleus and on the total surface area of foci as determined by the number of pixels in each focus. A minimum surface area threshold was included as the lower end of detection to ensure that the background signal was not included in quantification. The Z-score (the number of standard deviations from the mean) was then used to rank samples based on the number of standard deviations each data point was from the sample mean. Negative Z-score values represent a decrease in the parameter (foci size or number) and a positive value indicates an increase. Samples that had a mean z-score less than -2.0 in either foci number per nuclei or mean foci area from three repeats were considered hits for further validation.

### Western blot analysis and antibodies

In general, cells were washed with 1X PBS and lysed on the plate for 20 min at 4°C using RIPA lysis buffer containing aprotinin, leupeptin, and PMSF each at 10mg/mL. The lysed samples were collected by scraping, further subjected to sonication (3x15sec at 20% amplitude) and centrifuged at 13,000g for 30min at 4°C; the supernatant was then recovered. Protein concentrations were determined by Bradford protein assay using a Bio-Rad protein assay kit (Richmond, CA, USA). Loading samples were prepared in Laemmli buffer containing SDS and boiled for 5min at 95°C. The samples were separated by 11% SDS-PAGE gel and transferred to 0.45μm nitrocellulose membrane. The membranes were blocked in 5% milk in 1X PBS or TBS containing 0.1% Tween-20.

Primary antibodies were purchased from Abcam and used at the following concentrations: MBNL1-mouse IgG (1:5000), Tubulin-mouse IgG (1:20000), PACT-rabbit IgG (1:1000–2000), PKR-rabbit IgG (1:1000), PKR (phospho T446)-rabbit IgG (1:1000). HSP90 primary antibody was purchased from Cell Signalling and used at a dilution of 1:2000. For PACT-rabbit IgG, PKR-rabbit IgG, and PKR (phospho T446)-rabbit IgG, HRP-linked secondary antibodies were diluted in 3% BSA and for all other primary antibodies, HRP-linked secondary antibodies were diluted in 5% milk and used for 1hr at room temperature: anti-mouse (1:5000) and anti-rabbit (1:2000) (Cell Signalling). Antibody complexes were visualized by autoradiography using ECL western blotting system (GE Healthcare and BioRad) and X-Ray film (Kodak). Quantification was performed by scanning the autoradiographs and densitometric signal intensities determined using Image J software.

### MBNL1 immunohistochemistry

Media was removed from the cells and a MeOH/Acetone (1:1) solution (-20C) was added for 5 mins. Cells were washed with 1X PBS and blocked with a 5% normal goat serum, 0.3% Triton X-100 in 1X PBS (room temp, 60 min). The cells were washed again with 1X PBS, then primary MBNL1 (Developmental Studies Hybridoma Bank, 4A8) antibody diluted 1:4 in 1% bovine serum albumin (BSA)/0.3% Triton X-100 in 1X PBS was added to the cells overnight at 4C. CY3 conjugated goat anti-mouse secondary antibody (Jackson Immuno, 115-165-003) diluted to 1:5000 in 1% bovine serum albumin (BSA)/0.3% Triton X-100 in 1X PBS and added to the cells for 2 hours at room temperature in the dark after washing with 1X PBS. Nuclear counterstain, Hoechst 33342, was added to the cells at a dilution of 1:1000 in 1X PBS after subsequent washes with 1X PBS.

### RT-qPCR analysis

Total RNA was isolated using RNEasy Micro Kit (Qiagen) according to the manufacturer’s protocol. RNA was quantified using Nano Drop and reverse transcribed to cDNA using iScript Reverse Transcription Kit (BioRad) according to manufacturer’s protocol. RT-qPCR was used for the quantification of all mRNA transcripts using iQSybrGreen Master Mix (BioRad). All primers were optimized for use at 60°C annealing temperature and the SybrGreen signal was acquired after extension at 72°C.

The primer sequences are (5’ → 3’):

DMPK FWD GGCTCACTGCCATGGTGA; REV GCTGTTTCATCCTGTGGGGA

PACT FWD GCCATGCACATCAGAGAAAG; REV AGGGCCTGTTAGTGCTGTCC

MBNL1 FWD TGATTGTCGGTTTGCTCATC; REV TTGATCTTGGCTTGCAAATG

GAPDH FWD TGCACCACCAACTGCTTAGC; REV GGCATGGACTGTGGTCATGAG

HPRT FWD TGACACTGGCAAAACAATGCA; REV GGTCCTTTTCACCAGCAAGCT

SERCA1-A FWD CCCTCCTCCATCTCTGAGC; REV AGCTCTGCCTGAAGATGTGTC

SERCA1-AB FWD CTCCATCTGCCTCTCCATGT; REV CTTGAGGACCATGAGCCACT

IR-B FWD AAAACCTCTTCAGGCACTGG; REV CGACTCCTTGTTCACCACCT

IR-AB FWD GGCAACATCACCCACTACCT; REV ACTCGAATGGTGGAGACCAG.

### PKR inhibition

PKR inhibitor C16 (Sigma and Merck) was dissolved in DMSO (Sigma). Cells were grown in 384-well falcon plates (VWR). The cells were treated with DMSO or C16 PKRi every 48hrs for up to 7days. The treatments were performed at 37°C in the presence of 5% CO_2_. Following treatment, cells were fixed, stained by RNA FISH, and analysed for foci content as previously described.

## Results

### Nuclear RNA foci in DM1 patient fibroblasts are representative of pathogenic CTG repeat expansions

The robust quantification of nuclear RNA foci in DM1 cell lines was initially established as a disease-specific marker. FISH with Alexa555-(CAG)_10_ fluorescent probe identified nuclear CUG foci in DM1 primary skin fibroblast cells (DM1 HF), but not in unaffected control human fibroblast cells (Control HF), consistent with a high signal to noise ratio (S1 Fig). Six DM1 cell lines containing 500 CTG repeats or more were found to have on average two foci per nucleus, regardless of CTG repeat size. In the DM1 cell line containing 66 CTG repeats (DM1_66CTG_ HF), the foci number was comparable to that observed in Control HFs, suggesting a threshold repeat-size for either nuclear foci formation or detection. DM1 HF containing 500 CTG repeats (DM1_500 CTG_ HF) were selected for the kinome-wide siRNA screen given their efficient growth and transfection capacity.

### Kinome RNAi screen identifies PACT as a moderator of foci size and number in DM1 HF

**Fig 1:**
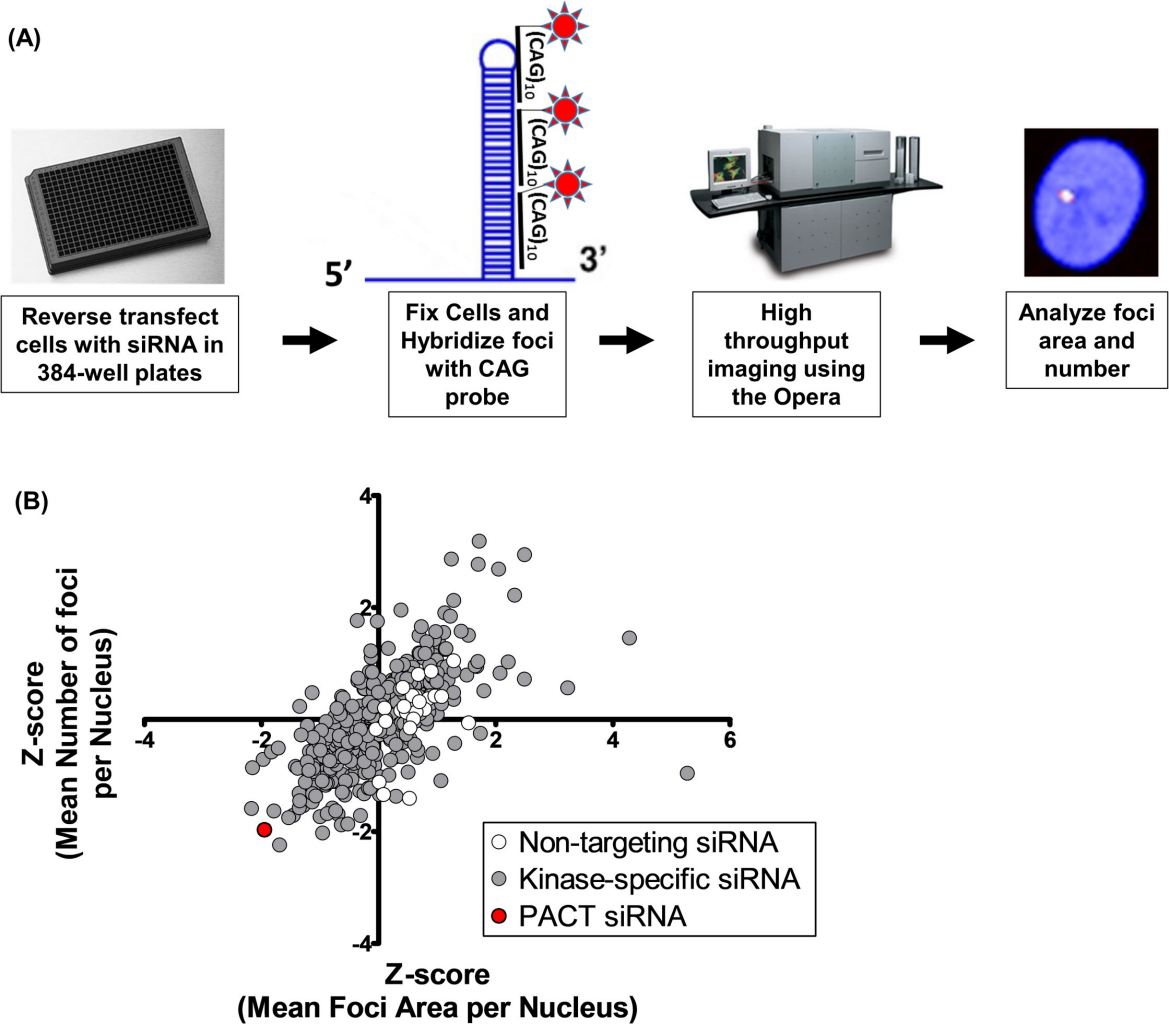
Kinome wide RNAi screen reveals several potential modulators of DM1 foci. The screen was done in triplicate using 10nM of pooled siRNA with an end-point readout conducted at 96 hrs. The data were normalized to the sample mean per plate and is represented as average Z-score values.

Using the methodology depicted in Fig 1A, the transfection of kinase-specific RNAi revealed the down regulation of PACT (protein kinase R activator or PRKRA (protein kinase, interferon inducible double stranded RNA dependent activator)) in particular conferred foci reduction (Fig 1B). The greatest impact on nuclear foci integrity of all kinases was observed with PACT knockdown, which resulted in the reduction of size and number of nuclear RNA foci by approximately two standard deviations (Z-score: foci count -1.97, foci area -1.95; S1 Table, PRKRA) after 96hrs. PACT is a kinase activator involved in the subcellular stress response and interacts with double-stranded RNA [22] as well as acting as a pro-apoptotic factor that activates PKR [23]. PKR (protein kinase R activator) has been found to be over-expressed in DM1 cells and has been suggested to bind the double stranded DMPK CUG RNA [24, 25]. Given this connection to DM1 dysregulation and PACT siRNA having had the greatest impact on foci integrity in the primary screen, PACT was selected for further evaluation.

**Fig 2:**
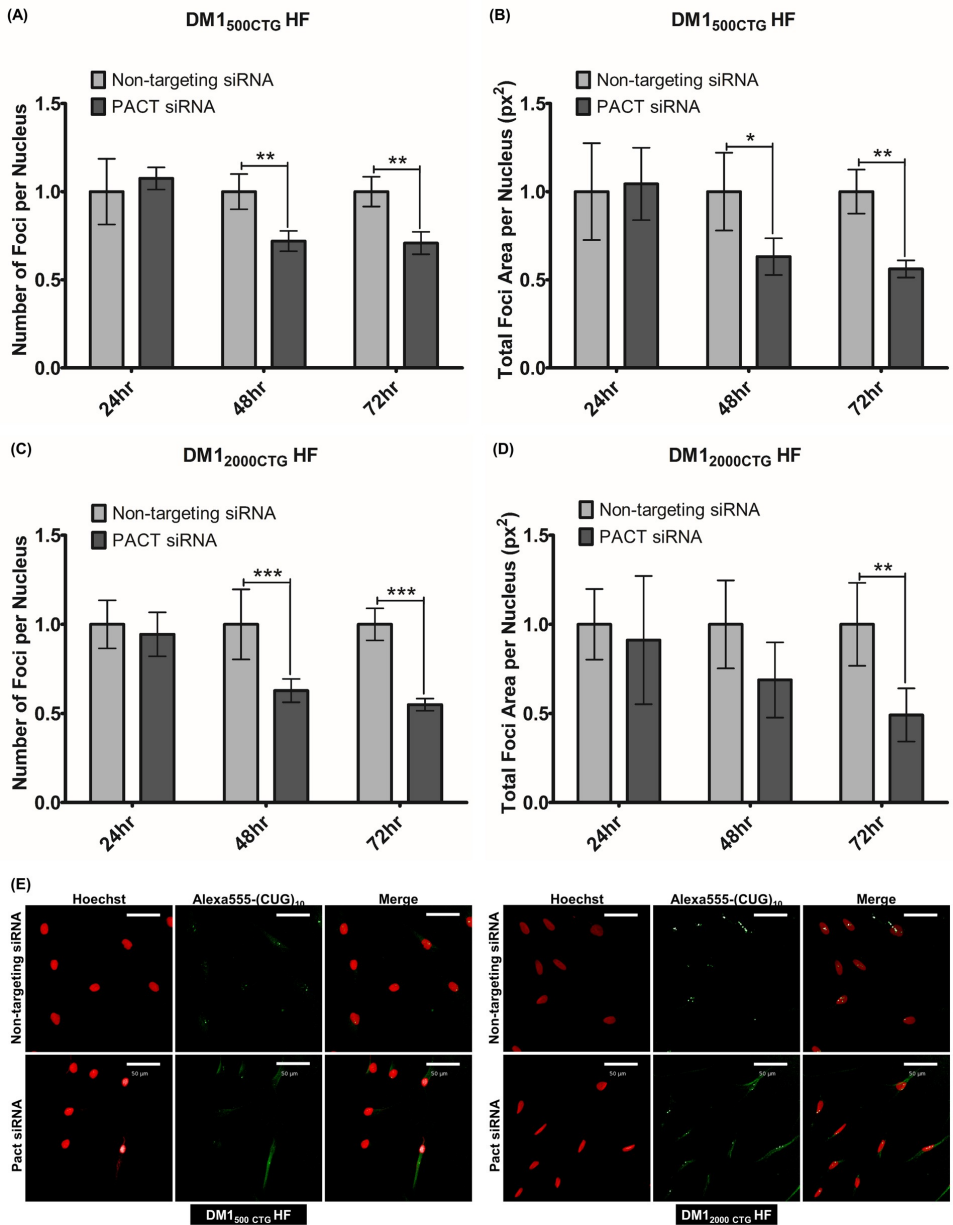
PACT knockdown reduces foci in DM1 patient fibroblasts. (A-B and C-D) Effect of PACT knockdown on foci in DM1_500 CTG_ and DM1_2000 CTG_, respectively. Cells were probed with Alexa-555 (CAG) _10_ probes to detect and image foci. Quantification of (A and C) number of foci per nucleus and (B and D) total foci area per nucleus was done using the Columbus software (n = 4; two-way ANOVA; error bars represent SD, details of the foci signal quantification using the Columbus software are shown in S2 Table). For statistical analysis, each trial consisted of mean data from 3–9 replicate wells per sample with the corresponding SD. The data were converted to fold change relative to non-targeting siRNA to accommodate plate-to-plate variability and the SD was adjusted to the same ratio. The fold change and SD data were averaged across all trials. (E) Representative images of nuclear foci (red–Hoechst; green–CUG foci) after RNA FISH of CTG foci in patient skin fibroblasts treated with Non-targeting or PACT siRNA for 72hrs. White scale bars represent 50um. *P < 0.05, **P < 0.01, ***P< 0.001.

The kinase siRNA library (Qiagen) used in this study is comprised of pools of four unique siRNA sequences targeting specific kinase genes. To confirm that PACT was an on-target hit, the impact of the four PACT siRNAs, individually and pooled, on PACT protein level and nuclear foci number and size were assessed in DM1_500 CTG_ patient cells. All siRNAs knocked down the PACT protein to varying degrees (S2A and S2B Fig). Similarly, all four siRNAs reduced foci to some extent, ranging from 10–45% reduction, depending on time-point and siRNA (S2C and S2D Fig). Importantly, siRNA1 and 3, the two siRNAs conferring the greatest foci area reduction also generated the greatest PACT protein decrease (S2B and S2D Fig). Given these findings, we concluded that the foci modulation is due to PACT knockdown and not an off-target effect of the siRNA. The PACT siRNA having the most robust effect on PACT protein knockdown and foci reduction (PACT siRNA 1, referred to as PACT siRNA from this point onwards) was used for further analysis. A time-course of PACT siRNA treatment revealed a reduction in PACT protein level starting at 24hrs for DM1_500 CTG_ HF and 48hrs for DM1_2000 CTG_ HF (S3 Fig), while the reduction of both foci number and area were completed by 48hrs (Fig 2).

Given CUG-foci are comprised of mutant DMPK mRNA, an obvious question was whether the observed reduction in foci was a direct result of the reduction in DMPK mRNA itself, either by transcriptional downregulation and/or by decreasing DMPK mRNA stability. Quantification by qPCR analysis revealed although PACT knockdown significantly reduced CUG-foci, there was no reduction in DMPK mRNA in either cell line (S4A Fig–DM1_500 CTG_ HF; S4D Fig–DM1_200 CTG_ HF).

### PACT knockdown increases MBNL1 expression in DM1 cells but only imparts a partial, non-statistically significant normalization of SERCA1 spliceopathy

**Fig 3:**
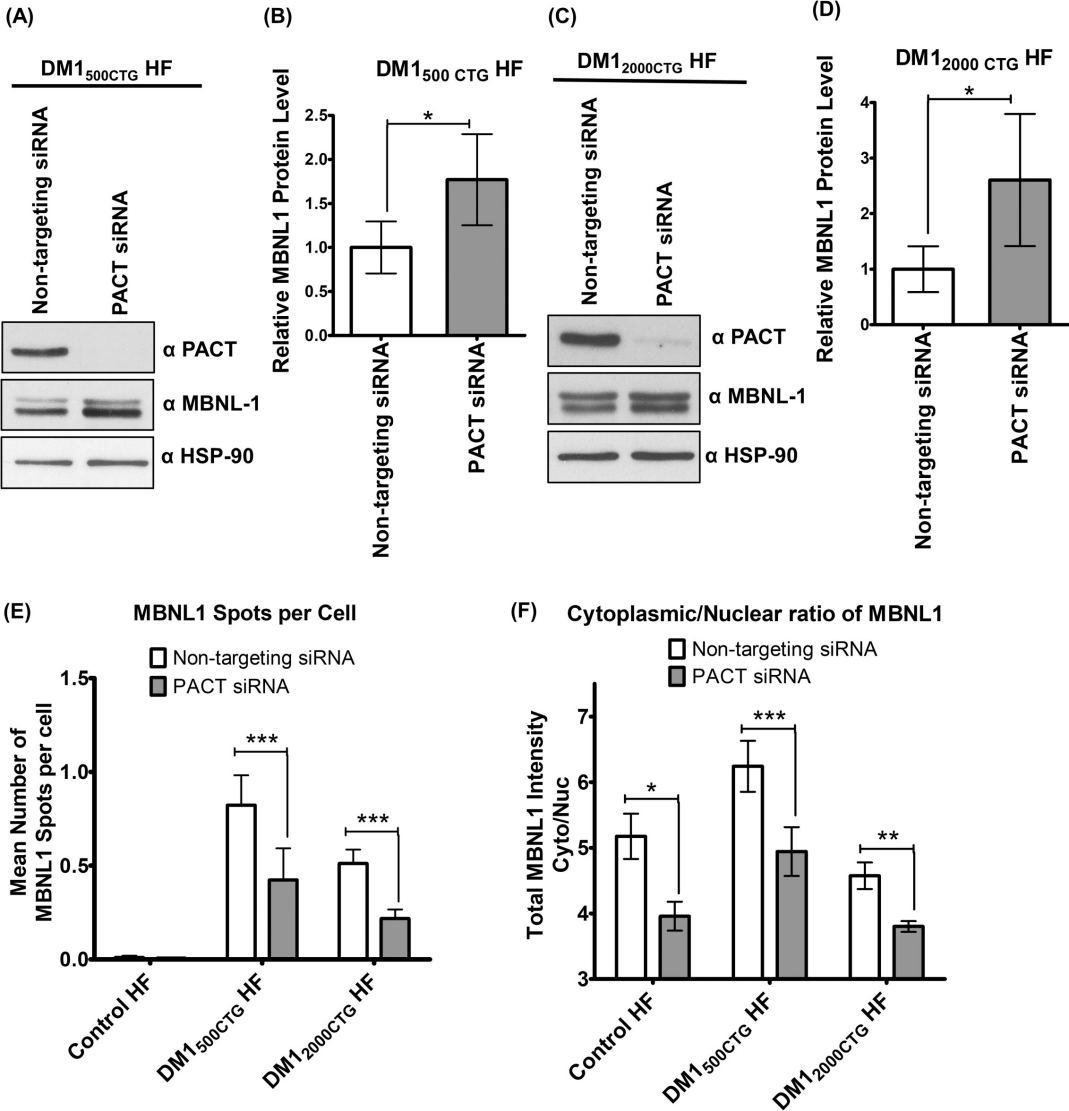
PACT knockdown induces MBNL1 protein expression. Western blot analysis of MBNL1 protein levels upon PACT knockdown in fibroblasts from (A) DM1_500 CTG_ HF and (C) DM1_2000 CTG_ HF. Corresponding quantification of MBNL1 protein levels in (B) DM1_500 CTG_ HF and (D). Quantification of MBNL1 positive foci in HF DM1_500 CTG_ and DM1_2000 CTG_ treated with PACT and scrambled siRNA. (E). The cytoplasmic to nuclear MBNL1 ratio was also quantified (F; details of the MBNL1 signal quantification using the Columbus software are shown in S3 Table). MBNL1 protein level was normalized to HSP90 loading control, averaged across trials and presented as relative fold-change to Non-targeting siRNA (n = 4; unpaired t-test; error bars represent SD). *P < 0.05, **P < 0.01, ***P< 0.001.

As outlined above, the major pre-mRNA splicing regulator MBNL1 undergoes pathogenic sequestration by the expanded DMPK mRNA in DM1. One potential DM1 therapeutic avenue is therefore to either free MBNL1 from foci or increase MBNL1 levels by some other mechanism. The impact of PACT knockdown on MBNL1 protein levels in DM1 cells was thus assessed by western blot analysis. PACT knockdown induces an approximate two to three-fold induction of MBNL1 protein in both DM1_500 CTG_ and DM1_2000 CTG_ patient fibroblasts (Fig 3). While PACT mRNA was reduced in both cell lines (S4B and S4E Fig), MBNL1 transcript levels were not significantly changed by PACT siRNA treatment, suggesting a post transcriptional mechanism for MBNL1 protein induction (S4C and S4F Fig).

To more fully explore the status of MBNL1 with PACT knock down, immunolocalization of MBNL1 protein in cells treated with PACT and non-targeting siRNA was conducted. MBNL1 positive foci were uniquely detected in DM1 cells; their number was halved by treatment with PACT siRNA (Fig 3E, p<0.001). The cytoplasmic to nuclear MBNL1 ratio was also determined; a statistically significant increase in the nuclear fraction was observed with PACT RNAi treatment in all cell lines tested with the greatest effect seen in DM1 cells (Fig 3F).

To investigate whether the increase in MBNL1 protein level and/or reduction in foci number/size upon PACT knockdown resulted in the normalization of dysregulated splicing in DM1, transcript ratios for genes encoding SERCA1 and IR were quantified using a qPCR assay. SERCA1 is responsible for the regulation of intracellular Ca^2+^ homeostasis in skeletal muscle cells; its mis-splicing is thought to contribute to the disabling, progressive muscle wasting observed in DM1 [13, 15]. Normally, there exists a single, full length isoform (SERCA1-A). Uniquely in DM1 patients, an isoform missing exon 22 (SERCA1-B) is observed with a corresponding reduction in the level of full-length SERCA1-A. In the case of the IR gene, there is an IR-B isoform, which encodes an insulin-sensitive receptor, and IR-A isoform, which lacks exon 11 and encodes a less-sensitive insulin receptor. In unaffected individuals, the IR exists predominantly as IR-B whereas in DM1 patients, the relative level of IR-B is reduced [10, 12]. In order to assess SERCA1 and IR splicing, we designed a transcript-specific RT-qPCR assay for each gene; for this assay, SERCA1-A (using primer sets with one primer within exon 22) was measured relative to total SERCA1 (using primers amplifying exons common to both SERCA1-A and SERCA1-B) as the reference gene and IR-B (using primer sets with one primer within exon 11) was measured relative to total IR (using primers amplifying exons common to both IR-A and IR-B) as the reference gene. RT-qPCR quantification revealed a decreased level of SERCA1-A in DM1 HF cells compared with control HF cells (S5A Fig). The significant variability observed in control HF cell IR-B transcript levels (S5B Fig) precluded a rigorous assessment of PACT knockdown on IR spliceopathy.

**Fig 4:**
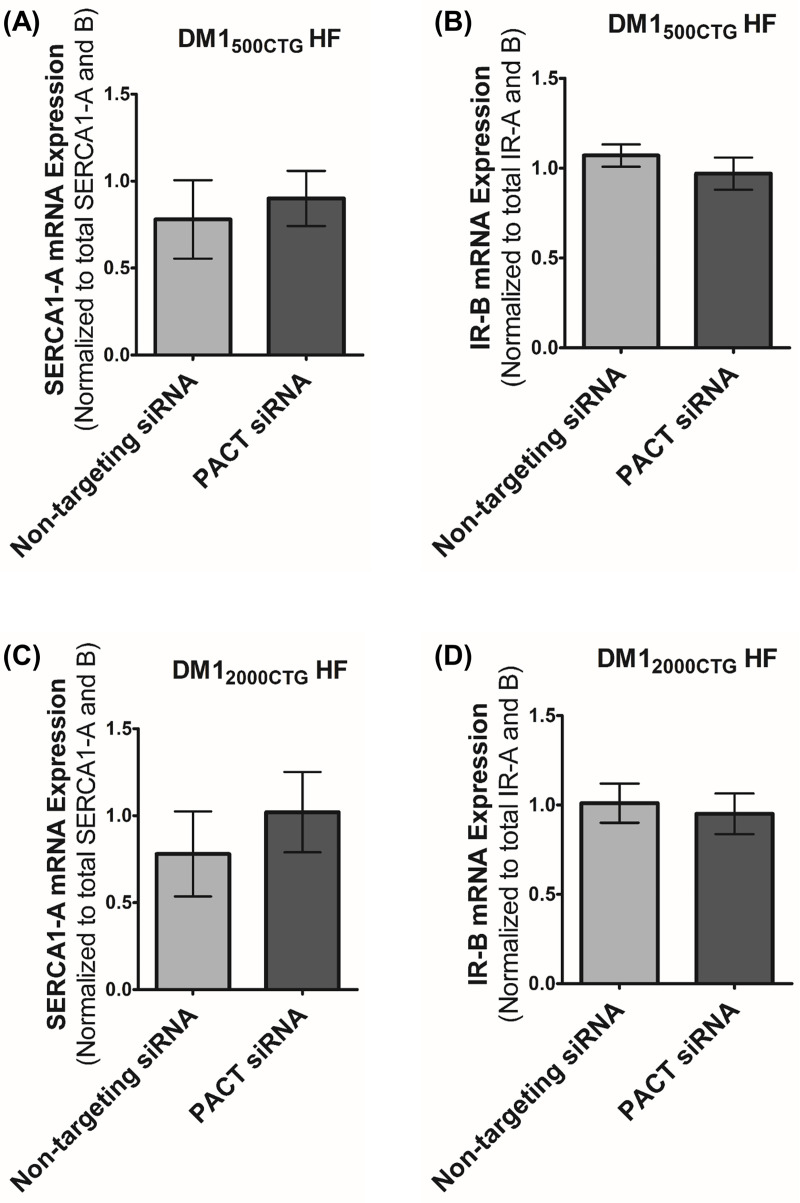
PACT knockdown does not impact IR splicing and imparts a partial, non-statistically significant trend towards SERCA1 splicing correction in DM1 cells. RT-qPCR analysis of (A and C) SERCA1 and (B and D) IR alternative splicing upon PACT knockdown in DM1_500 CTG_ and DM1_2000 CTG_, respectively. The non-targeting siRNA treated sample in one trial was set as the “control” sample and all other samples–meaning all non-targeting siRNA and PACT siRNA treated sample from all other trials–were quantified relative to this “control” (n = 5; unpaired t-test; error bars represent SD).

As a positive control to ensure that these qPCR assays are capable of detecting changes in transcript ratios, a subset of control and DM1 HF cells were treated with non-targeting or MBNL1 siRNA. MBNL1 siRNA effectively reduced MBNL1 mRNA (S5C Fig) and reduced the relative levels of both SERCA1-A and IR-B (S5D and S5E Fig, respectively). Treatment with PACT siRNA resulted in a modest albeit non-statistically significant correction of SERCA1 splicing in the cell line with the 2000 CTG repeats (Fig 4A-DM1_500CTG_ HF, 4C-DM1_2000CTG_HF) with no observable impact discerned for the IR in either cell line (Fig 4B-DM1_500CTG_ HF, 4D-DM1_2000CTG_ HF).

### PACT knockdown increases MBNL1 protein in non-DM1 fibroblasts

The effects of PACT knockdown on MBNL1 levels, and SERCA1 and IR splicing were also assessed in Control HF cells (Control HF 2 in the full panel of cells; S6 Fig). PACT knockdown was confirmed at both the protein (S6A Fig) and mRNA (S6C Fig) level. A moderate but statistically significant increase in MBNL1 protein was observed (S6A and S6B Fig); the smaller increase seen in DMPK mRNA did not reach statistical significance (S6D Fig). Therefore, it appears that some of the PACT-mediated increase in MBNL1 is independent of the expanded CTG repeat that is characteristic of DM1 genotype. However, similar to what was observed in the DM1 HF, there was no change in DMPK mRNA (S6E Fig) or relative SERCA1-A and IR-B transcript levels (S6F and S6G Fig, respectively).

### Pharmacologic inhibition of PKR, a substrate of PACT, reduces nuclear foci only at toxic levels

**Fig 5:**
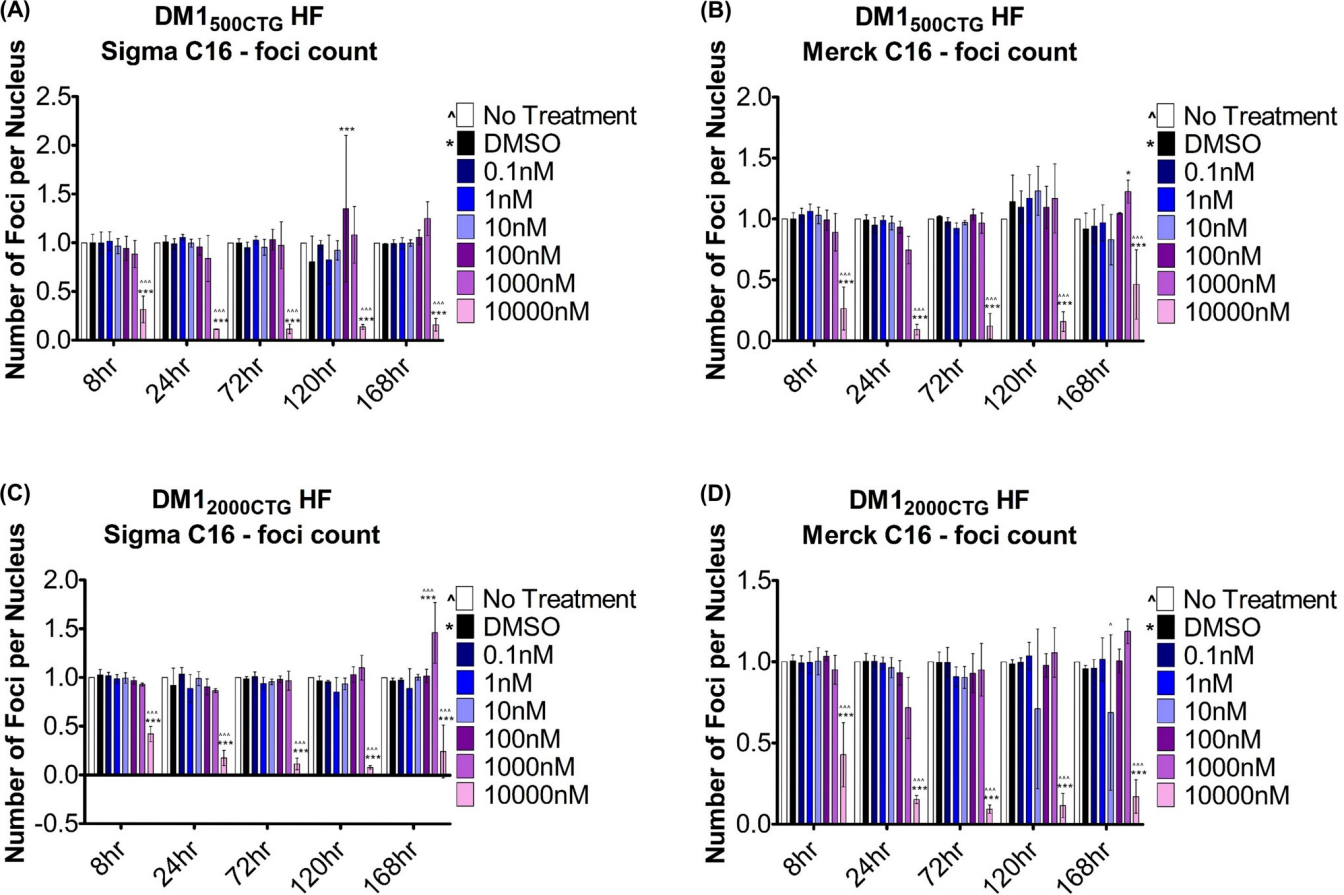
PKR inhibition does not significantly reduce foci integrity. Small molecule PKR inhibitor C16 was used to treat (A) DM1-500_500CTG_ HF or (B) DM1_2000cTG_ HF at doses ranging from 0.1nM-10,000nM for 8hrs up to 168hrs (7 days) following which (CUG)_n_ foci were detected by RNA FISH and foci number and area quantified using Columbus software (n = 3; two-way ANOVA; error bars represent SD). *P < 0.05, **P < 0.01, ***P< 0.001.

Given that PACT interacts with and activates PKR [26], the impact of PACT knockdown on PKR was explored. A modest reduction of PKR in DM1 cell lines alone, with no discernible impact on phosphorylated PKR levels in any cell line was observed following PACT RNAi treatment (S7 Fig). We next studied the impact of PKR inhibiting imidazole oxindole C16 (C16 PKRi), a compound previously reported to reduce DM1 foci [27], in our system. DM1_500CTG_ HF and DM1_2000CTG_ HF were treated with C16 PKRi; because of discordance with published results, C16 from two sources (Sigma and Merck) were used. Cells were treated C16 PKRi ranging in concentration from 0.1nM to 10μM for 8hrs, 24hrs, 72hrs, 120hrs and 168hrs. Foci number was only reduced with the highest C16 PKRi concentration (10μM), a level ten-fold greater than the previously reported foci reducing concentration of 1μM^27^ (and ~50X greater than PKR IC50 of 0.21 uM [28]), starting as early as 8 hrs (Fig 5). However, at this level there was also substantial cytotoxicity, as measured by reduced nuclear count, starting at 24hrs post-treatment and increasing over time (S8 Fig).

To assess whether the C16 PKRi mediated reduction in foci occurs before (and thus is likely independent of) toxicity or a consequence of toxicity in our system, the impact of AllStars death siRNA, a proprietary cocktail of siRNA sequences designed to induce cell death was tested. A time-dependent increase in cell death was observed with AllStars death siRNA treatment in both DM1_500CTG_ HF and DM1_2000CTG_ HF (S9A and S9C Fig, respectively). There is a similar time-dependent reduction in foci number in DM1_500CTG_ HF (S9B Fig). In DM1_2000CTG_ HF, foci are also reduced, but only at the 72hr time-point. These data suggest that the reduction in foci mediated by cytotoxicity follows the time-of the first observable cell death. In contrast, C16 PKRi mediated foci reduction preceded the observable cell death (S8 Fig). Taken together, we concluded that the reduction in foci mediated by C16 PKRi was likely not related to toxicity, although the only foci disrupting concentration of 10μM observed in our DM1 fibroblast model is both a significant excess of PKR inhibiting concentrations (IC50 0.21uM [28]) as well as ultimately cytotoxic at a later time-point. In keeping with the likely off-target mechanism of C16 mediated foci reduction in our system, no appreciable reduction of foci was seen with the PKR RNAi used in our screen (S1 Table). We also confirmed that PACT knockdown did not reduce cell viability (S10 Fig) and, thus, PACT siRNA-mediated reduction in foci (Fig 2) was not due to cytotoxic effects.

## Discussion

We report here the decrease of both the size and number of intranuclear RNA foci in DM1 patient fibroblasts with the siRNA-mediated reduction of the kinase modulator PACT. In addition, an increase in cellular MBNL1 protein levels is observed with PACT reduction.

While DMPK mRNA reduction has been implicated as the mechanism for a number of DM1 foci disaggregators [29], PACT does not reduce DMPK mRNA (S4 Fig) leaving the exact mechanism of foci reduction unclear. PACT is known to interact with and activate PKR during cellular stress, both inducing diverse genes as well as leading to apoptosis in some cellular contexts [30]. PACT also binds with TRBP (**t**ransactivation **r**esponse RNA (TAR) **b**inding **p**rotein) during normal conditions; cellular stress causes PACT-TRBP dissociation, allowing PACT to activate PKR by phosphorylation [22]. PKR has been previously implicated with DM1 as a protein which potentially interacts with CUG expanded mRNAs [24]. Tian and colleagues (2000) found that expression of expanded CUG repeat mRNAs formed hairpin secondary structures that had the ability to activate PKR. Furthermore, they demonstrated that CUG repeats greater than 15 triplets in length bind to PKR through its dsRNA-binding domain *in vitro*, thereby activating PKR. Activated PKR was therefore proposed to confer the cellular stress observed in DM1. The exact role that PKR plays in DM1 is still not well understood, however. PKR knockout mice crossed with a DM1 mouse model resulted in PKR KO/DM1 offspring that displayed no changes in disease development, calling into question the significance of the interaction between DMPK mRNA and PKR in DM1 [31].

A role for kinase signalling in DM1 foci integrity has been previously suggested; reduction of the size and number of foci was mediated by two small molecule ATP site directed kinase inhibitors, C51 and C16, which inhibit PKR as well as other kinases [27]. In the report, the compounds reduced nuclear RNA foci at 1uM for C16 and 30uM for C51, but did not directly inhibit binding of MBNL1 to the dsRNA, although dispersed MBNL1 and DMPK transcripts were observed. A modest reduction of PKR in DM1 cells was observed following PACT siRNA treatment although no impact on phospho-PKR levels was evident (S7 Fig). We were able to show a similar attenuation of foci in our fibroblasts treated with C16 across a 0.1nM to 10uM dose range, but only at the highest dose of 10μM where significant cellular toxicity was observed (Fig 5), 10-fold higher than the effective dose of 1μM previously reported. The reported IC50 of C16 for PKR, the concentration at which 50% PKR inhibition is attained, ranges from 141nM [32] to 210nM [28]; at 10μM (about 50-fold greater than the IC50), there is almost certainly off-target activity which may account for the foci reduction, such as the reported inhibition of cyclin dependent kinases in neurons treated with 10μM PKRi [33]. Even at the previously reported dose of 1μM for foci reduction [27] (approximately five-fold above the IC50), off-target effects have been reported–in silico predictions and in vitro kinase assays showed 93% inhibition of FGFR2 with 1μM C16 PKRi (FGFR2 IC50 = 31.8nM), which is higher than the reported 88% inhibition of the intended target, PKR (IC50 141nM); C16 PKRi also has inhibitory capacity against Aurora A (IC50 = 114nM), FGFR3 (IC50 = 478nM) and RET (33.8nM) [32]. In keeping with C16 mediated foci disruption being an off-target effect whether uniquely or combined with PKR inhibition, single-gene knockdown of PKR did not emerge as a hit in the kinome screen (S1 Table, PRKR).

Using a system very similar to the one described in our paper, Ketley *et al*. (2014) screened several libraries of small molecule compounds, including phosphatase and kinase inhibitors for modulators of nuclear foci in DM1 fibroblasts and converted myoblasts [34]. The PKC inhibitor Ro 31–8220 was found to reduce RNA foci, redistribute MBNL1 protein to the cytoplasm and normalize alternative splicing of IR and ATP2B1 (or SERCA1). Although Ro 31–8220 was previously identified as a PKC inhibitor, the authors demonstrate that the compound’s impact on DM1 pathomechanism is independent of PKC, suggesting involvement of other kinases or even a non-kinase role. Ro 31–8220 treatment completely abolished foci compared to the 50% reduction mediated by PACT knockdown (Fig 2); no kinase knocked down in our screen approximated this effect of Ro 31–8220. This discrepancy might be explained by more than one kinase being targeted by RO31-8220 in contrast to our screen which targets only single kinases. In this regard, one major limitation of a kinome siRNA screen is that the outcome is dependent on single-kinase regulators of foci. The data from the kinome siRNA screens suggest that, at least within the human kinome, there exists no single master regulator of foci capable of correcting the disease-associated spliceopathies. This single-gene interrogation approach does not address the possibility of combination knockdown of multiple kinases for therapeutic efficacy in DM1; this is especially important to consider given the evidence for redundancy in the kinome with overlapping or compensatory roles within a group of kinases [35]. The redundancy of the kinome may prevent a profound foci reduction from occurring.

The increase in MBNL1 protein level observed with the RNAi mediated PACT reduction in DM1 fibroblasts (Fig 3) was somewhat unanticipated given that while there may be shift in cellular compartmentalization of MBNL1 from foci to the nucleoplasm, total cellular MBNL1 would not be expected to change. Interestingly, an increase in MBNL1, albeit not to the same degree, is also observed in control HF with PACT knockdown, suggesting the mechanism is not wholly dependent on the presence of expanded CUG hairpins (Fig 3A). Immunofluorescent localization of MBNL1 with PACT siRNA revealed the anticipated reduction of MBNL1 positive foci in DM1 cells but also increases in nuclear vs cytosolic MBNL1 fractions (Fig 3E and 3F). Cytosolic translocation of MBNL1 has recently been shown to be contingent on ubiquitination in neuronal cells [36]. Given PACT has been shown to mediate ubiquitination of the tumor suppressor p53 [37], it is possible a similar mechanism occurs here with diminution of MBNL1 ubiquitination resulting in reduced cytoplasmic levels of the protein.

The PACT-mediated decrease in foci levels and increase in MBNL1 protein had only a mild impact on alternative splicing of SERCA1 which did not reach statistical significance; no impact was seen on IR splicing (Fig 4). In animal models over-expressing MBNL1, there was an 8–16 fold induction of the protein [17] and thus, a 2–3 fold induction seen here may not be sufficient to change alternative splicing. Further investigation is required to delineate the minimum and maximum thresholds of MBNL1 overexpression *in vitro* and *in vivo* in order to observe MBNL1-mediated correction of DM1 pathology without reaching toxicity.

The specific disposition of PACT is unknown, in particular whether it binds to the DMPK mRNA nuclear foci is unclear. Neither it nor any of its known protein binding partners have been previously shown to be present in foci, although thorough proteomic analyses of foci make up have not been published. And although PACT has three double stranded RNA binding protein domains, the protein has never been shown to actually bind RNA itself [38], while PACT knockdown alone is not sufficient to elicit profound splicing correction alone, it may offer a novel target pathway for drug development as an adjunct to other potential therapies, such as Ro 31–8220, C16, and C51.

## Supporting information

S1 Table Z-score data for nuclei count, foci count and foci area from a partial kinome RNAi screen in DM1_500 CTG_ HF. The screen was done in triplicate using 10nM of pooled siRNA with an end-point readout conducted at 96 hrs; due to technical errors, this original screen included only a portion of the entire kinome RNAi library. The data was normalized to the sample mean per plate and is represented as average Z-score values.
(PDF)

S2 Table Columbus image algorithm used for RNA foci image analysis. (TIF)

S3 Table Columbus image algorithm used for MBNL1 image analysis in Fig 3E and 3F. (TIF)

S1 Fig CUG-RNA foci are unique biomarkers in DM1 patient cells and are absent in unaffected control cells. (A) Representative images of nuclear foci (blue–Hoechst; green–CUG foci) after RNA FISH of CUG foci in control and patient skin fibroblasts. Quantification of (B) number of foci per nucleus and (C) total foci area per nucleus using means data from five replicate wells (n = 5; two-way ANOVA; errors represent SD).
(TIF)

S2 Fig Validation of PACT knockdown as a novel modulator of DM1 foci in DM1_500CTG_ HF. (A) Western blot analysis of PACT knockdown using individual and pooled PACT siRNA. (B) Quantification of PACT knockdown in (A) was done by densitometric analysis using Image J. (C-D) Foci integrity upon PACT knockdown. Cells were probed with Alexa-555 (CAG)_10_ probes to detect and image foci. Quantification of (C) number of foci per nucleus and (D) total foci area per nucleus was done using the Columbus software and mean data from five replicate wells is shown (n = 5; two-way ANOVA; error bars represent SD). *P < 0.05, **P < 0.01, ***P< 0.001.
(TIF)

S3 Fig PACT protein is reduced in both DM1_500 CTG_ and DM1_2000 CTG_ cells with PACT siRNA treatment over a 72hr time-course. Western blot analysis of PACT protein levels upon knockdown using PACT siRNA in (A) DM1_500 CTG_ and (B) DM1_2000 CTG_ patient fibroblasts, respectively.
(TIF)

S4 Fig PACT knockdown is confirmed but DMPK and MBNL1 transcript are unchanged in cDNA samples used for SERCA1 and IR splicing analysis. RT-qPCR analysis of (A and D) DMPK, (B and E) PACT, and (C and F) MBNL1 transcript levels in DM1_500CTG_ and DM1_2000CTG_ HF, respectively, after a 72hr treatment with PACT siRNA; target genes were normalized to GAPDH and HPRT1 as reference genes. Samples were run in duplicates for each gene with replicates of n = 5.
(TIF)

S5 Fig Assessment of DM1 spliceopathy by RT-qPCR compared to Control HF. (A) SERCA1 and (B) IR splicing patterns in Control HF versus DM1 HF using RT-qPCR. Error bars are representative of duplicate qPCR runs, SEM. (A) SERCA1-A levels were measured using an exon 22-specific primer and quantified against total SERCA1-A and B as the reference gene. (B) IR-B mRNA levels were measured using an exon 11-specific primer and quantified against total IR-A and B as the reference gene. MBNL1 knockdown was used to validate sensitivity of the assay in detecting changes in splicing upon treatment. (C) MBNL1 with reference to GAPDH, (D) SERCA1-A with reference to SERCA1-AB, and (E) IR-B with reference to IR-AB with measured in duplicate qPCR runs.
(TIF)

S6 Fig MBNL1 is induced and SERCA1-A slightly increased with PACT knockdown in Control HF. (A) Western blot analysis of MBNL1 protein levels upon PACT knockdown and (B) the corresponding quantification using Image J software (n = 3; unpaired t-test; error bars represent SD). MBNL1 protein level was normalized to HSP90 loading control and the normalized values were averaged across trials. (C) PACT mRNA, (D) MBNL1 mRNA, and (E) DMPK mRNA, all three with reference to GAPDH and HPRT1; (F) SERCA1-A with reference to SERCA1-AB; and (G) IR-B with reference to IR-AB were measured in duplicate qPCR runs per sample. The non-targeting siRNA treated sample in one trial was set as the “control” sample and all other sample, non-targeting siRNA and PACT siRNA treated sample from all other trials, were quantified relative to this “control” (n = 5; unpaired t-test; error bars represent SD).
(TIF)

S7 Fig PKR Levels are lower in DM1 cells withand phosphorylation status following PACT RNAi knockdown. PACT, PKR and phospho-PKR (Tyrosine 446) levels following PACT siRNA in HF (A), DM1_500 CTG_ (B) and DM1_2000 CTG_ (C).
(TIF)

S8 Fig C16 PKRi kills cells at high dose (10,000nM). Small molecule PKR inhibitor C16 was used to treat (A) DM1-500_500CTG_ HF or (B) DM1_2000cTG_ HF at doses ranging from 0.1nM-10,000nM for 8hrs up to 168hrs (7 days) following which the nucleus was stained with Hoechst. Number of nuclei per well was quantified using Columbus software (n = 3; two-way ANOVA; error bars represent SD).
(TIF)

S9 Fig AllStars death siRNA kills cells in a time-dependent manner and reduces nuclear foci. Effect of AllStars death siRNA on number of cells (A, C) and number of foci per nucleus (B, D) in DM1_500 CTG_ and DM1_2000 CTG_, respectively. Cells were stained with Hoechst to visualize the nuclei and probed with Alexa-555 (CAG)_10_ probes to detect foci. Quantification was done using the Columbus software (n = 4; two-way ANOVA; error bars represent SD). For statistical analysis, each trial consisted of mean data from 3–9 replicate wells per sample with corresponding SD. The data was converted to fold change relative to non-targeting siRNA to accommodate plate-to-plate variability and the SD was adjusted to the same ratio. The fold change and SD data were averaged across all trials.
(TIF)

S10 Fig PACT knockdown is not toxic to DM1 fibroblasts. Effect of PACT siRNA treatment on number of cells in (A) DM1_500 CTG_ and (B) DM1_2000 CTG_ HF. Cells were stained with Hoechst to visualize the nuclei. Quantification was done using the Columbus software (n = 4; two-way ANOVA; error bars represent SD). For statistical analysis, each trial consisted of mean data from 3–9 replicate wells per sample with corresponding SD. The data was converted to fold change relative to non-targeting siRNA to accommodate plate-to-plate variability and the SD was adjusted to the same ratio. The fold change and SD data were averaged across all trials.
(TIF)

S1 File (PDF)
