# Supplementary material for: High-throughput kinome-RNAi screen identifies protein kinase R activator (PACT) as a novel genetic modifier of CUG foci integrity in myotonic dystrophy type 1 (DM1)
Source: PLoS One. 2021 Sep 14;16(9):e0256276. doi: 10.1371/journal.pone.0256276 (PMC8439471; doi:10.1371/journal.pone.0256276)
Supplement: S1 Table — The screen was done in triplicate using 10nM of pooled siRNA with an end-point readout conducted at 96 hrs; due to technical errors, this original screen included only a portion of the entire kinome RNAi library. The data was normalized to the sample mean per plate and is represented as average Z-score values. (PDF) [file pone.0256276.s001.pdf]

| siRNA | Plate | Well Name | Row | Column | Z-score (nuclei count) | Z-score (foci count) | Z-score (foci area) |
|---|---|---|---|---|---|---|---|
| PRKAA1 | hKQsi002 | B3 | 2 | 3 | 0.05 | 1.51 | 1.53 |
| PRKCA | hKQsi002 | B4 | 2 | 4 | 0.58 | 0.19 | 0.47 |
| PRKG2 | hKQsi002 | B5 | 2 | 5 | -0.03 | 0.29 | 0.45 |
| MAP2K5 | hKQsi002 | B6 | 2 | 6 | 0.40 | 0.51 | 0.38 |
| RAGE | hKQsi002 | B7 | 2 | 7 | 0.24 | -0.72 | -0.24 |
| SGK | hKQsi002 | B8 | 2 | 8 | -1.41 | -0.31 | -0.44 |
| AURKA | hKQsi002 | B10 | 2 | 10 | 0.36 | -0.39 | -0.72 |
| TK2 | hKQsi002 | B11 | 2 | 11 | -0.46 | 0.10 | 0.18 |
| MAP3K12 | hKQsi002 | B12 | 2 | 12 | -0.42 | 0.25 | 0.03 |
| IKBKG | hKQsi002 | B13 | 2 | 13 | -0.44 | -0.98 | -0.62 |
| CDC2L5 | hKQsi002 | B14 | 2 | 14 | 0.12 | -0.73 | -0.71 |
| CDK5R2 | hKQsi002 | B15 | 2 | 15 | -0.57 | -0.11 | -1.22 |
| DGKI | hKQsi002 | B17 | 2 | 17 | -1.32 | -1.38 | -0.70 |
| AKAP6 | hKQsi002 | B18 | 2 | 18 | 0.01 | 0.26 | -0.11 |
| ARK5 | hKQsi002 | B19 | 2 | 19 | 1.48 | 0.14 | -0.26 |
| PAK4 | hKQsi002 | B20 | 2 | 20 | -0.03 | -1.07 | -0.46 |
| NEK6 | hKQsi002 | B21 | 2 | 21 | -0.46 | -0.33 | -0.36 |
| AKAP11 | hKQsi002 | B22 | 2 | 22 | 0.89 | -1.36 | -1.08 |
| PRKAA2 | hKQsi002 | C3 | 3 | 3 | 0.39 | 0.74 | 0.35 |
| PRKCB1 | hKQsi002 | C4 | 3 | 4 | 0.51 | 0.54 | 0.28 |
| MAPK1 | hKQsi002 | C5 | 3 | 5 | -1.13 | 0.42 | 0.23 |
| MAP2K6 | hKQsi002 | C6 | 3 | 6 | -0.02 | -0.51 | -0.40 |
| RAF1 | hKQsi002 | C7 | 3 | 7 | 0.44 | 0.30 | 0.25 |
| SKP1A | hKQsi002 | C8 | 3 | 8 | -0.06 | -0.23 | -0.04 |
| CDKL5 | hKQsi002 | C10 | 3 | 10 | 0.94 | -1.17 | -0.91 |
| TRIO | hKQsi002 | C11 | 3 | 11 | 0.14 | -0.35 | -0.32 |
| MAPKAPK3 | hKQsi002 | C12 | 3 | 12 | -0.68 | 1.58 | 1.41 |
| IKBKAP | hKQsi002 | C13 | 3 | 13 | -0.44 | -0.62 | -0.93 |
| MAP2K1IP1 | hKQsi002 | C14 | 3 | 14 | -0.94 | -1.28 | -0.32 |
| RPS6KA4 | hKQsi002 | C15 | 3 | 15 | -0.41 | 0.85 | -0.16 |
| MAP3K13 | hKQsi002 | C17 | 3 | 17 | -0.77 | -1.11 | -0.54 |
| ROCK2 | hKQsi002 | C18 | 3 | 18 | -0.62 | -0.58 | -0.35 |
| XYLB | hKQsi002 | C19 | 3 | 19 | 0.26 | -0.64 | -0.52 |
| TESK2 | hKQsi002 | C20 | 3 | 20 | 1.65 | -1.32 | -1.13 |
| FASTK | hKQsi002 | C21 | 3 | 21 | 0.06 | -0.11 | 0.03 |

| | | | | | | | |
|---|---|---|---|---|---|---|---|
| AKAP10 | hKQsi002 | C22 | 3 | 22 | 4.73 | -0.17 | -0.46 |
| PRKAB1 | hKQsi002 | D3 | 4 | 3 | 1.19 | -0.45 | -0.66 |
| PRKCD | hKQsi002 | D4 | 4 | 4 | -0.24 | -0.88 | -1.26 |
| MAPK3 | hKQsi002 | D5 | 4 | 5 | 3.08 | 0.79 | 0.36 |
| MAP2K7 | hKQsi002 | D6 | 4 | 6 | -0.77 | -0.48 | -0.31 |
| RET | hKQsi002 | D7 | 4 | 7 | -0.34 | -0.04 | -0.02 |
| SKP2 | hKQsi002 | D8 | 4 | 8 | -1.46 | 0.25 | 0.81 |
| STK10 | hKQsi002 | D10 | 4 | 10 | -0.11 | 0.46 | 0.34 |
| TTK | hKQsi002 | D11 | 4 | 11 | -2.37 | 0.94 | 1.10 |
| AKAP1 | hKQsi002 | D12 | 4 | 12 | -1.32 | -0.02 | -0.42 |
| DGKZ | hKQsi002 | D13 | 4 | 13 | 0.44 | -1.32 | -1.36 |
| TNK1 | hKQsi002 | D14 | 4 | 14 | 0.77 | 0.34 | 0.10 |
| CDKL2 | hKQsi002 | D15 | 4 | 15 | 0.35 | -0.35 | -0.90 |
| DCAMKL1 | hKQsi002 | D17 | 4 | 17 | 0.60 | 0.58 | 0.46 |
| MAPK8IP1 | hKQsi002 | D18 | 4 | 18 | -0.11 | -1.11 | -0.80 |
| OSR1 | hKQsi002 | D19 | 4 | 19 | 1.38 | -0.57 | -1.18 |
| MAP3K7IP1 | hKQsi002 | D20 | 4 | 20 | -0.80 | -0.08 | 0.82 |
| ASK | hKQsi002 | D21 | 4 | 21 | -0.36 | 0.08 | 0.27 |
| PACSIN2 | hKQsi002 | D22 | 4 | 22 | -0.25 | 0.09 | 1.00 |
| PRKAB2 | hKQsi002 | E3 | 5 | 3 | 2.10 | 1.18 | 0.98 |
| PRKCE | hKQsi002 | E4 | 5 | 4 | -0.46 | -0.76 | -0.80 |
| MAPK4 | hKQsi002 | E5 | 5 | 5 | 1.21 | 1.50 | 0.69 |
| PRKR | hKQsi002 | E6 | 5 | 6 | -0.90 | -0.60 | -0.20 |
| GRK1 | hKQsi002 | E7 | 5 | 7 | 1.26 | -0.96 | -0.75 |
| SRC | hKQsi002 | E8 | 5 | 8 | 0.25 | -0.15 | 0.37 |
| STK11 | hKQsi002 | E10 | 5 | 10 | -0.32 | 0.48 | 0.47 |
| TXK | hKQsi002 | E11 | 5 | 11 | -0.55 | -0.69 | -0.46 |
| NME5 | hKQsi002 | E12 | 5 | 12 | -1.03 | 0.54 | 0.11 |
| DGKE | hKQsi002 | E13 | 5 | 13 | 0.42 | 0.33 | 0.30 |
| RIPK1 | hKQsi002 | E14 | 5 | 14 | -0.64 | -0.36 | 0.20 |
| MAP3K14 | hKQsi002 | E15 | 5 | 15 | -0.99 | -0.61 | -0.55 |
| AURKB | hKQsi002 | E17 | 5 | 17 | -1.03 | 0.71 | 0.99 |
| AKAP5 | hKQsi002 | E18 | 5 | 18 | 0.06 | -0.27 | -0.45 |
| AKT3 | hKQsi002 | E19 | 5 | 19 | 1.13 | 0.63 | 0.60 |
| MERTK | hKQsi002 | E20 | 5 | 20 | -1.20 | -1.12 | -1.25 |
| TLK2 | hKQsi002 | E21 | 5 | 21 | 0.12 | -2.24 | -1.69 |
| STK38 | hKQsi002 | E22 | 5 | 22 | 1.72 | 0.51 | -0.09 |
| PRKACA | hKQsi002 | F3 | 6 | 3 | 1.55 | 0.19 | -0.24 |
| PRKCG | hKQsi002 | F4 | 6 | 4 | 0.00 | 0.13 | 0.13 |
| MAPK6 | hKQsi002 | F5 | 6 | 5 | 0.40 | 0.48 | 0.44 |

| | | | | | | | |
|---|---|---|---|---|---|---|---|
| PRKRIR | hKQsi002 | F6 | 6 | 6 | -1.10 | 0.47 | 0.40 |
| ROCK1 | hKQsi002 | F7 | 6 | 7 | 0.52 | -1.17 | -0.96 |
| SRMS | hKQsi002 | F8 | 6 | 8 | 0.67 | 0.83 | 0.55 |
| AURKC | hKQsi002 | F10 | 6 | 10 | -0.06 | -0.83 | -0.85 |
| TYK2 | hKQsi002 | F11 | 6 | 11 | -0.27 | 0.42 | 0.25 |
| PIP5K1A | hKQsi002 | F12 | 6 | 12 | 0.58 | -0.15 | -0.34 |
| DGKD | hKQsi002 | F13 | 6 | 13 | 0.60 | 0.49 | 0.31 |
| RIPK2 | hKQsi002 | F14 | 6 | 14 | -0.38 | 1.24 | 0.83 |
| STK29 | hKQsi002 | F15 | 6 | 15 | -1.42 | -1.00 | -0.47 |
| RPS6KA5 | hKQsi002 | F17 | 6 | 17 | -0.63 | -1.21 | -1.12 |
| CDC42BPB | hKQsi002 | F18 | 6 | 18 | -0.63 | 0.24 | 0.32 |
| GNE | hKQsi002 | F19 | 6 | 19 | -0.54 | 0.51 | 0.58 |
| STK25 | hKQsi002 | F20 | 6 | 20 | 0.50 | -1.14 | -0.88 |
| RIPK3 | hKQsi002 | F21 | 6 | 21 | 2.23 | -1.14 | -0.91 |
| PTK9L | hKQsi002 | F22 | 6 | 22 | -0.96 | -0.68 | -0.54 |
| PRKACB | hKQsi002 | G3 | 7 | 3 | -1.23 | -0.02 | 0.70 |
| PRKCH | hKQsi002 | G4 | 7 | 4 | 1.21 | -0.28 | -0.66 |
| MAPK7 | hKQsi002 | G5 | 7 | 5 | 0.23 | 1.04 | 0.49 |
| PRKX | hKQsi002 | G6 | 7 | 6 | 0.28 | -1.69 | -0.69 |
| ROS1 | hKQsi002 | G7 | 7 | 7 | -0.89 | 0.97 | 0.87 |
| SRPK1 | hKQsi002 | G8 | 7 | 8 | -1.47 | -0.12 | -0.15 |
| SYK | hKQsi002 | G10 | 7 | 10 | -0.78 | -0.96 | -0.79 |
| TYRO3 | hKQsi002 | G11 | 7 | 11 | 0.37 | 0.36 | -0.01 |
| PIP5K1B | hKQsi002 | G12 | 7 | 12 | 0.72 | -0.71 | -0.69 |
| CAMK1 | hKQsi002 | G13 | 7 | 13 | -0.60 | 0.66 | 0.85 |
| RIOK3 | hKQsi002 | G14 | 7 | 14 | -0.35 | 0.31 | -0.04 |
| MAP3K6 | hKQsi002 | G15 | 7 | 15 | 2.31 | -0.92 | -0.91 |
| MAPKAPK2 | hKQsi002 | G17 | 7 | 17 | -0.28 | -0.13 | -0.68 |
| AKAP12 | hKQsi002 | G18 | 7 | 18 | -0.37 | -0.78 | -0.43 |
| SGK2 | hKQsi002 | G19 | 7 | 19 | 0.59 | 1.77 | -0.37 |
| CIB2 | hKQsi002 | G20 | 7 | 20 | -1.64 | 0.03 | -0.14 |
| PIM2 | hKQsi002 | G21 | 7 | 21 | -0.28 | -0.66 | -0.94 |
| AAK1 | hKQsi002 | G22 | 7 | 22 | -0.45 | -0.14 | 0.04 |
| PRKACG | hKQsi002 | H3 | 8 | 3 | -0.85 | -0.17 | -0.35 |
| PRKCI | hKQsi002 | H4 | 8 | 4 | -0.43 | 3.19 | 1.72 |
| MAPK8 | hKQsi002 | H5 | 8 | 5 | -0.74 | -0.15 | 0.42 |
| PRKY | hKQsi002 | H6 | 8 | 6 | -0.92 | 0.19 | 0.48 |
| RPS6KA1 | hKQsi002 | H7 | 8 | 7 | -0.02 | 2.77 | 1.70 |
| SRPK2 | hKQsi002 | H8 | 8 | 8 | -0.08 | -0.89 | -0.25 |
| MAP3K7 | hKQsi002 | H10 | 8 | 10 | 0.68 | 0.47 | -0.08 |

| | | | | | | | |
|---|---|---|---|---|---|---|---|
| TYROBP | hKQsi002 | H11 | 8 | 11 | -0.22 | 0.90 | 0.39 |
| PIP5K2B | hKQsi002 | H12 | 8 | 12 | -0.03 | -1.11 | -1.18 |
| MAPKAPK5 | hKQsi002 | H13 | 8 | 13 | -0.27 | 0.70 | 0.27 |
| DYRK4 | hKQsi002 | H14 | 8 | 14 | -1.46 | -0.05 | 0.25 |
| PKMYT1 | hKQsi002 | H15 | 8 | 15 | 0.62 | -0.21 | 0.38 |
| STK17B | hKQsi002 | H17 | 8 | 17 | 0.51 | -0.65 | -0.61 |
| AATK | hKQsi002 | H18 | 8 | 18 | -1.57 | -0.43 | 1.30 |
| HIPK3 | hKQsi002 | H19 | 8 | 19 | -0.42 | 0.69 | -0.36 |
| AKAP3 | hKQsi002 | H20 | 8 | 20 | 0.00 | -0.44 | -0.16 |
| CIT | hKQsi002 | H21 | 8 | 21 | -0.38 | 0.68 | 0.72 |
| LMTK2 | hKQsi002 | H22 | 8 | 22 | 1.03 | -0.87 | -0.82 |
| PKIA | hKQsi002 | I3 | 9 | 3 | 0.54 | 0.16 | -0.07 |
| PRKCL1 | hKQsi002 | I4 | 9 | 4 | -0.02 | 0.09 | -0.03 |
| MAPK11 | hKQsi002 | I5 | 9 | 5 | 2.04 | 1.10 | 0.77 |
| PRSS7 | hKQsi002 | I6 | 9 | 6 | 0.12 | 0.70 | 0.23 |
| RPS6KA2 | hKQsi002 | I7 | 9 | 7 | 0.51 | -0.39 | -0.21 |
| NEK4 | hKQsi002 | I8 | 9 | 8 | -0.66 | 0.55 | -0.25 |
| TEC | hKQsi002 | I10 | 9 | 10 | 0.39 | 1.56 | 1.11 |
| UGP2 | hKQsi002 | I11 | 9 | 11 | -0.27 | 0.42 | 1.14 |
| ULK1 | hKQsi002 | I12 | 9 | 12 | 0.43 | -0.16 | -0.33 |
| CDK10 | hKQsi002 | I13 | 9 | 13 | 0.28 | -1.27 | -1.13 |
| CDKL1 | hKQsi002 | I14 | 9 | 14 | -0.21 | 0.71 | 0.02 |
| LATS1 | hKQsi002 | I15 | 9 | 15 | 1.19 | 0.02 | -0.17 |
| STK17A | hKQsi002 | I17 | 9 | 17 | 0.35 | -1.54 | -1.29 |
| IKBKE | hKQsi002 | I18 | 9 | 18 | 0.73 | -0.03 | -0.42 |
| AKAP9 | hKQsi002 | I19 | 9 | 19 | 0.58 | 0.25 | -0.65 |
| ERN2 | hKQsi002 | I20 | 9 | 20 | -0.86 | 0.65 | 1.06 |
| KALRN | hKQsi002 | I21 | 9 | 21 | 0.38 | 0.57 | 3.23 |
| ICK | hKQsi002 | I22 | 9 | 22 | -0.21 | 0.68 | 0.28 |
| PKIB | hKQsi002 | J3 | 10 | 3 | 0.03 | -0.07 | -0.57 |
| PRKCL2 | hKQsi002 | J4 | 10 | 4 | -0.32 | -1.34 | -1.08 |
| MAPK9 | hKQsi002 | J5 | 10 | 5 | -0.87 | 0.51 | 1.80 |
| PSKH1 | hKQsi002 | J6 | 10 | 6 | 0.41 | -0.72 | -0.46 |
| RPS6KA3 | hKQsi002 | J7 | 10 | 7 | -0.15 | -0.22 | -0.46 |
| STK3 | hKQsi002 | J8 | 10 | 8 | -0.95 | -0.84 | -0.78 |
| TEK | hKQsi002 | J10 | 10 | 10 | 0.36 | -0.44 | -0.19 |
| UMPK | hKQsi002 | J11 | 10 | 11 | -0.46 | -0.79 | -0.74 |
| STK24 | hKQsi002 | J12 | 10 | 12 | 1.52 | -0.22 | 0.04 |
| PDXK | hKQsi002 | J13 | 10 | 13 | -0.34 | -0.46 | -1.08 |
| KSR | hKQsi002 | J14 | 10 | 14 | -0.21 | 0.95 | 1.24 |

| | | | | | | | |
|---|---|---|---|---|---|---|---|
| HGS | hKQsi002 | J15 | 10 | 15 | -1.52 | 0.55 | -0.24 |
| TAO1 | hKQsi002 | J17 | 10 | 17 | 0.73 | 1.19 | 0.69 |
| ULK2 | hKQsi002 | J18 | 10 | 18 | -1.42 | 0.39 | 0.75 |
| TNK2 | hKQsi002 | J19 | 10 | 19 | -0.54 | -0.82 | -1.11 |
| PDLIM5 | hKQsi002 | J20 | 10 | 20 | 0.23 | -0.54 | -0.44 |
| PKIG | hKQsi002 | J21 | 10 | 21 | -0.37 | -1.71 | -1.46 |
| SORCS3 | hKQsi002 | J22 | 10 | 22 | -0.52 | -0.41 | -0.41 |
| PRKAG1 | hKQsi002 | K3 | 11 | 3 | 1.76 | 1.91 | 1.13 |
| PRKCM | hKQsi002 | K4 | 11 | 4 | 0.62 | -0.56 | -0.72 |
| MAPK10 | hKQsi002 | K5 | 11 | 5 | 0.69 | 1.85 | 1.22 |
| PTK2 | hKQsi002 | K6 | 11 | 6 | 0.03 | 0.94 | 0.58 |
| RPS6KB1 | hKQsi002 | K7 | 11 | 7 | 1.09 | -0.91 | -0.99 |
| STK4 | hKQsi002 | K8 | 11 | 8 | 0.33 | -0.41 | 0.41 |
| TESK1 | hKQsi002 | K10 | 11 | 10 | 0.06 | 0.80 | 1.50 |
| VRK1 | hKQsi002 | K11 | 11 | 11 | -1.01 | 0.40 | -0.11 |
| DYRK3 | hKQsi002 | K12 | 11 | 12 | 2.46 | -0.20 | -0.06 |
| MADD | hKQsi002 | K13 | 11 | 13 | 0.33 | 0.36 | -0.01 |
| CDK5R1 | hKQsi002 | K14 | 11 | 14 | -0.77 | 0.34 | 0.42 |
| DYRK1B | hKQsi002 | K15 | 11 | 15 | -0.06 | -0.90 | -0.39 |
| TAO1 | hKQsi002 | K17 | 11 | 17 | 0.39 | 0.66 | 1.09 |
| SLK | hKQsi002 | K18 | 11 | 18 | -0.15 | -0.03 | -0.06 |
| NME6 | hKQsi002 | K19 | 11 | 19 | 0.75 | 0.41 | -0.63 |
| CAMKK2 | hKQsi002 | K20 | 11 | 20 | 0.07 | 0.34 | 0.57 |
| MAP4K5 | hKQsi002 | K21 | 11 | 21 | 0.36 | -1.05 | -0.96 |
| MAPKBP1 | hKQsi002 | K22 | 11 | 22 | 0.89 | -0.44 | -0.51 |
| PRKAR1A | hKQsi002 | L3 | 12 | 3 | -1.78 | -0.58 | -1.00 |
| PRKCQ | hKQsi002 | L4 | 12 | 4 | 0.46 | 0.23 | -0.26 |
| MAPK13 | hKQsi002 | L5 | 12 | 5 | -0.46 | 0.44 | 0.79 |
| PTK6 | hKQsi002 | L6 | 12 | 6 | -0.57 | 0.48 | 0.28 |
| RPS6KB2 | hKQsi002 | L7 | 12 | 7 | 0.24 | -0.47 | -0.73 |
| TGFBR1 | hKQsi002 | L10 | 12 | 10 | 1.38 | 0.53 | 0.17 |
| VRK2 | hKQsi002 | L11 | 12 | 11 | -0.48 | 1.43 | 0.95 |
| DYRK2 | hKQsi002 | L12 | 12 | 12 | 0.35 | 0.27 | 0.48 |
| MKNK1 | hKQsi002 | L13 | 12 | 13 | -2.00 | -0.82 | -0.07 |
| AKAP4 | hKQsi002 | L14 | 12 | 14 | -0.76 | 0.63 | -0.09 |
| MAP4K4 | hKQsi002 | L17 | 12 | 17 | 0.66 | -0.25 | 1.74 |
| IHPK1 | hKQsi002 | L18 | 12 | 18 | 0.48 | 2.87 | 1.24 |
| CNKSR1 | hKQsi002 | L19 | 12 | 19 | 0.32 | 0.05 | -0.13 |
| PMVK | hKQsi002 | L20 | 12 | 20 | 0.44 | -1.10 | -0.57 |
| MAP4K1 | hKQsi002 | L21 | 12 | 21 | 0.57 | 2.69 | 2.05 |

| | | | | | | | |
|---|---|---|---|---|---|---|---|
| PRKAR1B | hKQsi002 | M3 | 13 | 3 | 0.93 | 0.66 | 0.71 |
| PRKCZ | hKQsi002 | M4 | 13 | 4 | 1.10 | 1.38 | 1.02 |
| MAP2K1 | hKQsi002 | M5 | 13 | 5 | -0.11 | 0.57 | 0.68 |
| PTK7 | hKQsi002 | M6 | 13 | 6 | -1.13 | 0.62 | 0.41 |
| RYK | hKQsi002 | M7 | 13 | 7 | -0.30 | -1.74 | -1.00 |
| TGFBR2 | hKQsi002 | M10 | 13 | 10 | -0.13 | -1.11 | -0.77 |
| WEE1 | hKQsi002 | M11 | 13 | 11 | -2.47 | -0.25 | -0.12 |
| CDC42BPA | hKQsi002 | M12 | 13 | 12 | 0.35 | 2.13 | 1.28 |
| CASK | hKQsi002 | M13 | 13 | 13 | 0.33 | 1.27 | 1.15 |
| STK19 | hKQsi002 | M14 | 13 | 14 | 0.34 | 0.17 | -0.15 |
| EIF2AK3 | hKQsi002 | M17 | 13 | 17 | 1.05 | 1.46 | 4.28 |
| GIT2 | hKQsi002 | M18 | 13 | 18 | -0.49 | -0.35 | 0.53 |
| AKAP8 | hKQsi002 | M19 | 13 | 19 | -0.74 | -0.67 | 0.66 |
| PLK4 | hKQsi002 | M20 | 13 | 20 | 0.30 | -0.16 | 0.09 |
| CHEK2 | hKQsi002 | M21 | 13 | 21 | 0.81 | -1.58 | -1.36 |
| PRKAR2A | hKQsi002 | N3 | 14 | 3 | 1.35 | -0.07 | -0.68 |
| PRKDC | hKQsi002 | N4 | 14 | 4 | 0.68 | 0.53 | -0.08 |
| MAP2K2 | hKQsi002 | N5 | 14 | 5 | -0.31 | 0.21 | 0.48 |
| PTK9 | hKQsi002 | N6 | 14 | 6 | -0.53 | 1.06 | 0.99 |
| MAPK12 | hKQsi002 | N7 | 14 | 7 | -0.76 | -0.38 | -0.20 |
| TIE | hKQsi002 | N10 | 14 | 10 | -0.08 | -0.42 | -0.57 |
| YES1 | hKQsi002 | N11 | 14 | 11 | -0.93 | 0.45 | -0.05 |
| MAP4K3 | hKQsi002 | N12 | 14 | 12 | 1.24 | 1.66 | 0.73 |
| PRKRA | hKQsi002 | N13 | 14 | 13 | 1.49 | -1.97 | -1.95 |
| SPHK1 | hKQsi002 | N14 | 14 | 14 | -0.29 | 0.06 | 0.16 |
| PRKCABP | hKQsi002 | N17 | 14 | 17 | -1.14 | -0.27 | 0.01 |
| MELK | hKQsi002 | N18 | 14 | 18 | -1.51 | -0.91 | -0.83 |
| SPEG | hKQsi002 | N19 | 14 | 19 | -2.03 | 0.72 | 2.49 |
| MAP3K2 | hKQsi002 | N20 | 14 | 20 | -0.37 | -0.67 | 0.29 |
| IRAK3 | hKQsi002 | N21 | 14 | 21 | 0.98 | 0.13 | 0.48 |
| PRKAR2B | hKQsi002 | O3 | 15 | 3 | -0.28 | -0.31 | -0.44 |
| PRKG1 | hKQsi002 | O4 | 15 | 4 | 0.61 | -1.15 | -0.91 |
| MAP2K3 | hKQsi002 | O5 | 15 | 5 | 1.32 | 2.95 | 2.49 |
| MAP4K2 | hKQsi002 | O6 | 15 | 6 | -1.81 | 1.25 | 1.14 |
| MAP2K4 | hKQsi002 | O7 | 15 | 7 | -1.10 | -0.70 | -0.40 |
| TK1 | hKQsi002 | O10 | 15 | 10 | -2.52 | 0.16 | -0.43 |
| ZAP70 | hKQsi002 | O11 | 15 | 11 | -0.29 | -0.49 | -0.37 |
| PIK3R3 | hKQsi002 | O12 | 15 | 12 | -0.72 | -0.29 | -0.44 |
| STK16 | hKQsi002 | O13 | 15 | 13 | -0.68 | -0.65 | -0.89 |
| PRPF4B | hKQsi002 | O14 | 15 | 14 | 1.46 | 0.23 | 0.29 |

| | | | | | | | |
|---|---|---|---|---|---|---|---|
| AKAP7 | hKQsi002 | O17 | 15 | 17 | -0.16 | -1.47 | -1.14 |
| TLK1 | hKQsi002 | O18 | 15 | 18 | -0.60 | -1.62 | -0.78 |
| BCKDK | hKQsi002 | O19 | 15 | 19 | 0.14 | -0.53 | -0.11 |
| PLK2 | hKQsi002 | O20 | 15 | 20 | 0.51 | -0.13 | -0.37 |
| AKAP13 | hKQsi002 | O21 | 15 | 21 | -0.36 | -1.75 | -1.54 |
| STK38L | hKQsi003 | B3 | 2 | 3 | 0.91 | -0.19 | -0.53 |
| MAPK8IP2 | hKQsi003 | B4 | 2 | 4 | -0.04 | 0.95 | 1.17 |
| LATS2 | hKQsi003 | B5 | 2 | 5 | 0.35 | -1.58 | -0.79 |
| EEF2K | hKQsi003 | B6 | 2 | 6 | 0.85 | 0.07 | -0.14 |
| JIK | hKQsi003 | B7 | 2 | 7 | -1.12 | 0.01 | 0.52 |
| SNRK | hKQsi003 | B8 | 2 | 8 | 0.88 | -0.58 | -0.62 |
| RFK | hKQsi003 | B10 | 2 | 10 | -0.16 | 0.01 | 0.82 |
| SPHK2 | hKQsi003 | B11 | 2 | 11 | 0.08 | 0.48 | 1.28 |
| FN3K | hKQsi003 | B12 | 2 | 12 | -0.35 | 0.24 | -0.32 |
| FN3KRP | hKQsi003 | B13 | 2 | 13 | -0.67 | -0.45 | 0.29 |
| UCK1 | hKQsi003 | B14 | 2 | 14 | -1.60 | 0.43 | 0.88 |
| PSKH2 | hKQsi003 | B15 | 2 | 15 | -0.93 | -0.04 | -0.90 |
| GRK7 | hKQsi003 | B17 | 2 | 17 | 0.87 | -0.70 | -1.79 |
| TTBK2 | hKQsi003 | B18 | 2 | 18 | 0.38 | 0.48 | -1.14 |
| NRK | hKQsi003 | B19 | 2 | 19 | 0.92 | -0.99 | 0.18 |
| MAST4 | hKQsi003 | B20 | 2 | 20 | 0.46 | 0.68 | 0.99 |
| MAST3 | hKQsi003 | C3 | 3 | 3 | 3.86 | -0.63 | -0.22 |
| CCRK | hKQsi003 | C4 | 3 | 4 | -1.45 | 0.52 | 0.30 |
| STK23 | hKQsi003 | C5 | 3 | 5 | -0.36 | -1.34 | 0.02 |
| NME7 | hKQsi003 | C6 | 3 | 6 | 0.54 | 0.87 | 1.29 |
| PRKAG2 | hKQsi003 | C7 | 3 | 7 | -0.64 | 0.57 | 0.25 |
| EPS8L1 | hKQsi003 | C8 | 3 | 8 | 0.29 | -1.22 | -0.18 |
| STK32B | hKQsi003 | C10 | 3 | 10 | -0.84 | -0.07 | 0.38 |
| PAK6 | hKQsi003 | C11 | 3 | 11 | -0.02 | -0.21 | -0.23 |
| NJMU-R1 | hKQsi003 | C12 | 3 | 12 | 0.99 | 0.67 | -0.30 |
| LRRK1 | hKQsi003 | C13 | 3 | 13 | -0.65 | -0.49 | -0.10 |
| RPS6KL1 | hKQsi003 | C14 | 3 | 14 | 0.13 | 0.08 | -0.02 |
| NYD-SP25 | hKQsi003 | C15 | 3 | 15 | 0.37 | 1.27 | 0.16 |
| STK11IP | hKQsi003 | C17 | 3 | 17 | 0.64 | -0.87 | -1.38 |
| HIPK4 | hKQsi003 | C18 | 3 | 18 | 1.36 | -0.71 | -1.97 |
| HIPK1 | hKQsi003 | C19 | 3 | 19 | -0.43 | -0.61 | 0.92 |
| SBK1 | hKQsi003 | C20 | 3 | 20 | 0.66 | 1.18 | 0.79 |
| TNIK | hKQsi003 | D3 | 4 | 3 | -0.32 | -0.24 | -0.17 |
| DAPK2 | hKQsi003 | D4 | 4 | 4 | -0.60 | 0.81 | 0.98 |
| RPS6KC1 | hKQsi003 | D5 | 4 | 5 | 0.57 | 0.41 | 0.59 |

| | | | | | | | |
|---|---|---|---|---|---|---|---|
| PKN3 | hKQsi003 | D6 | 4 | 6 | 0.26 | 0.31 | 0.31 |
| IHPK2 | hKQsi003 | D7 | 4 | 7 | -0.44 | 0.98 | 0.42 |
| PXK | hKQsi003 | D8 | 4 | 8 | 0.15 | -1.02 | 0.03 |
| STYK1 | hKQsi003 | D10 | 4 | 10 | -0.53 | -0.44 | -0.22 |
| SPEC2 | hKQsi003 | D11 | 4 | 11 | -1.07 | -0.27 | 0.47 |
| NUCKS | hKQsi003 | D12 | 4 | 12 | -1.27 | 0.39 | 0.60 |
| PIP5K2C | hKQsi003 | D13 | 4 | 13 | -1.23 | -0.45 | -0.64 |
| RIOK1 | hKQsi003 | D14 | 4 | 14 | -1.33 | -0.62 | -0.63 |
| NYD-SP25 | hKQsi003 | D15 | 4 | 15 | 1.00 | -0.20 | -0.98 |
| HAK | hKQsi003 | D17 | 4 | 17 | 0.94 | -1.59 | -2.17 |
| PDIK1L | hKQsi003 | D18 | 4 | 18 | -1.08 | 0.39 | -0.61 |
| PRPS1L1 | hKQsi003 | D19 | 4 | 19 | -1.41 | -0.29 | -0.19 |
| discontinued | hKQsi003 | D20 | 4 | 20 | -0.67 | -0.17 | -0.31 |
| SMG1 | hKQsi003 | E3 | 5 | 3 | -0.23 | 0.79 | 0.34 |
| PRKCBP1 | hKQsi003 | E4 | 5 | 4 | -0.80 | 0.60 | 0.61 |
| AKAP8L | hKQsi003 | E5 | 5 | 5 | 0.76 | 0.28 | -0.21 |
| NRBP | hKQsi003 | E6 | 5 | 6 | 0.73 | 0.25 | -0.04 |
| NIPA | hKQsi003 | E7 | 5 | 7 | -0.64 | 0.50 | 0.18 |
| URKL1 | hKQsi003 | E8 | 5 | 8 | 1.22 | -1.68 | -0.88 |
| PI4KII | hKQsi003 | E10 | 5 | 10 | -1.01 | 0.38 | 0.57 |
| CAMK1D | hKQsi003 | E11 | 5 | 11 | -0.45 | -1.88 | -0.64 |
| CERK | hKQsi003 | E12 | 5 | 12 | 0.62 | -0.20 | -0.37 |
| NEK11 | hKQsi003 | E13 | 5 | 13 | 0.21 | -0.46 | -0.55 |
| MGC4796 | hKQsi003 | E14 | 5 | 14 | -2.56 | 0.02 | -0.41 |
| ADCK2 | hKQsi003 | E15 | 5 | 15 | 0.21 | 0.11 | -0.38 |
| IHPK3 | hKQsi003 | E17 | 5 | 17 | -1.59 | -0.73 | -0.84 |
| SNF1LK | hKQsi003 | E18 | 5 | 18 | -0.42 | -0.27 | -1.33 |
| ERK8 | hKQsi003 | E19 | 5 | 19 | -0.45 | 0.11 | -0.16 |
| discontinued | hKQsi003 | E20 | 5 | 20 | -0.15 | 0.23 | -0.08 |
| ARHGAP26 | hKQsi003 | F3 | 6 | 3 | 1.77 | -0.53 | -0.18 |
| STK22B | hKQsi003 | F4 | 6 | 4 | -0.63 | -0.34 | -0.48 |
| TPK1 | hKQsi003 | F5 | 6 | 5 | -0.50 | -0.62 | -0.05 |
| PACSIN1 | hKQsi003 | F6 | 6 | 6 | 1.07 | 0.00 | -0.04 |
| CINP | hKQsi003 | F7 | 6 | 7 | -0.72 | -0.08 | -0.76 |
| ULK4 | hKQsi003 | F8 | 6 | 8 | -0.61 | -0.32 | -0.36 |
| ALS2CR2 | hKQsi003 | F10 | 6 | 10 | 0.35 | 0.63 | 0.21 |
| ADCK1 | hKQsi003 | F11 | 6 | 11 | -0.01 | 0.37 | 0.53 |
| PINK1 | hKQsi003 | F12 | 6 | 12 | 1.83 | -0.74 | -0.79 |
| ADCK4 | hKQsi003 | F13 | 6 | 13 | 0.18 | -0.94 | -0.17 |
| STK22D | hKQsi003 | F14 | 6 | 14 | 0.40 | -0.16 | 0.18 |

| | | | | | | | |
|---|---|---|---|---|---|---|---|
| NEK9 | hKQsi003 | F15 | 6 | 15 | -1.83 | -0.57 | -1.12 |
| CIB3 | hKQsi003 | F17 | 6 | 17 | -0.77 | -0.76 | -1.22 |
| WDSUB1 | hKQsi003 | F18 | 6 | 18 | -0.77 | -0.86 | -2.15 |
| ANKK1 | hKQsi003 | F19 | 6 | 19 | -0.55 | -0.91 | -1.19 |
| discontinued | hKQsi003 | F20 | 6 | 20 | 0.35 | 0.54 | 0.37 |
| CDK11 | hKQsi003 | G3 | 7 | 3 | 1.46 | -0.55 | 0.67 |
| SGKL | hKQsi003 | G4 | 7 | 4 | -0.90 | 0.42 | 0.13 |
| HRI | hKQsi003 | G5 | 7 | 5 | 1.24 | -0.02 | -0.41 |
| SH3KBP1 | hKQsi003 | G6 | 7 | 6 | 0.45 | 0.04 | 0.02 |
| NLK | hKQsi003 | G7 | 7 | 7 | -0.83 | -0.10 | -0.36 |
| AKIP | hKQsi003 | G8 | 7 | 8 | -0.47 | 0.44 | 0.99 |
| CAMKIINALPHA | hKQsi003 | G10 | 7 | 10 | -0.13 | 0.64 | -0.11 |
| PAK7 | hKQsi003 | G11 | 7 | 11 | 0.35 | -0.94 | 0.50 |
| ALS2CR7 | hKQsi003 | G12 | 7 | 12 | -0.03 | -0.21 | 0.08 |
| PANK2 | hKQsi003 | G13 | 7 | 13 | 0.33 | -1.10 | -0.97 |
| SSTK | hKQsi003 | G14 | 7 | 14 | 0.43 | -1.71 | -0.31 |
| MLCK | hKQsi003 | G15 | 7 | 15 | -1.16 | -0.87 | -1.12 |
| PIK3AP1 | hKQsi003 | G17 | 7 | 17 | 0.44 | -0.41 | -0.53 |
| CNKSR3 | hKQsi003 | G18 | 7 | 18 | -1.76 | 0.24 | -0.45 |
| MGC40579 | hKQsi003 | G19 | 7 | 19 | 0.04 | 0.54 | 0.48 |
| discontinued | hKQsi003 | G20 | 7 | 20 | 0.32 | 0.23 | 0.58 |
| MAP3K7IP2 | hKQsi003 | H3 | 8 | 3 | 0.16 | -1.33 | -0.25 |
| PRKCN | hKQsi003 | H4 | 8 | 4 | -0.32 | -0.93 | -1.11 |
| EIF2AK4 | hKQsi003 | H5 | 8 | 5 | 0.45 | -0.44 | 0.62 |
| HUNK | hKQsi003 | H6 | 8 | 6 | 0.30 | 0.24 | -0.02 |
| UMP-CMPK | hKQsi003 | H7 | 8 | 7 | -0.33 | -0.33 | -0.62 |
| FLJ10761 | hKQsi003 | H8 | 8 | 8 | 0.10 | -0.37 | -0.63 |
| ETNK1 | hKQsi003 | H10 | 8 | 10 | -0.82 | 0.13 | -0.42 |
| CAMK1G | hKQsi003 | H11 | 8 | 11 | -0.88 | 0.29 | 0.90 |
| PRKWNK1 | hKQsi003 | H12 | 8 | 12 | -1.05 | 0.15 | 0.51 |
| LAK | hKQsi003 | H13 | 8 | 13 | 0.40 | 0.64 | -0.09 |
| CAMKK1 | hKQsi003 | H14 | 8 | 14 | -0.90 | -1.16 | -0.09 |
| LYK5 | hKQsi003 | H15 | 8 | 15 | -0.65 | -0.60 | -0.11 |
| LRRK2 | hKQsi003 | H17 | 8 | 17 | 0.49 | -1.45 | -1.35 |
| AKAP28 | hKQsi003 | H18 | 8 | 18 | -1.02 | -0.58 | -1.83 |
| MAGI-3 | hKQsi003 | H19 | 8 | 19 | 1.00 | 0.74 | -0.35 |
| LOC392265 | hKQsi003 | H20 | 8 | 20 | -1.37 | -1.63 | -0.74 |
| MAST2 | hKQsi003 | I3 | 9 | 3 | 0.97 | -0.67 | 0.40 |
| CARKL | hKQsi003 | I4 | 9 | 4 | 0.02 | 0.66 | 0.88 |
| STK36 | hKQsi003 | I5 | 9 | 5 | -0.98 | -0.47 | -1.05 |

| | | | | | | | |
|---|---|---|---|---|---|---|---|
| PIK3R4 | hKQsi003 | I6 | 9 | 6 | -1.89 | -1.37 | 0.30 |
| CRK7 | hKQsi003 | I7 | 9 | 7 | 1.17 | -0.14 | -0.15 |
| PANK4 | hKQsi003 | I8 | 9 | 8 | 0.07 | -0.78 | -0.72 |
| HSMDPKIN | hKQsi003 | I10 | 9 | 10 | 0.99 | 0.05 | -0.75 |
| CLK4 | hKQsi003 | I11 | 9 | 11 | 0.60 | 0.07 | -0.16 |
| FLJ13052 | hKQsi003 | I12 | 9 | 12 | 1.73 | 0.28 | 1.07 |
| ITPKC | hKQsi003 | I13 | 9 | 13 | -0.07 | 0.31 | -0.16 |
| KIAA1811 | hKQsi003 | I14 | 9 | 14 | -0.60 | -1.09 | 1.06 |
| PRKCDBP | hKQsi003 | I15 | 9 | 15 | 1.87 | -0.29 | -0.67 |
| CSNK1A1L | hKQsi003 | I17 | 9 | 17 | 0.12 | -1.63 | -1.79 |
| DGKH | hKQsi003 | I18 | 9 | 18 | -1.48 | 0.24 | -1.35 |
| STK32C | hKQsi003 | I19 | 9 | 19 | 0.50 | 1.96 | 0.38 |
| discontinued | hKQsi003 | I20 | 9 | 20 | -0.35 | -0.68 | -0.57 |
| MAPK8IP3 | hKQsi003 | J3 | 10 | 3 | 0.12 | 0.65 | 0.66 |
| DUSTYPK | hKQsi003 | J4 | 10 | 4 | 1.06 | -0.03 | 0.63 |
| RPS6KA6 | hKQsi003 | J5 | 10 | 5 | -0.56 | 0.33 | 0.28 |
| MINK | hKQsi003 | J6 | 10 | 6 | 1.30 | -0.81 | -0.16 |
| RP6-213H19.1 | hKQsi003 | J7 | 10 | 7 | -0.72 | -0.01 | -0.19 |
| FLJ10986 | hKQsi003 | J8 | 10 | 8 | 1.26 | -0.51 | -0.13 |
| NAGK | hKQsi003 | J10 | 10 | 10 | 0.57 | 0.03 | -0.15 |
| KIDINS220 | hKQsi003 | J11 | 10 | 11 | 0.40 | -0.17 | -0.06 |
| WNK4 | hKQsi003 | J12 | 10 | 12 | -0.69 | 0.26 | 0.05 |
| SKIP | hKQsi003 | J13 | 10 | 13 | -0.32 | -0.66 | -0.68 |
| KIAA1804 | hKQsi003 | J14 | 10 | 14 | 0.42 | 0.06 | -0.10 |
| TP53RK | hKQsi003 | J15 | 10 | 15 | -0.11 | -0.02 | -0.55 |
| AK7 | hKQsi003 | J17 | 10 | 17 | -0.38 | -1.30 | -0.62 |
| CSNK1A1P | hKQsi003 | J18 | 10 | 18 | -1.65 | -0.51 | -1.71 |
| LOC283155 | hKQsi003 | J19 | 10 | 19 | -0.15 | 0.20 | -0.71 |
| LOC407835 | hKQsi003 | J20 | 10 | 20 | -0.28 | -0.36 | -0.39 |
| PASK | hKQsi003 | K3 | 11 | 3 | 0.47 | 0.38 | 0.19 |
| PRKD2 | hKQsi003 | K4 | 11 | 4 | -0.23 | 0.02 | -0.63 |
| STK39 | hKQsi003 | K5 | 11 | 5 | -0.79 | 1.18 | 0.71 |
| AK3L1 | hKQsi003 | K6 | 11 | 6 | 0.77 | -1.35 | -0.58 |
| ZAK | hKQsi003 | K7 | 11 | 7 | -0.11 | 0.43 | 0.08 |
| PI4K2B | hKQsi003 | K8 | 11 | 8 | -1.09 | -0.81 | -0.72 |
| BMP2K | hKQsi003 | K10 | 11 | 10 | -0.52 | 0.73 | 0.40 |
| KIAA1361 | hKQsi003 | K11 | 11 | 11 | 0.19 | 0.01 | 0.12 |
| PRKWNK3 | hKQsi003 | K12 | 11 | 12 | -0.10 | 0.11 | -0.26 |
| GKAP1 | hKQsi003 | K13 | 11 | 13 | 0.51 | -0.28 | -0.83 |
| TTBK1 | hKQsi003 | K14 | 11 | 14 | 1.35 | 0.11 | 0.70 |

| | | | | | | | |
|---|---|---|---|---|---|---|---|
| LMTK3 | hKQsi003 | K15 | 11 | 15 | -0.24 | -0.84 | -1.42 |
| UHMK1 | hKQsi003 | K17 | 11 | 17 | -0.23 | 0.09 | -0.66 |
| MGC45428 | hKQsi003 | K18 | 11 | 18 | -2.95 | 0.58 | 0.16 |
| KSR2 | hKQsi003 | K19 | 11 | 19 | 0.72 | 1.23 | -0.14 |
| MAST4 | hKQsi003 | L3 | 12 | 3 | -0.22 | 0.36 | 1.28 |
| DKFZP434C131 | hKQsi003 | L4 | 12 | 4 | -0.98 | -0.81 | -0.27 |
| GIT1 | hKQsi003 | L5 | 12 | 5 | -0.23 | 0.48 | 0.14 |
| TNNI3K | hKQsi003 | L6 | 12 | 6 | 0.78 | 0.28 | -0.01 |
| PANK1 | hKQsi003 | L7 | 12 | 7 | -0.58 | 0.95 | 1.67 |
| RIOK2 | hKQsi003 | L10 | 12 | 10 | 1.13 | -0.15 | 0.15 |
| KIAA1446 | hKQsi003 | L11 | 12 | 11 | 0.70 | -0.33 | 0.20 |
| PRKWNK2 | hKQsi003 | L12 | 12 | 12 | 0.39 | -0.04 | 0.04 |
| TRAF3IP3 | hKQsi003 | L13 | 12 | 13 | -0.09 | -0.33 | -0.66 |
| FLJ14800 | hKQsi003 | L14 | 12 | 14 | 0.56 | -0.20 | 0.02 |
| IRAK1BP1 | hKQsi003 | L17 | 12 | 17 | -0.45 | -0.41 | -1.02 |
| FUK | hKQsi003 | L18 | 12 | 18 | -3.06 | -0.96 | 5.27 |
| NEK8 | hKQsi003 | L19 | 12 | 19 | -1.46 | 0.85 | -0.11 |
| SIK2 | hKQsi003 | M3 | 13 | 3 | 0.03 | 0.56 | 0.92 |
| IBTK | hKQsi003 | M4 | 13 | 4 | -1.78 | 1.39 | 0.82 |
| HIPK2 | hKQsi003 | M5 | 13 | 5 | -1.49 | 0.14 | 0.86 |
| IRAK4 | hKQsi003 | M6 | 13 | 6 | 0.92 | 0.58 | 1.09 |
| PRKAG3 | hKQsi003 | M7 | 13 | 7 | -1.48 | 0.48 | 0.49 |
| TOPK | hKQsi003 | M10 | 13 | 10 | -0.79 | -0.07 | 0.43 |
| MARK4 | hKQsi003 | M11 | 13 | 11 | -0.20 | -0.72 | 0.20 |
| STK33 | hKQsi003 | M12 | 13 | 12 | -0.84 | -1.86 | -0.53 |
| STK22C | hKQsi003 | M13 | 13 | 13 | -0.45 | 0.73 | 0.48 |
| MASTL | hKQsi003 | M14 | 13 | 14 | -0.83 | 0.42 | 0.68 |
| PNCK | hKQsi003 | M17 | 13 | 17 | 0.18 | -0.55 | -1.00 |
| PIP5K3 | hKQsi003 | M18 | 13 | 18 | -2.41 | -0.04 | 0.54 |
| discontinued | hKQsi003 | M19 | 13 | 19 | -0.79 | 2.22 | 2.32 |
| PIP5K1C | hKQsi003 | N3 | 14 | 3 | -0.58 | 1.03 | 2.21 |
| AK5 | hKQsi003 | N4 | 14 | 4 | -1.00 | 0.42 | 0.32 |
| TBK1 | hKQsi003 | N5 | 14 | 5 | 0.69 | 0.37 | 0.36 |
| VRK3 | hKQsi003 | N6 | 14 | 6 | 0.19 | 0.67 | 0.55 |
| CSNK1G1 | hKQsi003 | N7 | 14 | 7 | -0.79 | 0.17 | -0.02 |
| LOC55971 | hKQsi003 | N10 | 14 | 10 | -0.25 | 0.76 | 0.92 |
| TSKS | hKQsi003 | N11 | 14 | 11 | 0.34 | 0.78 | 0.91 |
| MAPKAP1 | hKQsi003 | N12 | 14 | 12 | 0.00 | -0.74 | -0.48 |
| SNARK | hKQsi003 | N13 | 14 | 13 | -0.72 | -0.37 | -0.67 |
| PDXK | hKQsi003 | N14 | 14 | 14 | 0.35 | 1.11 | -0.13 |

| | | | | | | | |
|---|---|---|---|---|---|---|---|
| NEK7 | hKQsi003 | N17 | 14 | 17 | -0.85 | -0.32 | -1.13 |
| STK32A | hKQsi003 | N18 | 14 | 18 | -0.10 | 0.21 | -0.10 |
| NEK5 | hKQsi003 | N19 | 14 | 19 | -0.03 | 0.47 | 0.42 |
| P101-PI3K | hKQsi003 | O3 | 15 | 3 | 0.62 | 1.57 | 0.98 |
| HSPB8 | hKQsi003 | O4 | 15 | 4 | 0.70 | -2.02 | -0.96 |
| PACSIN3 | hKQsi003 | O5 | 15 | 5 | 0.43 | 0.83 | 1.00 |
| CDKL3 | hKQsi003 | O6 | 15 | 6 | -0.98 | 0.16 | -0.14 |
| ANKRD3 | hKQsi003 | O7 | 15 | 7 | -0.04 | -0.08 | 0.35 |
| STK31 | hKQsi003 | O10 | 15 | 10 | -0.78 | 0.96 | 1.65 |
| RBKS | hKQsi003 | O11 | 15 | 11 | 1.24 | 0.96 | 0.31 |
| PANK3 | hKQsi003 | O12 | 15 | 12 | -0.37 | -0.95 | -0.23 |
| SHARPIN | hKQsi003 | O13 | 15 | 13 | 0.57 | -0.75 | -0.25 |
| MYLK2 | hKQsi003 | O14 | 15 | 14 | -0.43 | 0.83 | 2.07 |
| STK35 | hKQsi003 | O17 | 15 | 17 | 0.04 | 1.75 | -0.02 |
| ADCK5 | hKQsi003 | O18 | 15 | 18 | -2.50 | -1.23 | -0.15 |
| CDKL4 | hKQsi003 | O19 | 15 | 19 | -0.48 | 1.04 | 1.70 |
