# Supplementary figures and images for: High-throughput kinome-RNAi screen identifies protein kinase R activator (PACT) as a novel genetic modifier of CUG foci integrity in myotonic dystrophy type 1 (DM1)
Source: PLoS One. 2021 Sep 14;16(9):e0256276. doi: 10.1371/journal.pone.0256276 (PMC8439471; doi:10.1371/journal.pone.0256276)

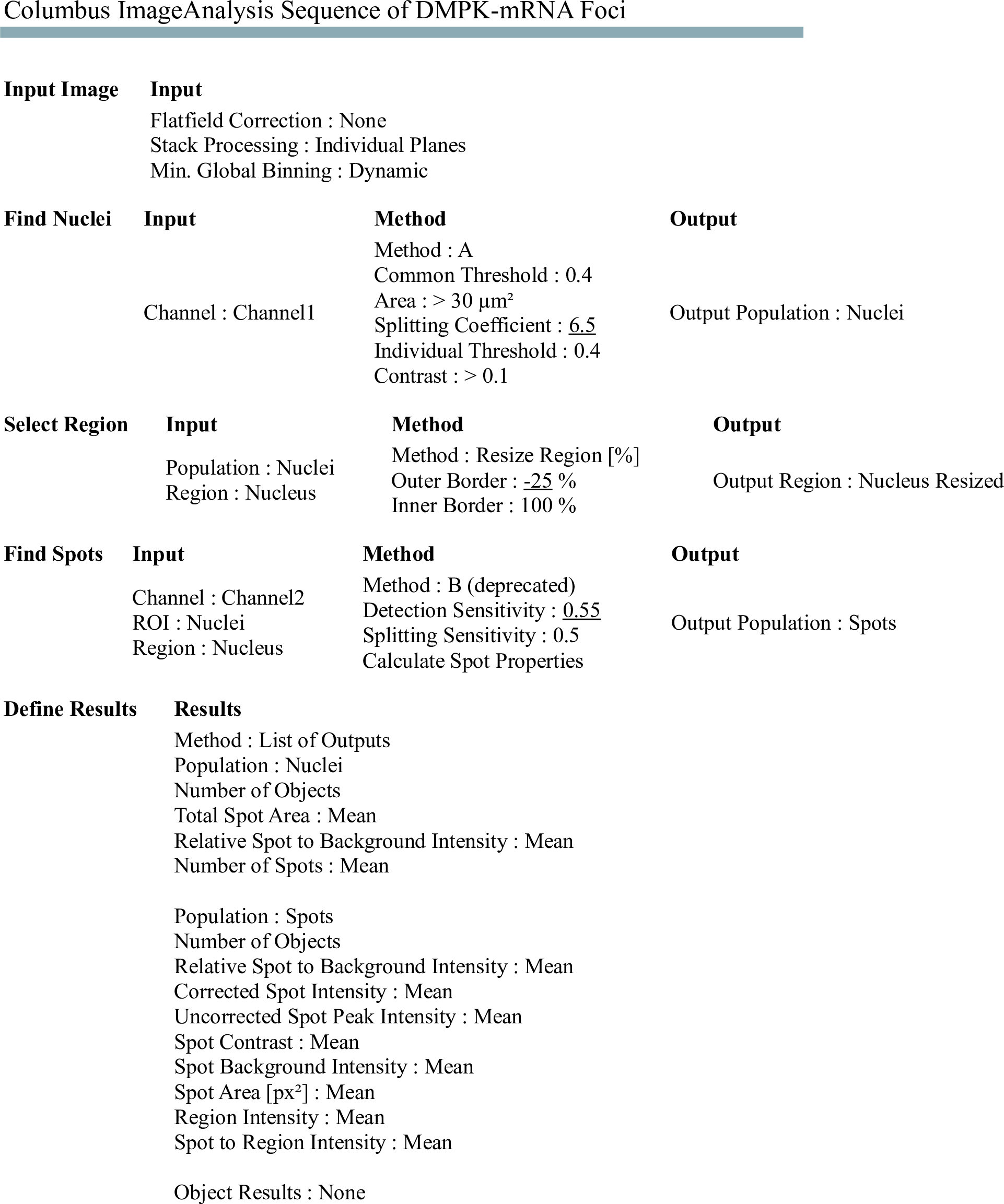

Supplement: S2 Table — (TIF) [file pone.0256276.s002.tif]

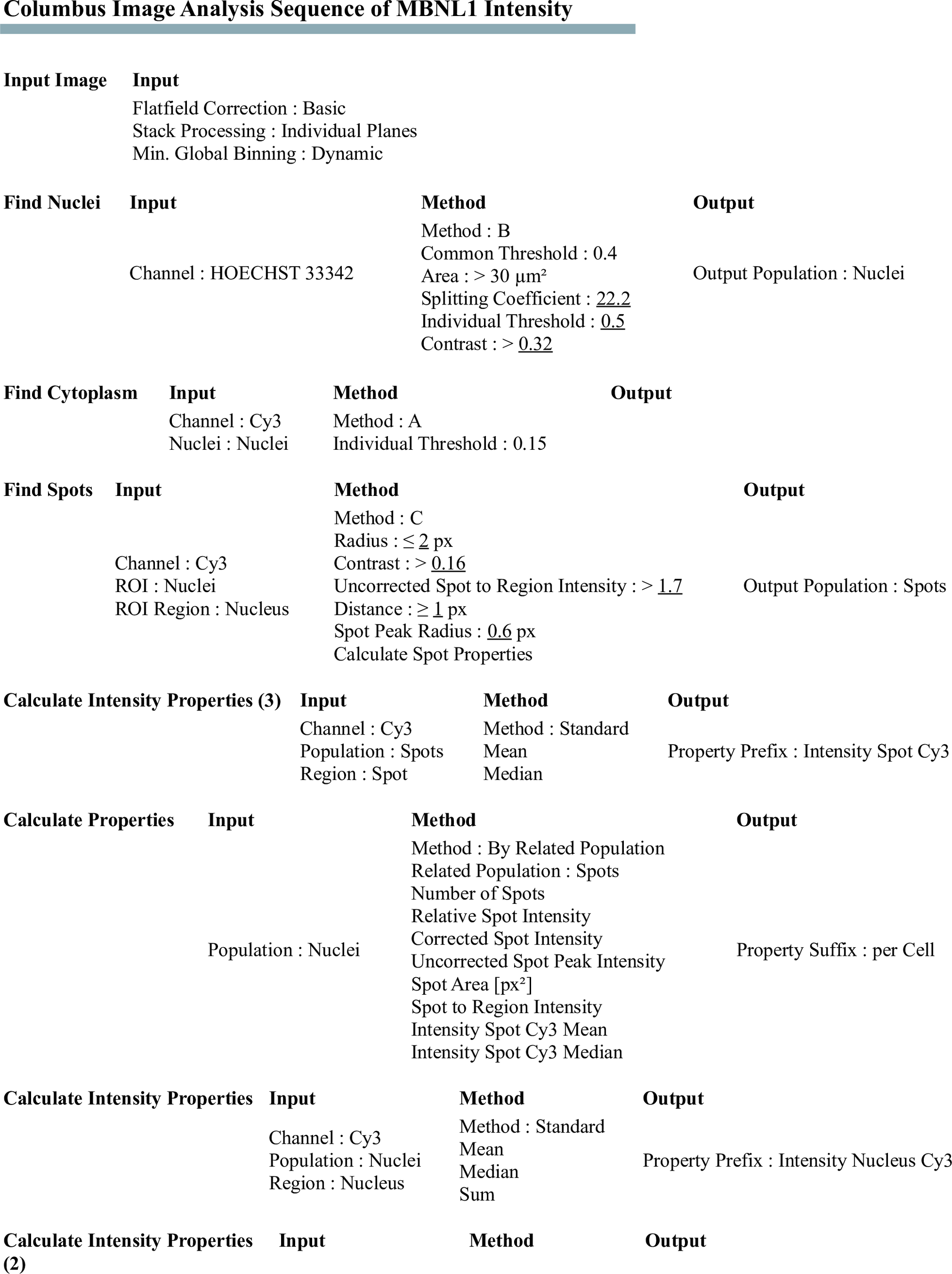

Supplement: S3 Table — (TIF) [file pone.0256276.s003.tif]

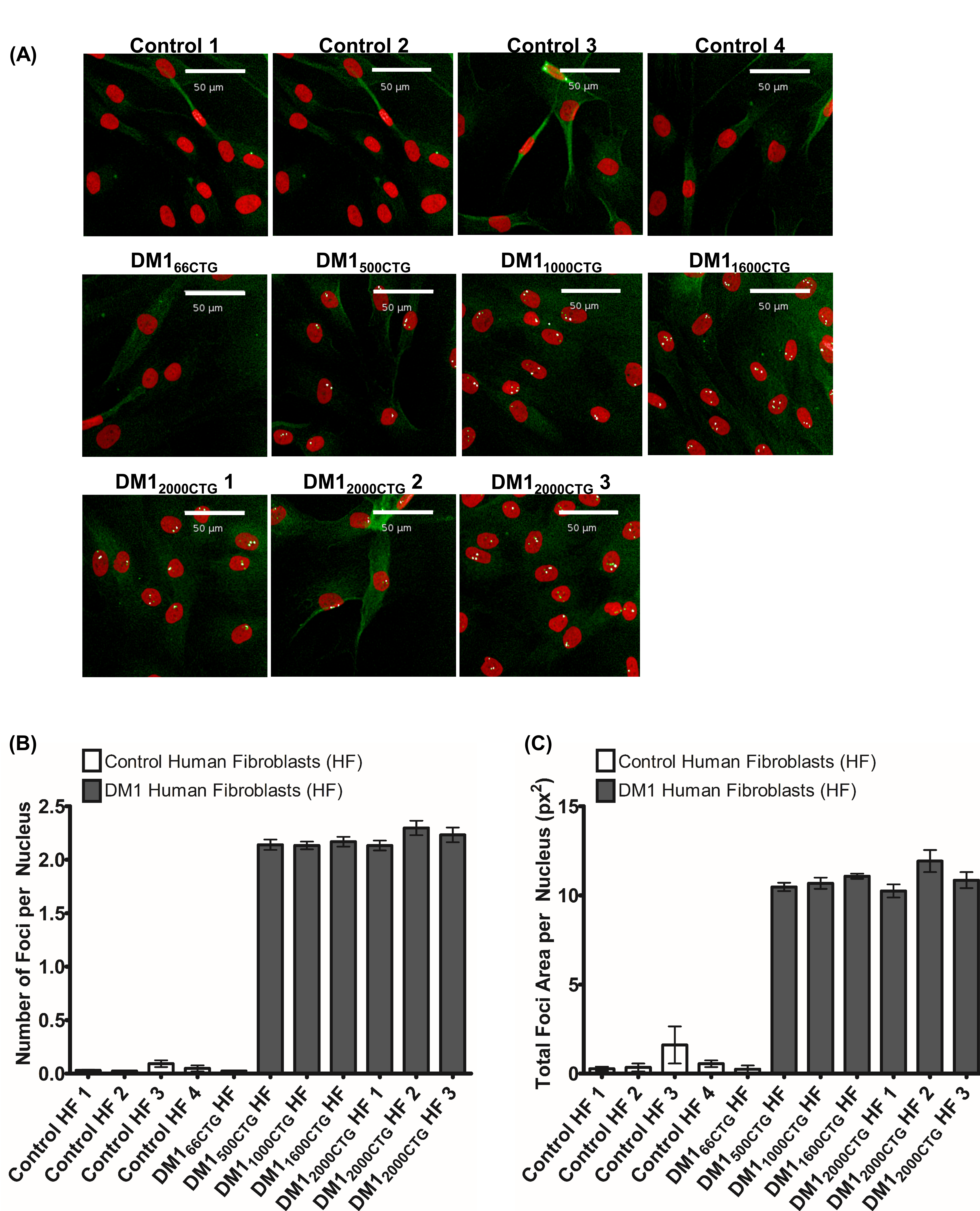

Supplement: S1 Fig — (A) Representative images of nuclear foci (blue–Hoechst; green–CUG foci) after RNA FISH of CUG foci in control and patient skin fibroblasts. Quantification of (B) number of foci per nucleus and (C) total foci area per nucleus using means data from five replicate wells (n = 5; two-way ANOVA; errors represent SD). (TIF) [file pone.0256276.s004.tif]

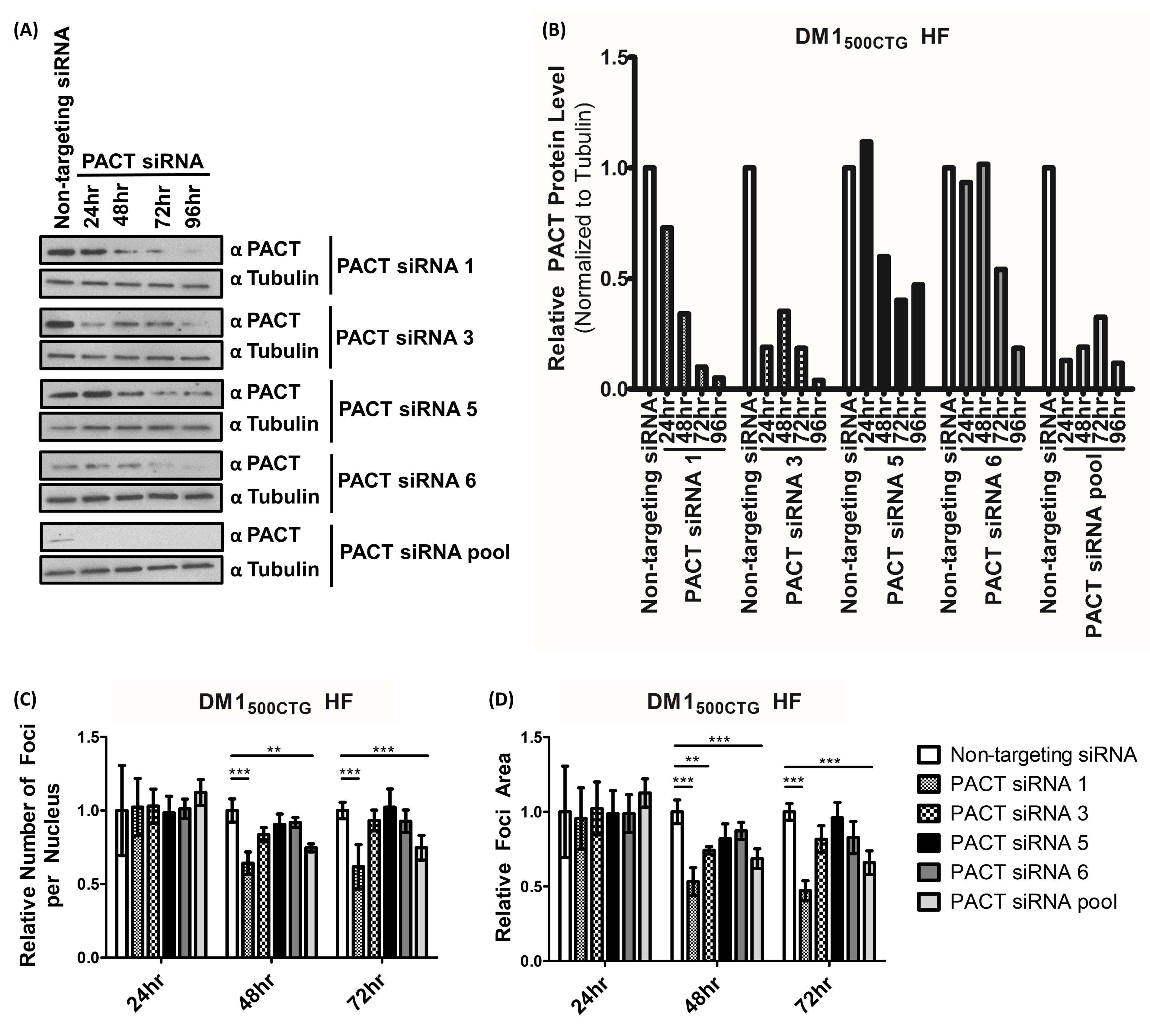

Supplement: S2 Fig — (A) Western blot analysis of PACT knockdown using individual and pooled PACT siRNA. (B) Quantification of PACT knockdown in (A) was done by densitometric analysis using Image J. (C-D) Foci integrity upon PACT knockdown. Cells were probed with Alexa-555 (CAG)10 probes to detect and image foci. Quantification of (C) number of foci per nucleus and (D) total foci area per nucleus was done using the Columbus software and mean data from five replicate wells is shown (n = 5; two-way ANOVA; error bars represent SD). *P < 0.05, **P < 0.01, ***P< 0.001. (TIF) [file pone.0256276.s005.tif]

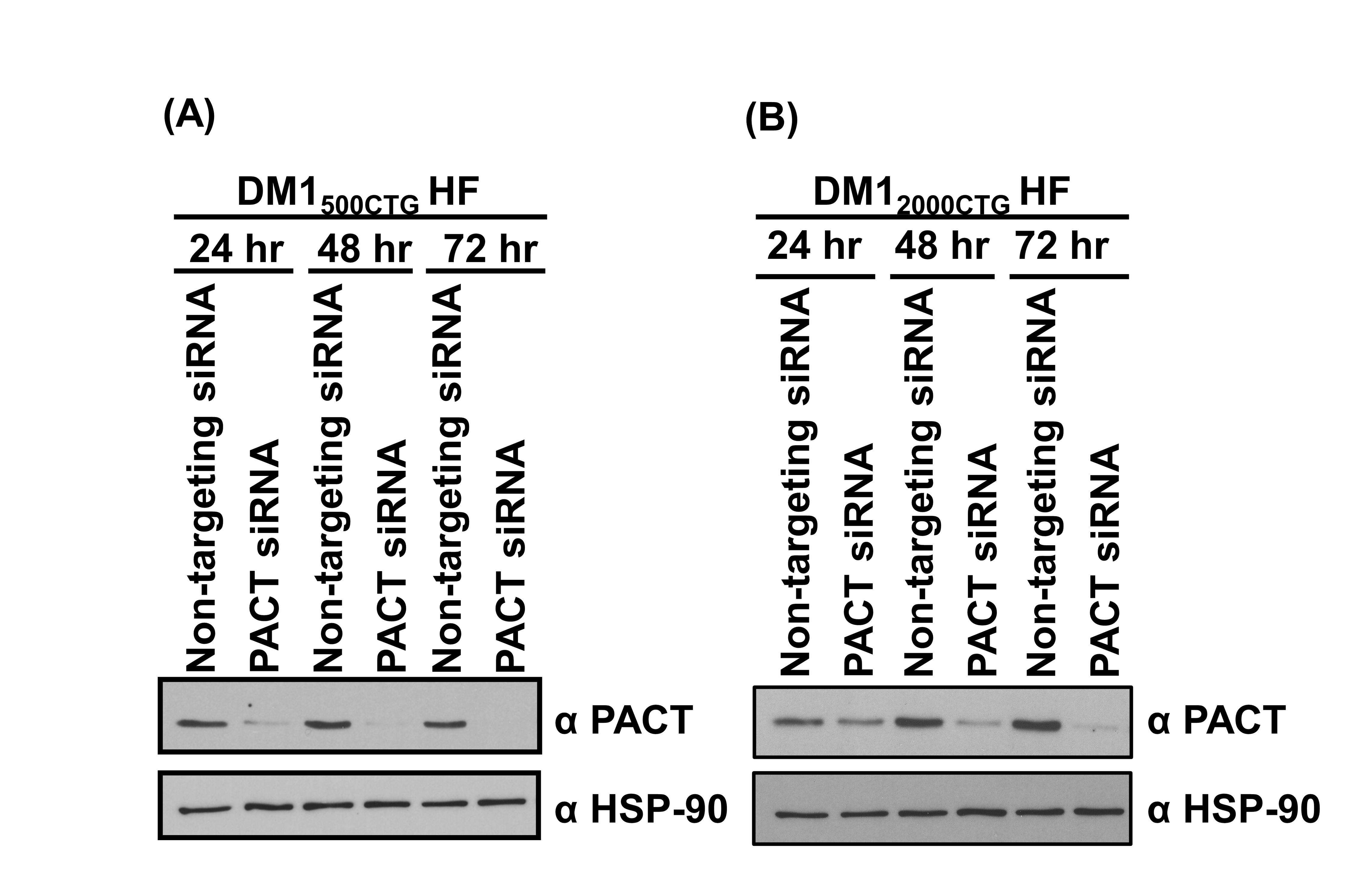

Supplement: S3 Fig — Western blot analysis of PACT protein levels upon knockdown using PACT siRNA in (A) DM1500 CTG and (B) DM12000 CTG patient fibroblasts, respectively. (TIF) [file pone.0256276.s006.tif]

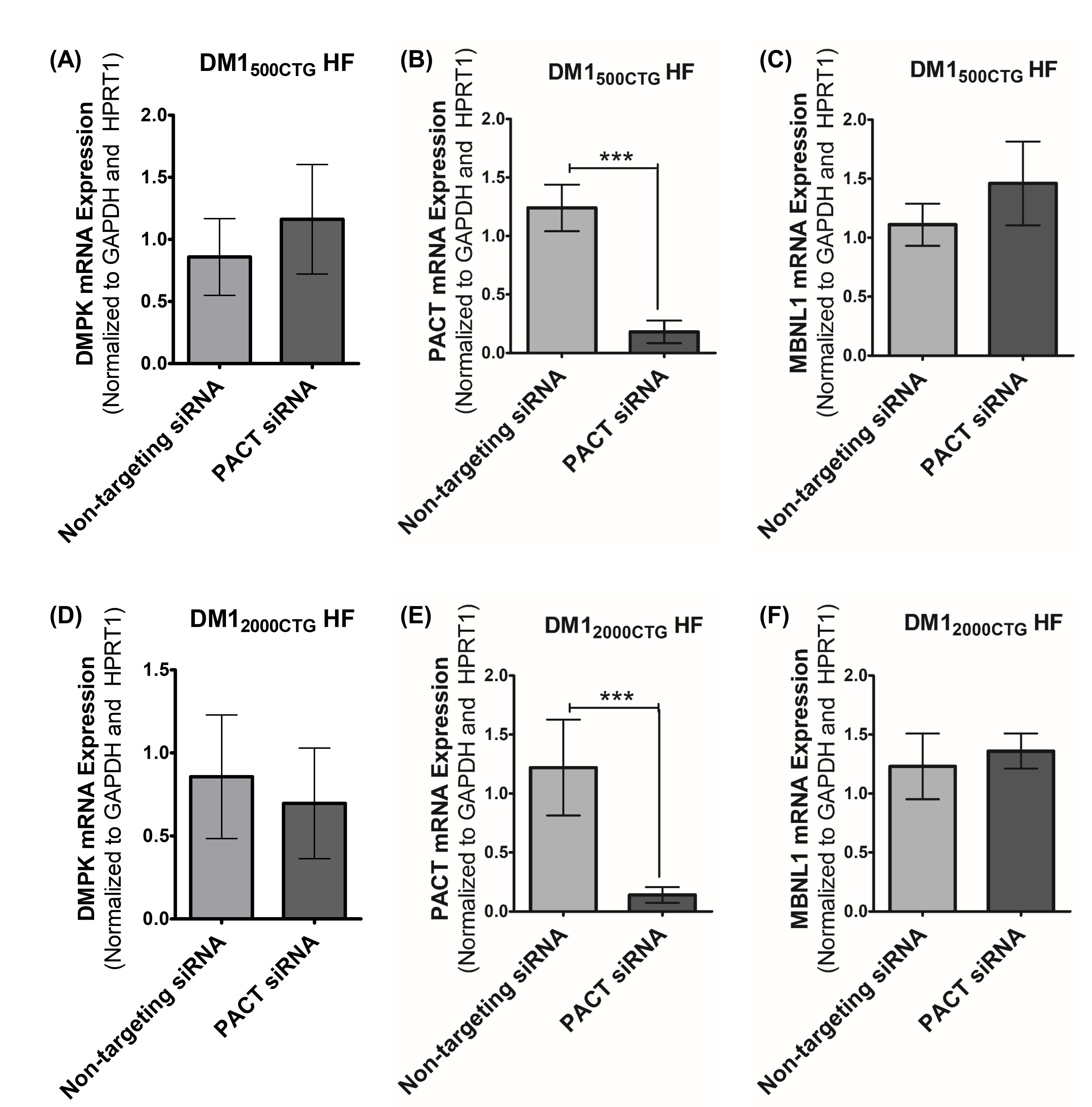

Supplement: S4 Fig — RT-qPCR analysis of (A and D) DMPK, (B and E) PACT, and (C and F) MBNL1 transcript levels in DM1500CTG and DM12000CTG HF, respectively, after a 72hr treatment with PACT siRNA; target genes were normalized to GAPDH and HPRT1 as reference genes. Samples were run in duplicates for each gene with replicates of n = 5. (TIF) [file pone.0256276.s007.tif]

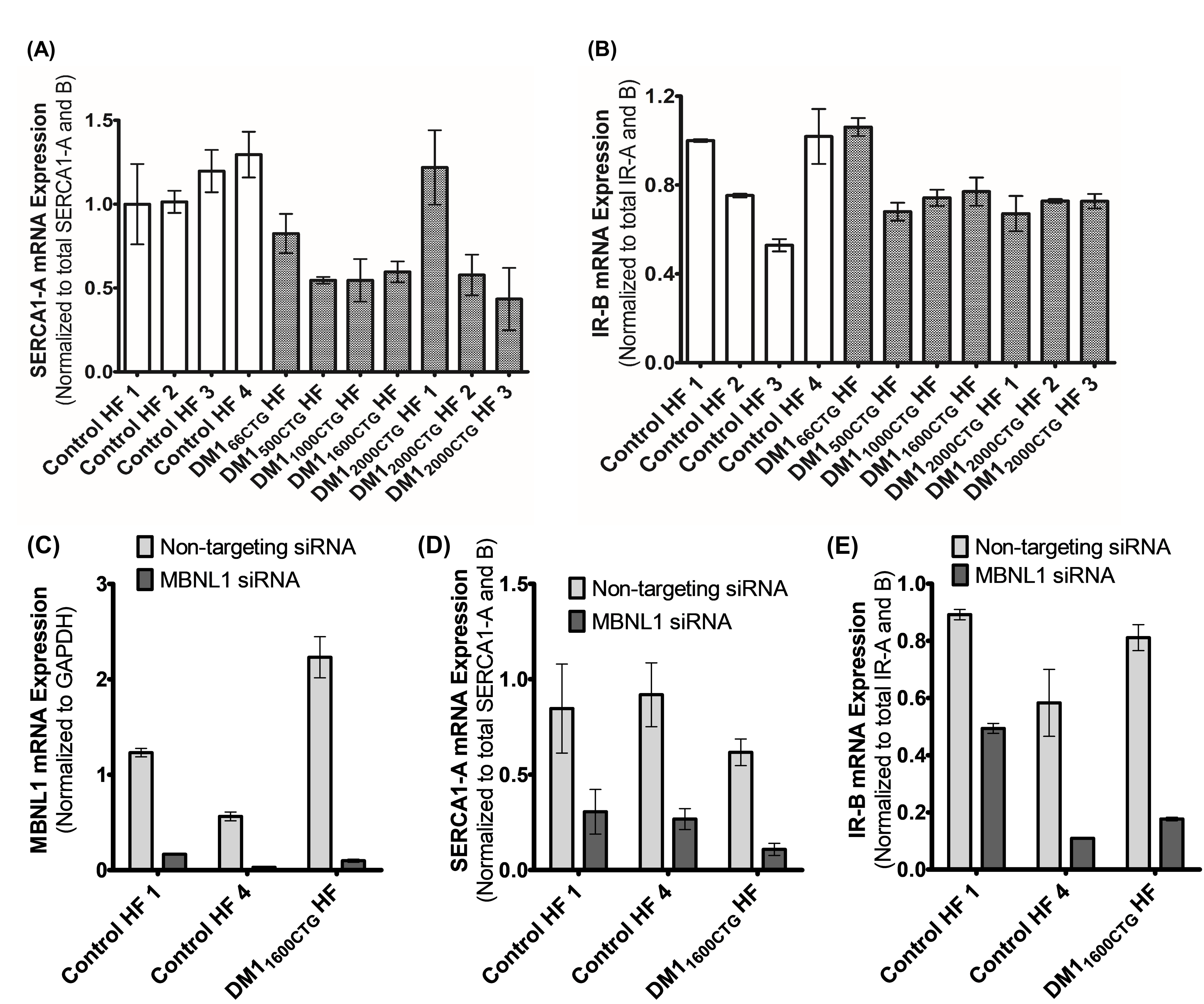

Supplement: S5 Fig — (A) SERCA1 and (B) IR splicing patterns in Control HF versus DM1 HF using RT-qPCR. Error bars are representative of duplicate qPCR runs, SEM. (A) SERCA1-A levels were measured using an exon 22-specific primer and quantified against total SERCA1-A and B as the reference gene. (B) IR-B mRNA levels were measured using an exon 11-specific primer and quantified against total IR-A and B as the reference gene. MBNL1 knockdown was used to validate sensitivity of the assay in detecting changes in splicing upon treatment. (C) MBNL1 with reference to GAPDH, (D) SERCA1-A with reference to SERCA1-AB, and (E) IR-B with reference to IR-AB with measured in duplicate qPCR runs. (TIF) [file pone.0256276.s008.tif]

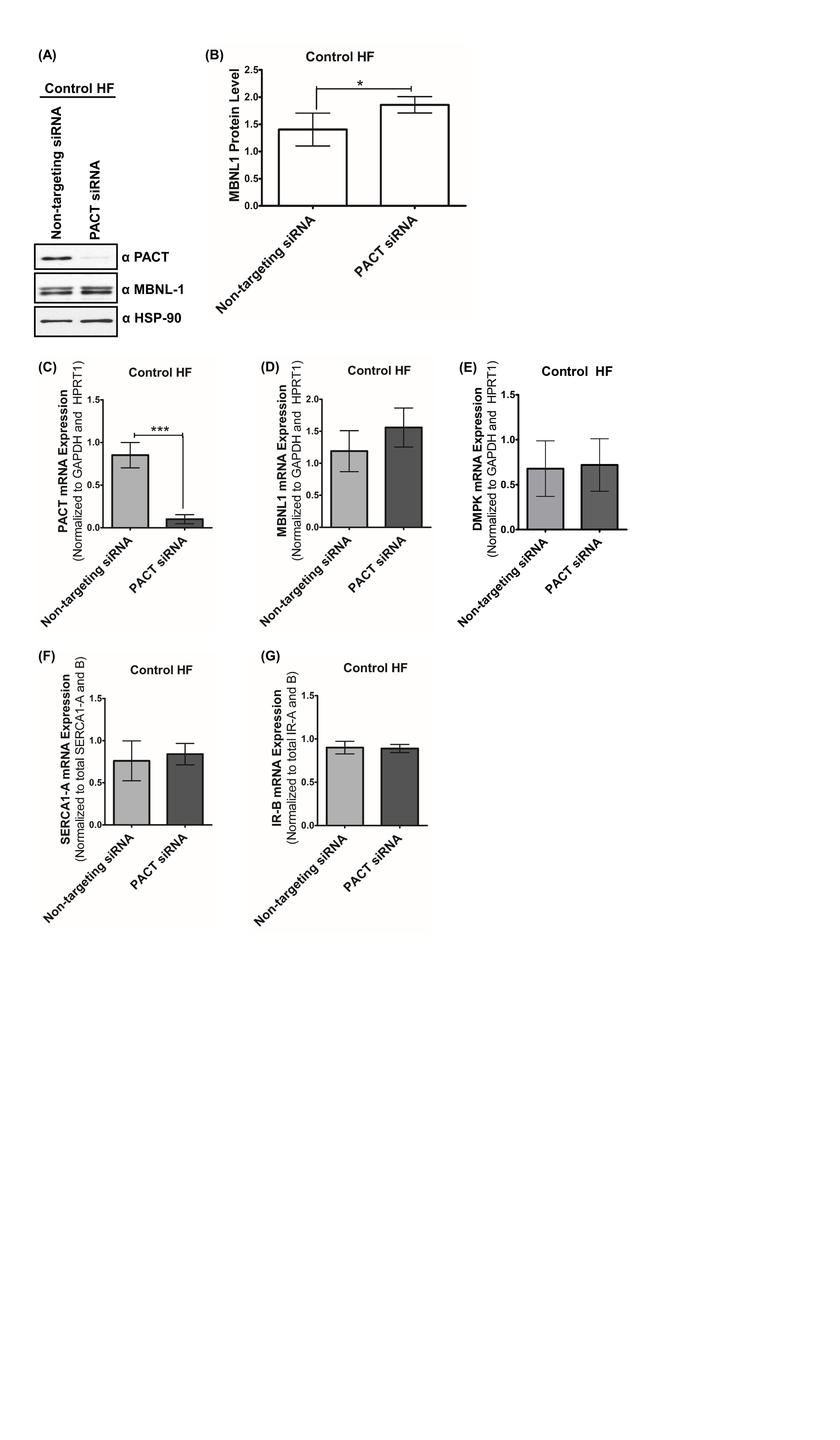

Supplement: S6 Fig — (A) Western blot analysis of MBNL1 protein levels upon PACT knockdown and (B) the corresponding quantification using Image J software (n = 3; unpaired t-test; error bars represent SD). MBNL1 protein level was normalized to HSP90 loading control and the normalized values were averaged across trials. (C) PACT mRNA, (D) MBNL1 mRNA, and (E) DMPK mRNA, all three with reference to GAPDH and HPRT1; (F) SERCA1-A with reference to SERCA1-AB; and (G) IR-B with reference to IR-AB were measured in duplicate qPCR runs per sample. The non-targeting siRNA treated sample in one trial was set as the “control” sample and all other sample, non-targeting siRNA and PACT siRNA treated sample from all other trials, were quantified relative to this “control” (n = 5; unpaired t-test; error bars represent SD). (TIF) [file pone.0256276.s009.tif]

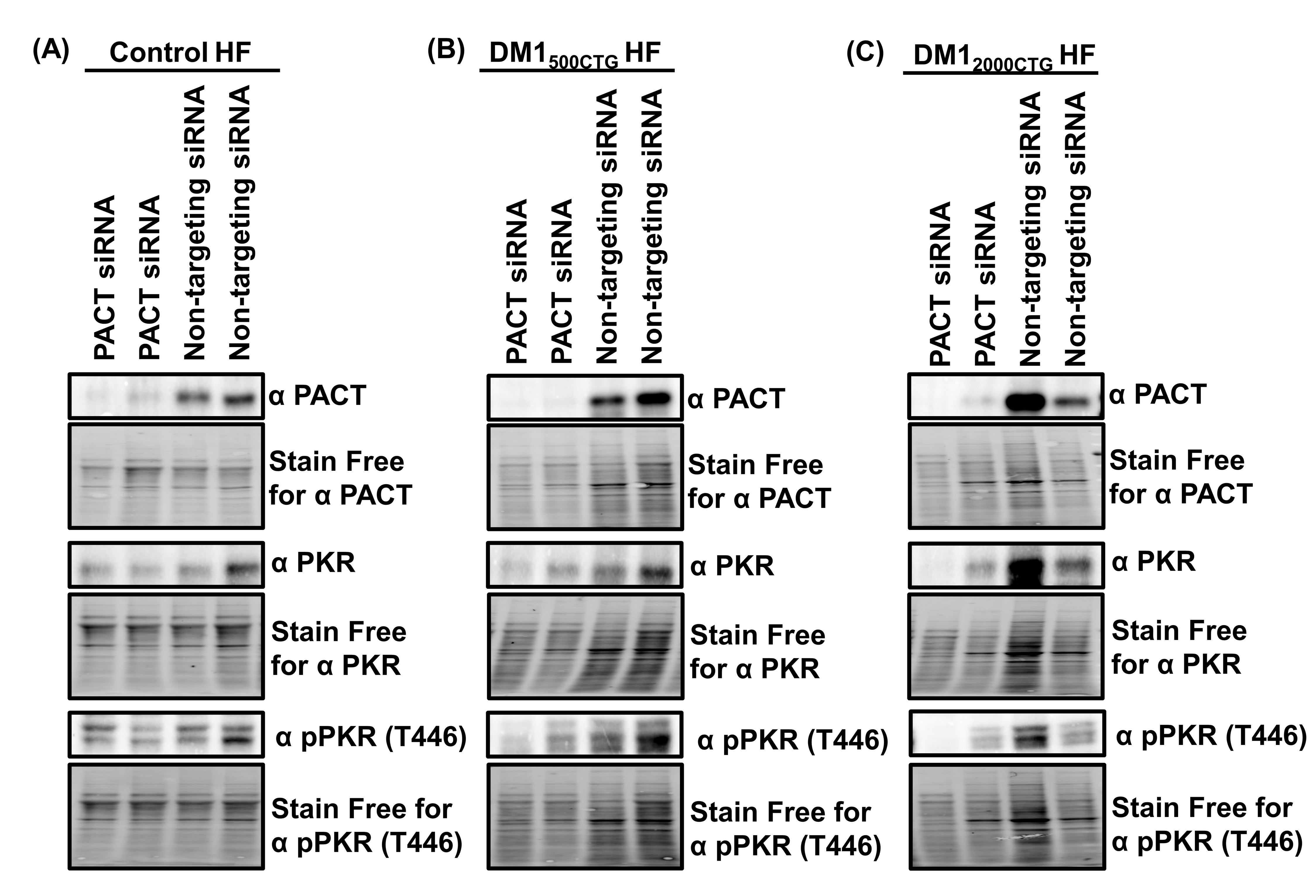

Supplement: S7 Fig — PACT, PKR and phospho-PKR (Tyrosine 446) levels following PACT siRNA in HF (A), DM1500 CTG (B) and DM12000 CTG (C). (TIF) [file pone.0256276.s010.tif]

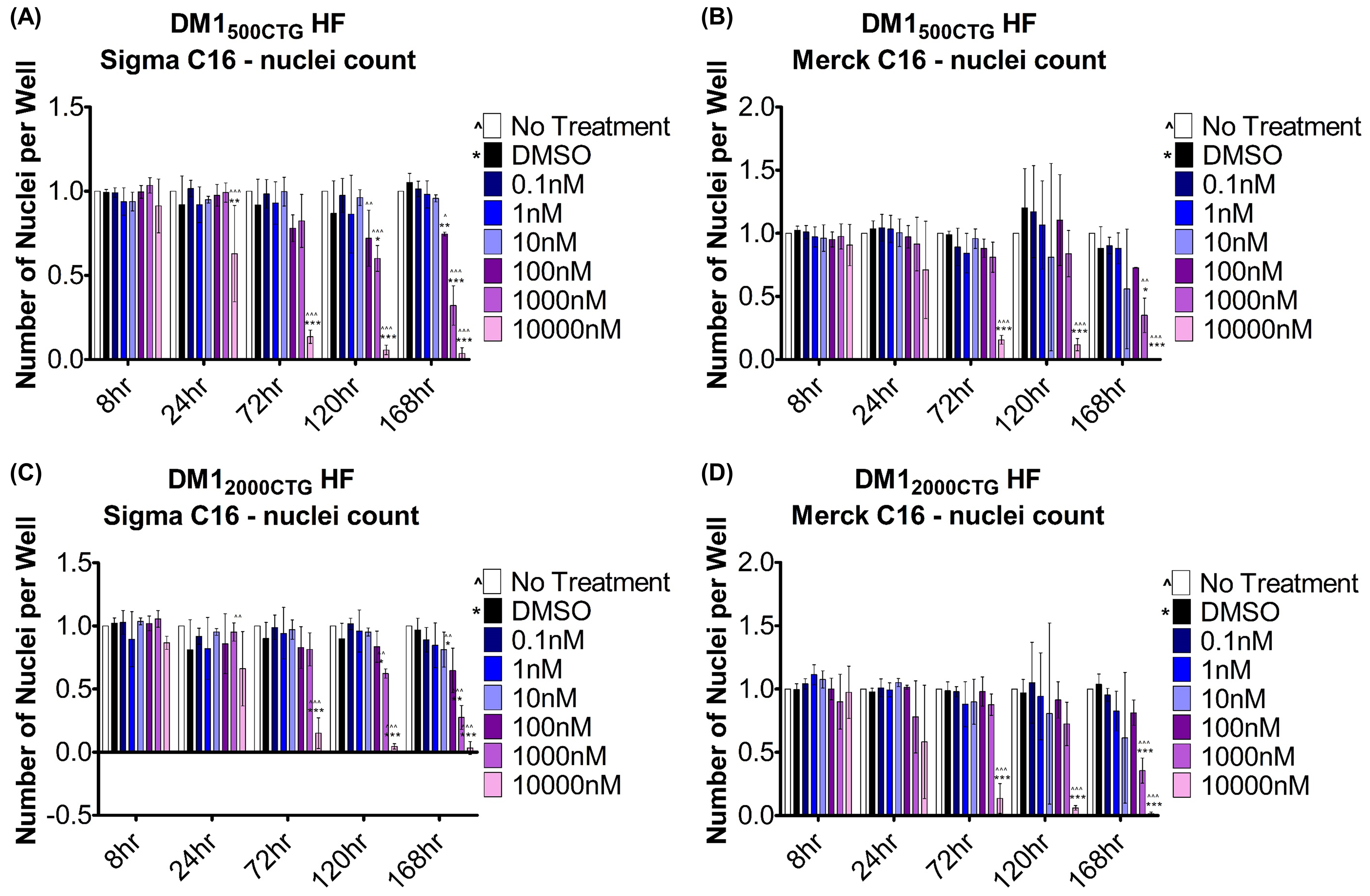

Supplement: S8 Fig — Small molecule PKR inhibitor C16 was used to treat (A) DM1-500500CTG HF or (B) DM12000cTG HF at doses ranging from 0.1nM-10,000nM for 8hrs up to 168hrs (7 days) following which the nucleus was stained with Hoechst. Number of nuclei per well was quantified using Columbus software (n = 3; two-way ANOVA; error bars represent SD). (TIF) [file pone.0256276.s011.tif]

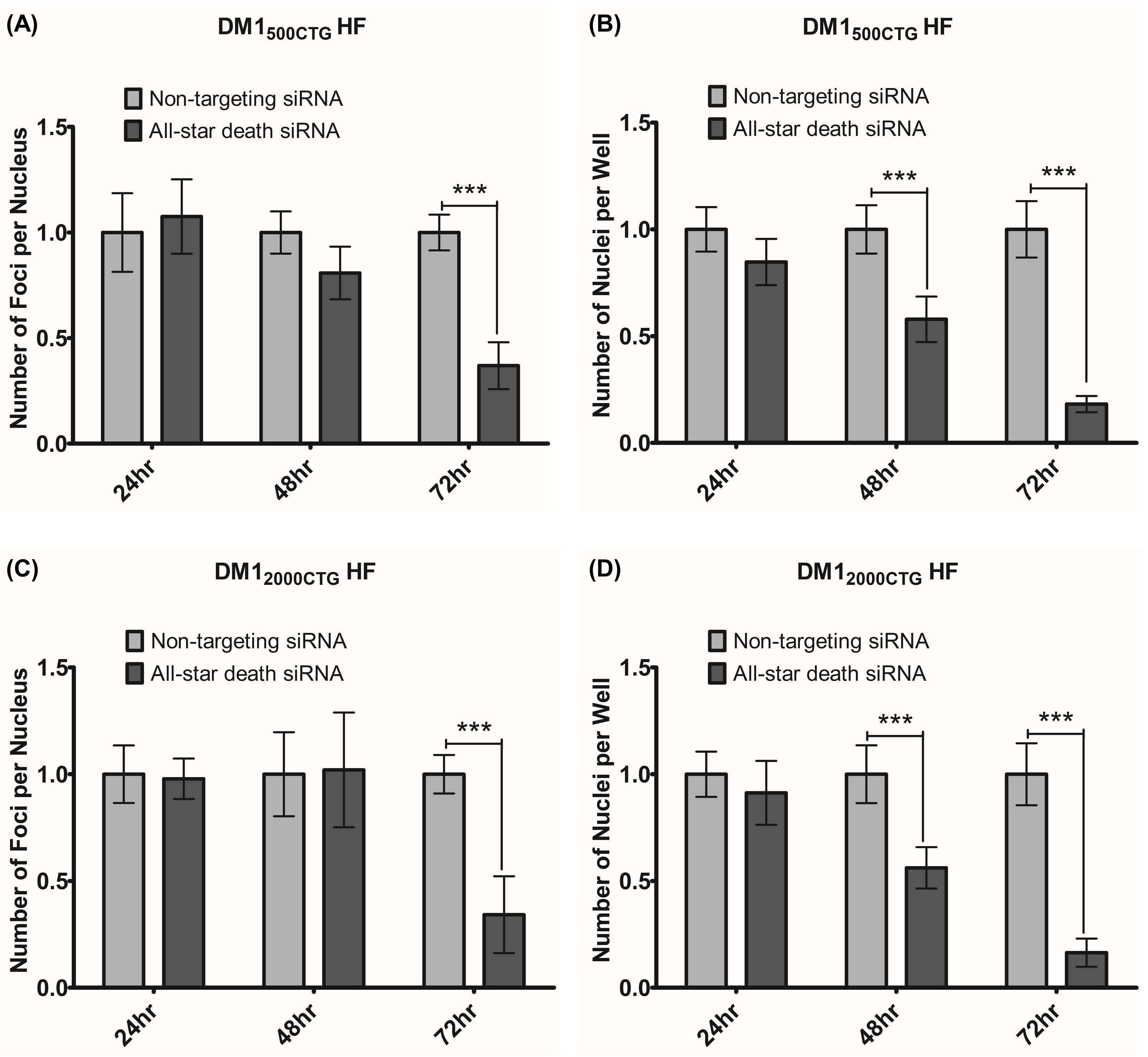

Supplement: S9 Fig — Effect of AllStars death siRNA on number of cells (A, C) and number of foci per nucleus (B, D) in DM1500 CTG and DM12000 CTG, respectively. Cells were stained with Hoechst to visualize the nuclei and probed with Alexa-555 (CAG)10 probes to detect foci. Quantification was done using the Columbus software (n = 4; two-way ANOVA; error bars represent SD). For statistical analysis, each trial consisted of mean data from 3–9 replicate wells per sample with corresponding SD. The data was converted to fold change relative to non-targeting siRNA to accommodate plate-to-plate variability and the SD was adjusted to the same ratio. The fold change and SD data were averaged across all trials. (TIF) [file pone.0256276.s012.tif]

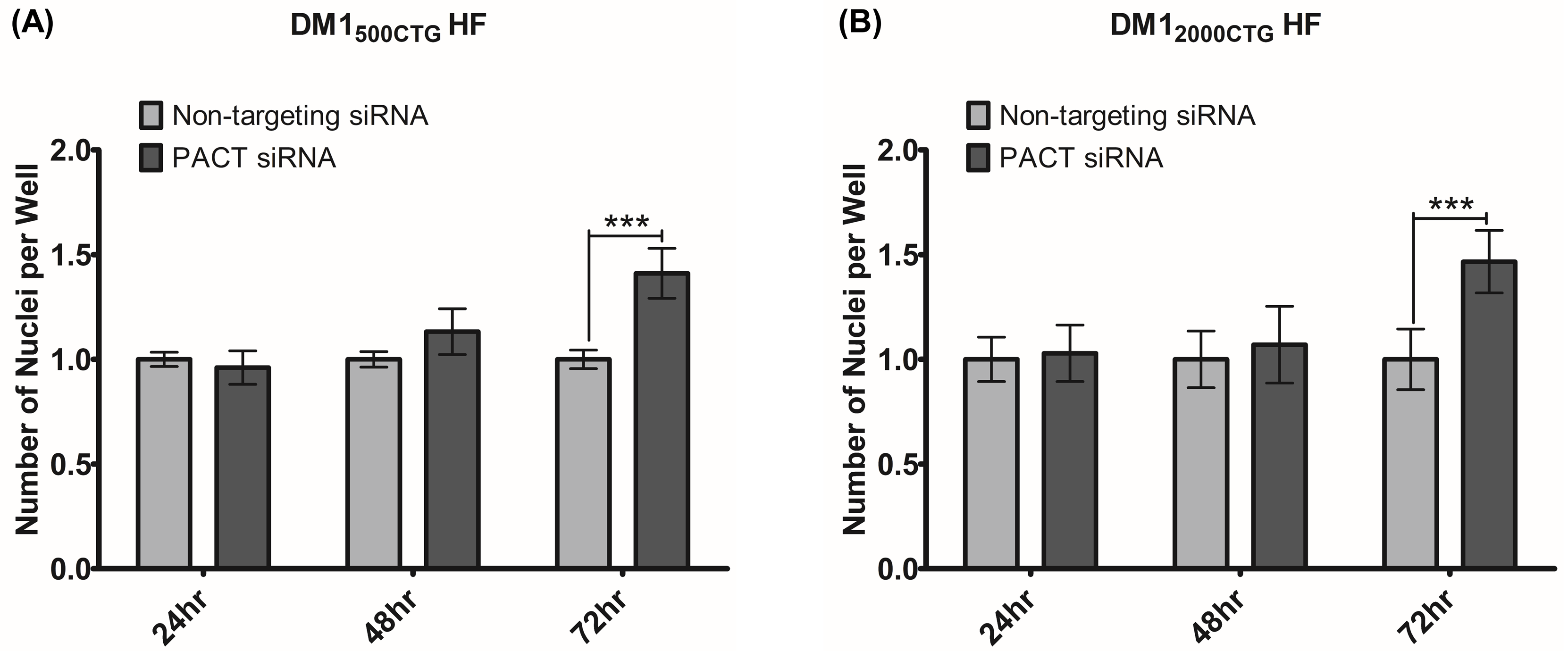

Supplement: S10 Fig — Effect of PACT siRNA treatment on number of cells in (A) DM1500 CTG and (B) DM12000 CTG HF. Cells were stained with Hoechst to visualize the nuclei. Quantification was done using the Columbus software (n = 4; two-way ANOVA; error bars represent SD). For statistical analysis, each trial consisted of mean data from 3–9 replicate wells per sample with corresponding SD. The data was converted to fold change relative to non-targeting siRNA to accommodate plate-to-plate variability and the SD was adjusted to the same ratio. The fold change and SD data were averaged across all trials. (TIF) [file pone.0256276.s013.tif]
