# Supplementary material for: High-throughput kinome-RNAi screen identifies protein kinase R activator (PACT) as a novel genetic modifier of CUG foci integrity in myotonic dystrophy type 1 (DM1)
Source: PLoS One. 2021 Sep 14;16(9):e0256276. doi: 10.1371/journal.pone.0256276 (PMC8439471; doi:10.1371/journal.pone.0256276)

Figure 3, Supplementary Figure 6A-B
Trial 1:

anti-PACT (film, chemiluminescence)

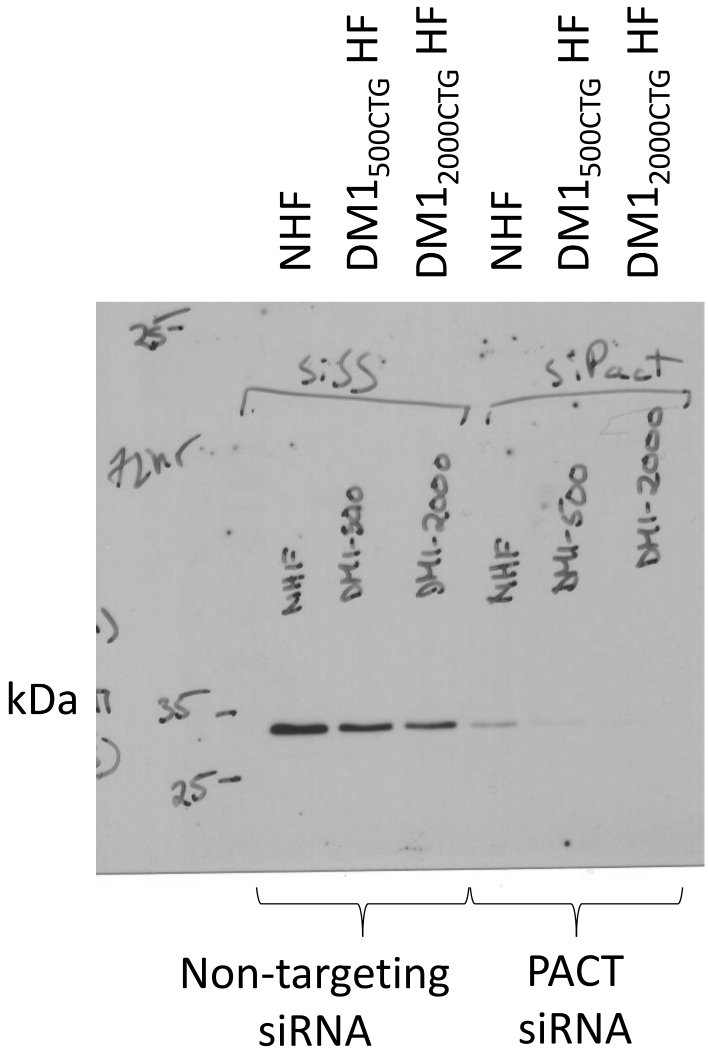

Figure 3, Supplementary Figure 6A-B
Trial 1:
anti-MBNL1 (film, chemiluminescence)

(exposure A used for quantification; exposure B used to show markings for the ladder size reference)

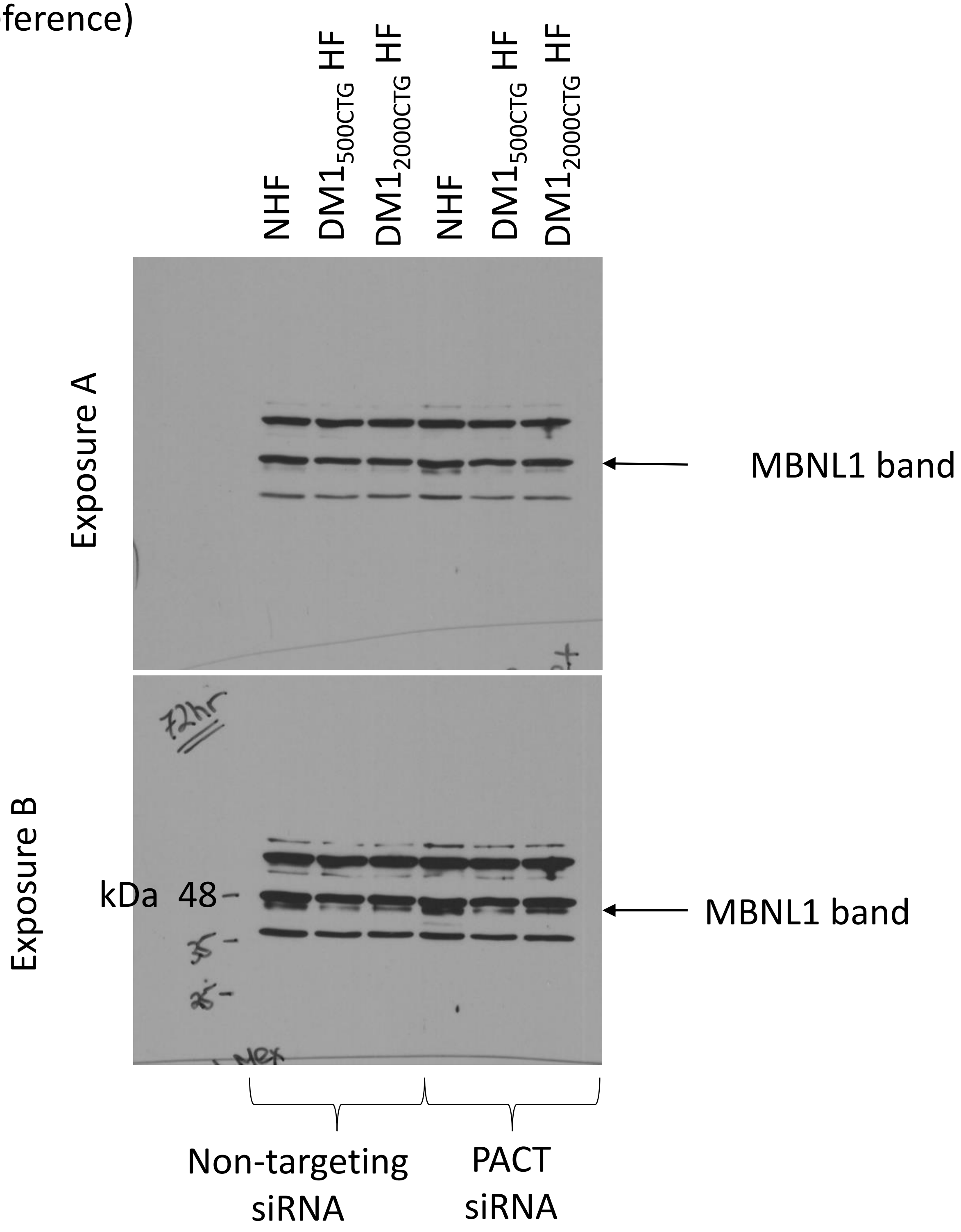

Figure 3, Supplementary Figure 6A-B
Trial 1:

anti-HSP90 (film, chemiluminescence)

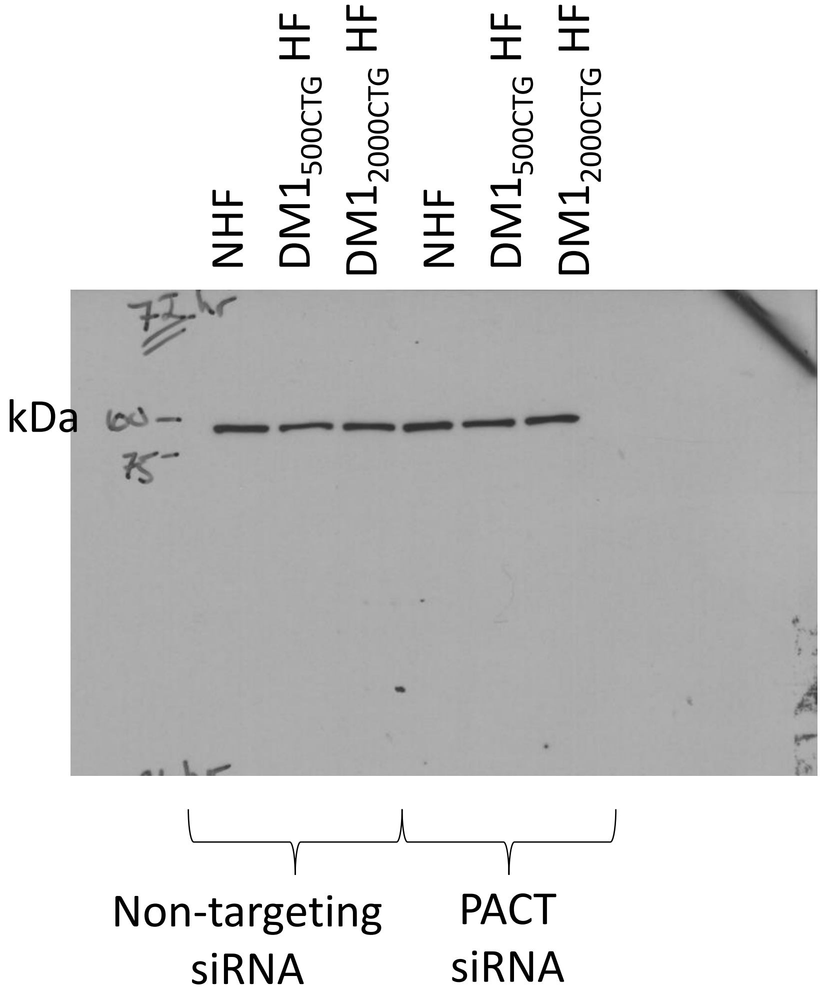

Figure 3, Supplementary Figure 6A-B
Trial 2:
anti-PACT (film, chemiluminescence)

SS = Non-targeting siRNA
PACT = PACT siRNA
*blot shows time-course of 24hr, 48hr, 72hr and 96hr siRNA treatment, but only 72hr bands were used in quantification

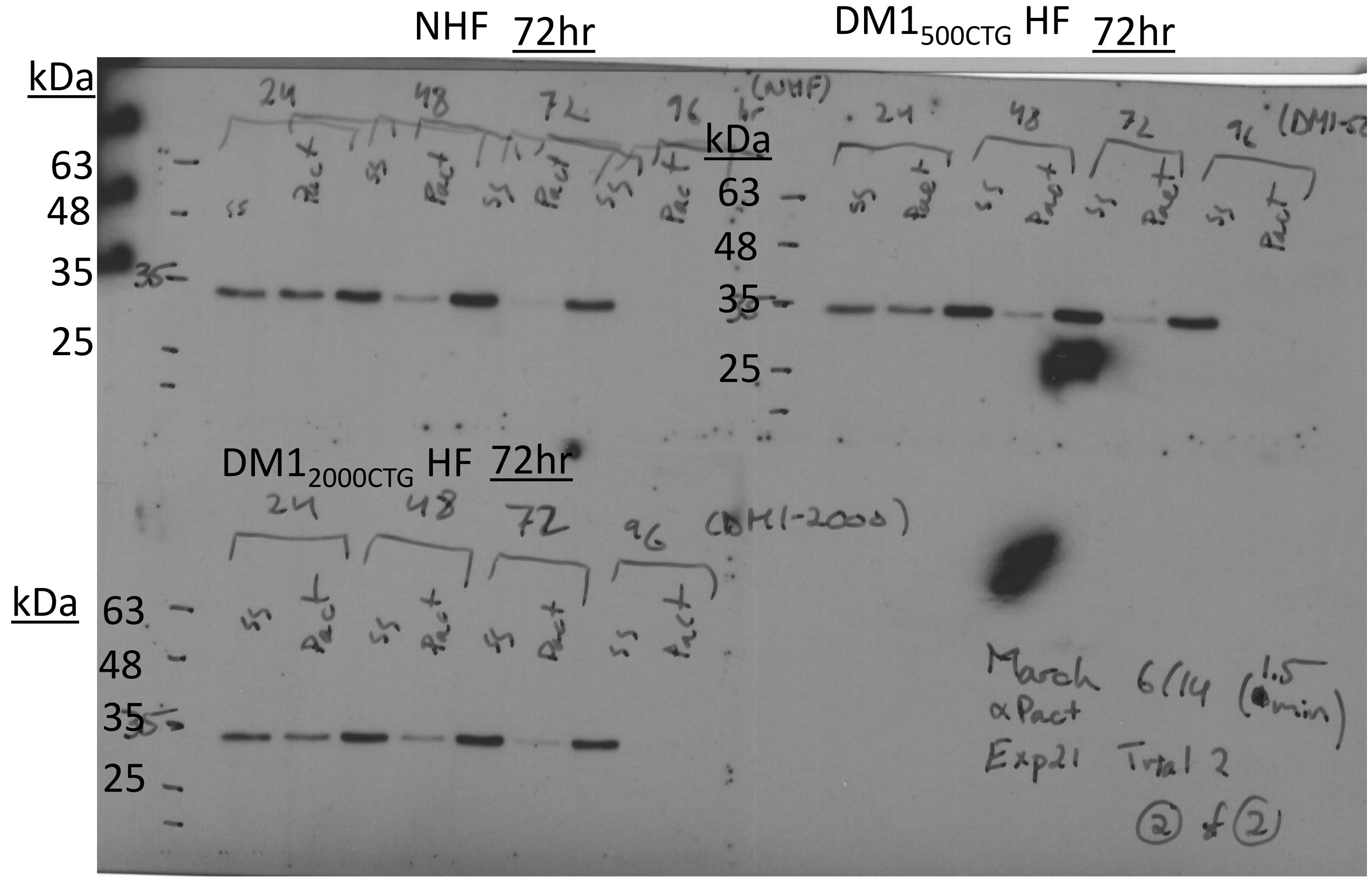

Figure 3, Supplementary Figure 6A-B
Trial 2:
anti-MBNL1 (film, chemiluminescence)

Ss = Non-targeting siRNA
PACT = PACT siRNA
*blot shows time-course of 24hr, 48hr, 72hr and 96hr siRNA treatment, but only 72hr bands were used in quantification

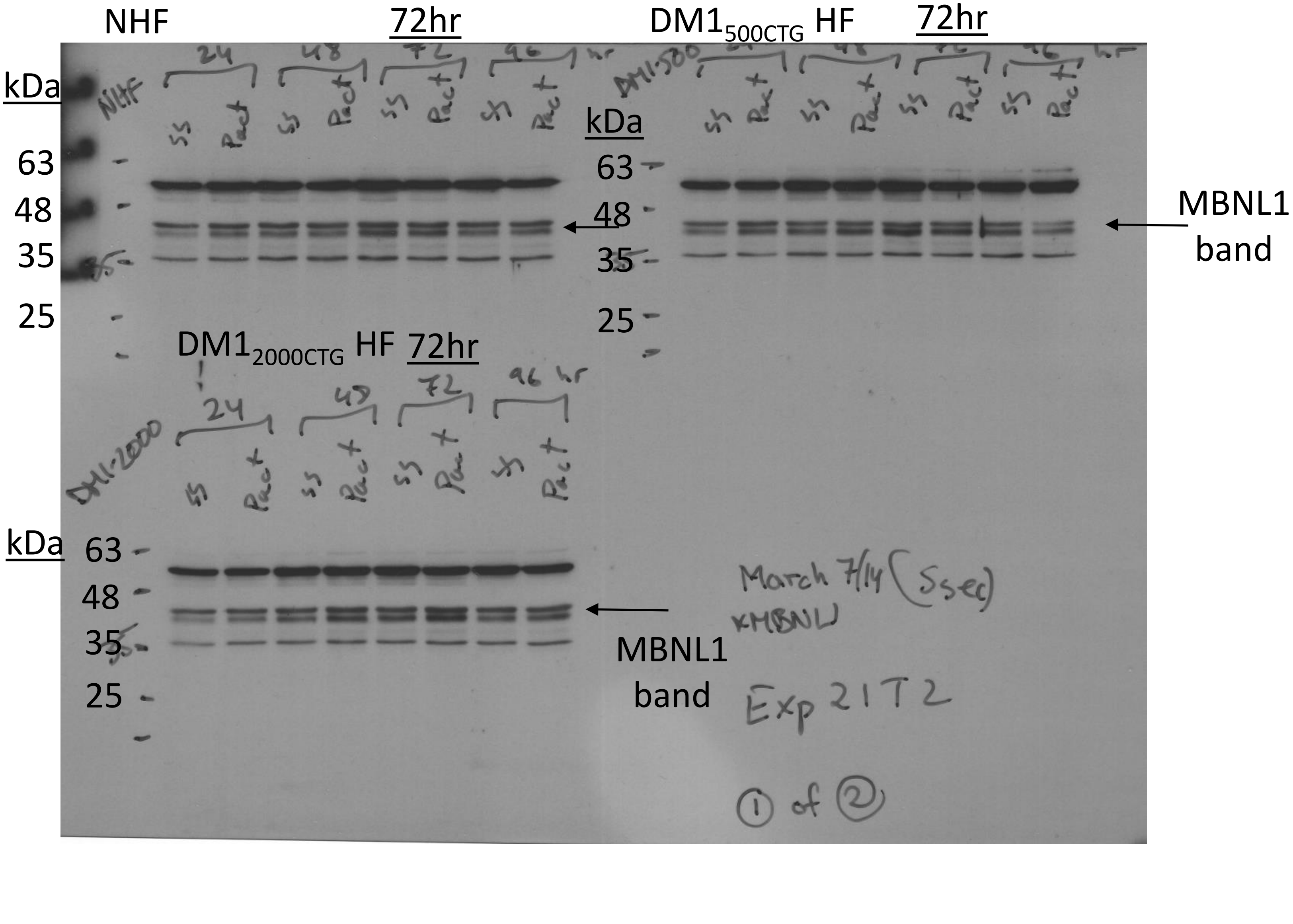

Figure 3, Supplementary Figure 6A-B
Trial 2:
anti-HSP90 (film, chemiluminescence)

Ss = Non-targeting siRNA
P = PACT siRNA
*blot shows time-course of 24hr, 48hr, 72hr and 96hr siRNA treatment, but only 72hr bands were used in quantification

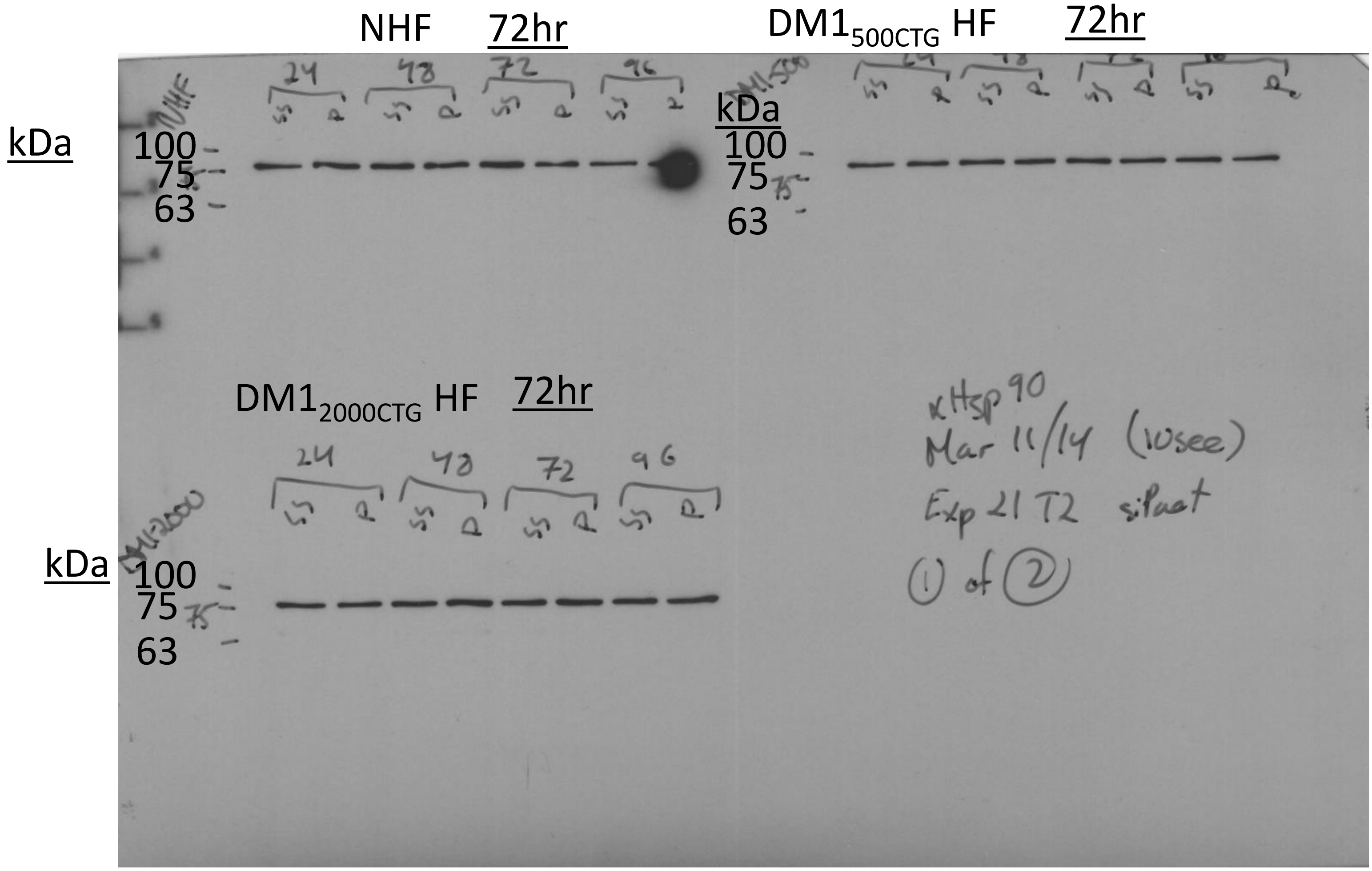

Figure 3, Supplementary Figure 6A-B
Trial 3:
anti-PACT (film, chemiluminescence)

SS = Non-targeting siRNA
PACT = PACT siRNA
*blot shows time-course of 24hr, 48hr, 72hr and 96hr siRNA treatment, but only 72hr bands were used in quantification

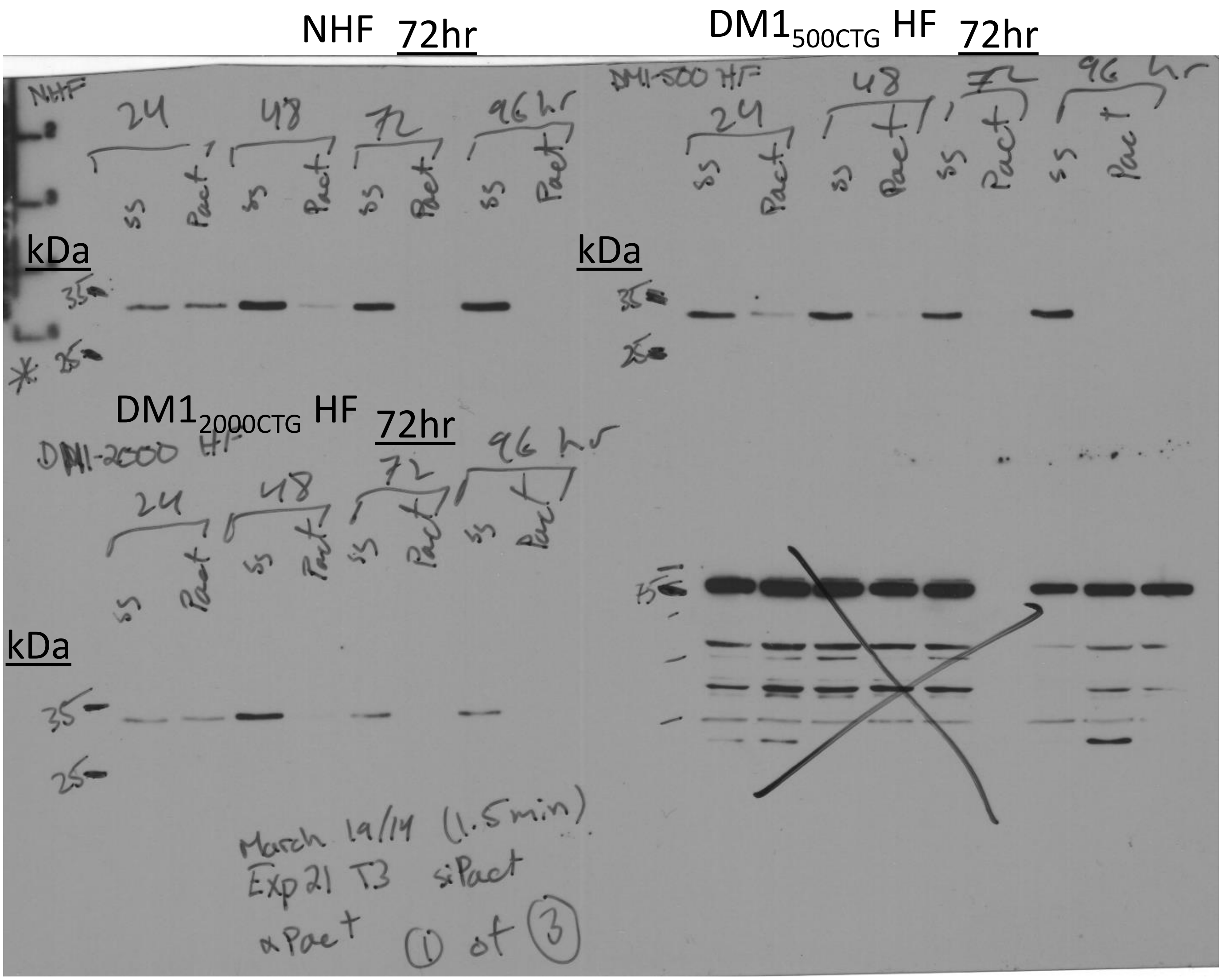

Figure 3, Supplementary Figure 6A-B
Trial 3:
anti-MBNL1 (film, chemiluminescence)

C = Non-targeting siRNA
PACT = PACT siRNA
*blot shows time-course of 24hr, 48hr, 72hr and 96hr siRNA treatment, but only 72hr bands were used in quantification
* Check mark indicates MBNL1 bands

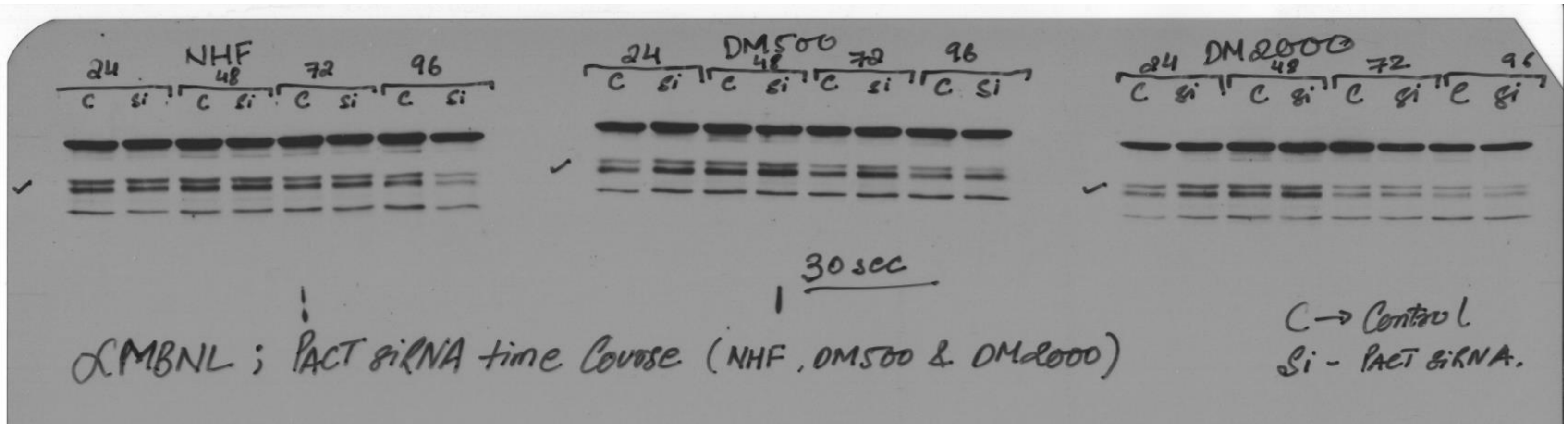

Figure 3, Supplementary Figure 6A-B
Trial 3:
anti-HSP90 (film, chemiluminescence)

ss = Non-targeting siRNA
PACT = PACT siRNA
*blot shows time-course of 24hr, 48hr, 72hr and 96hr siRNA treatment, but only 72hr bands were used in quantification

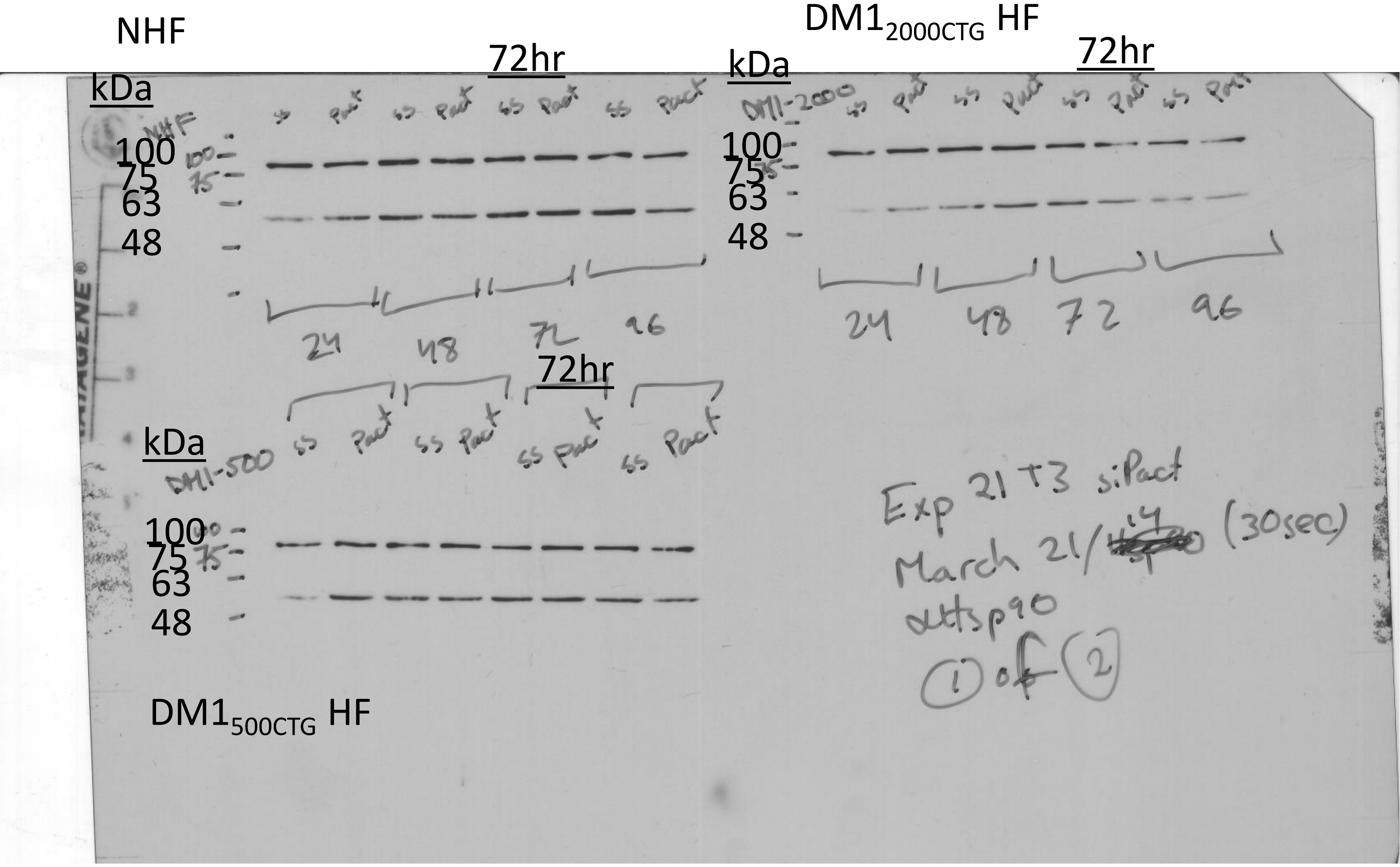

Figure 3, Supplementary Figure 3, Supplementary Figure 6A-B
Trial 4:
anti-PACT (film, chemiluminescence)

SS = Non-targeting siRNA
PACT = PACT siRNA
*blot shows time-course of 24hr, 48hr, 72hr and 96hr siRNA treatment, but only 72hr bands were used in quantification

NHF 72hr

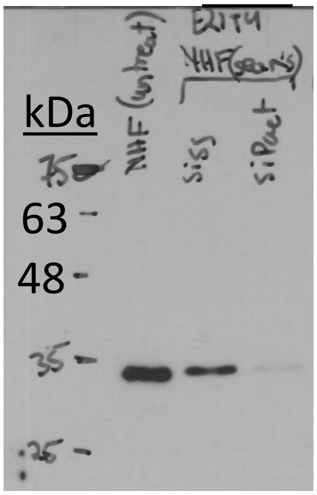

DM1$_{500CTG}$ HF 72hr DM1$_{2000CTG}$ HF 72hr

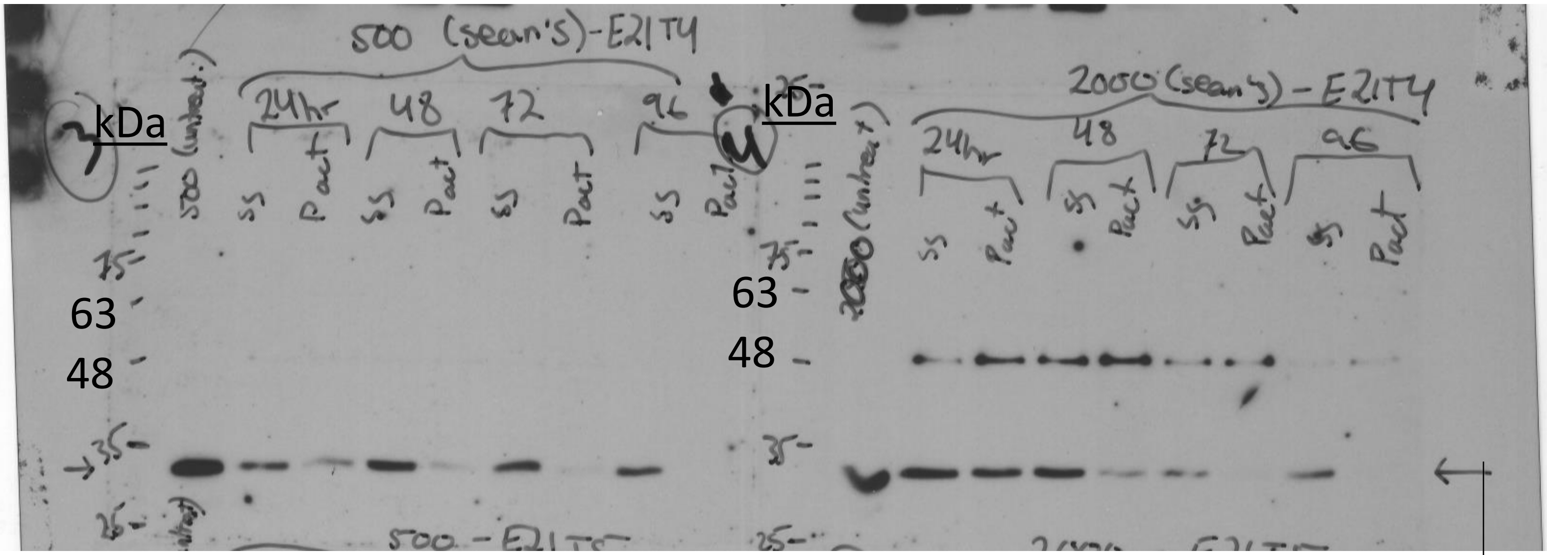

PACT band

Figure 3, Supplementary Figure 6A-B
Trial 4:
anti-MBNL1 (film, chemiluminescence)

ss = Non-targeting siRNA
PACT = PACT siRNA
*blot shows time-course of 24hr, 48hr, 72hr and 96hr siRNA treatment, but only 72hr bands were used in quantification
* Check mark indicates MBNL1 bands

NHF 72hr

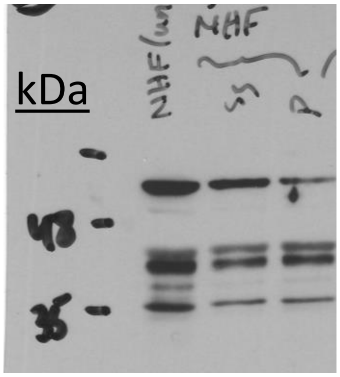

$DM1_{2000CTG}$ HF 72hr

$DM1_{500CTG}$ HF 72hr

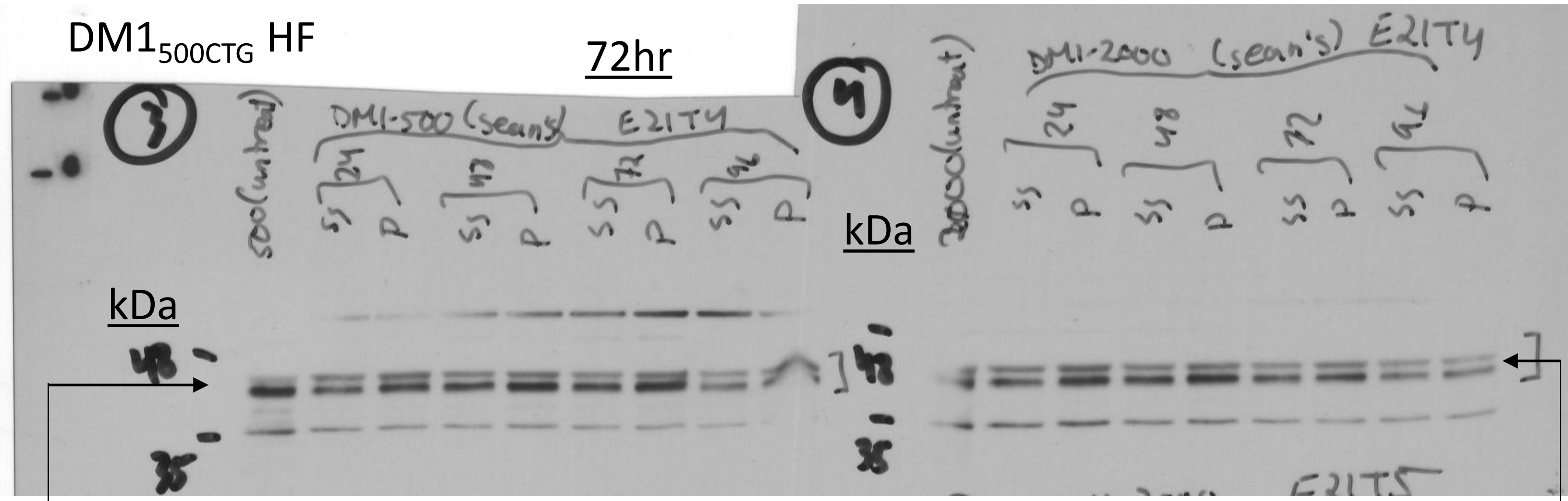

Figure 3, Supplementary Figure 6A-B
Trial 4:
anti-HSP90 (film, chemiluminescence)

ss = Non-targeting siRNA
PACT = PACT siRNA
*blot shows time-course of 24hr, 48hr, 72hr and 96hr siRNA treatment, but only 72hr bands were used in quantification

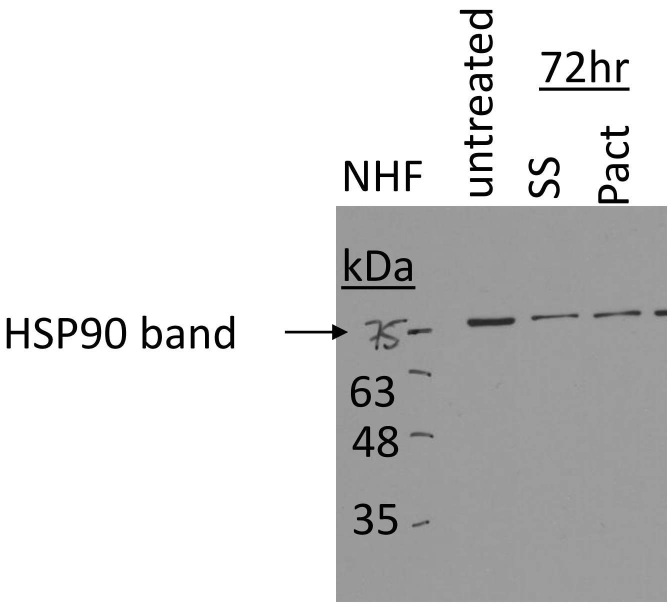

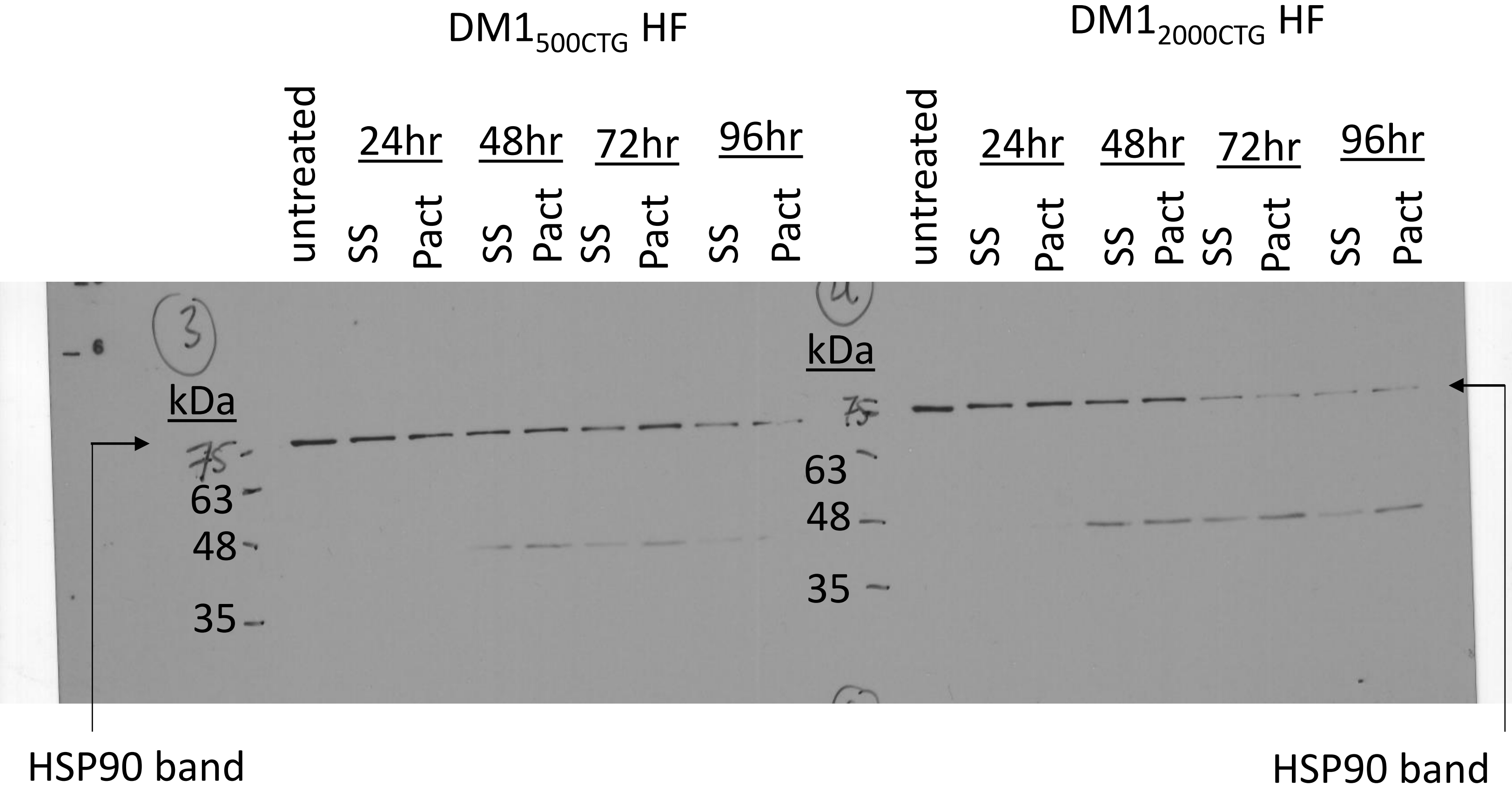

Supplementary Figure 2

anti-PACT (film, chemiluminescence) – blots used in final figure are squared
CTR = non-targeting siRNA; 24, 48, 72, 96 (hr) = time-course with PACT siRNA

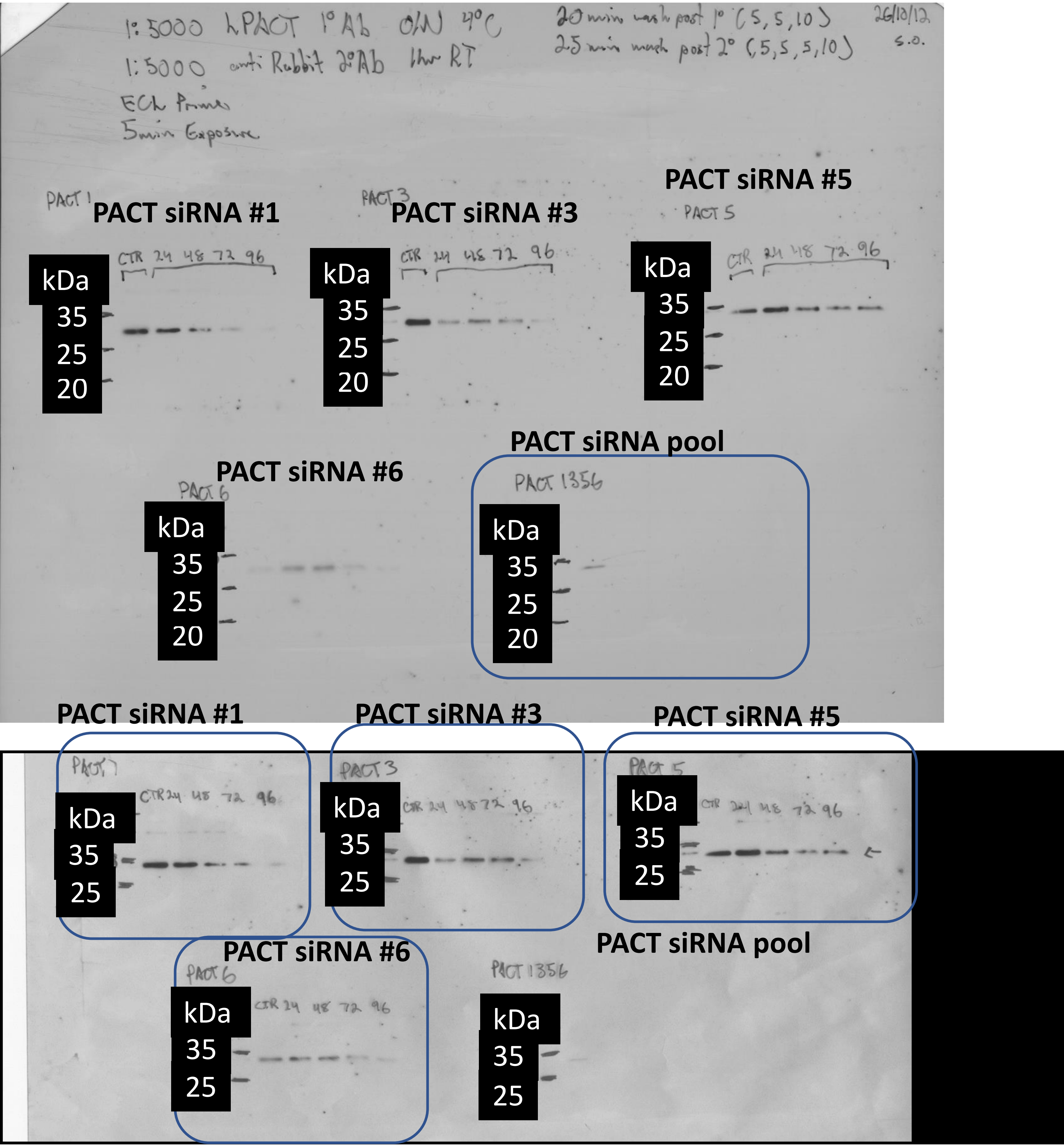

Supplementary Figure 2

anti-Tubulin (film, chemiluminescence)
CTR = non-targeting siRNA; 24, 48, 72, 96 (hr) = time-course with PACT siRNA

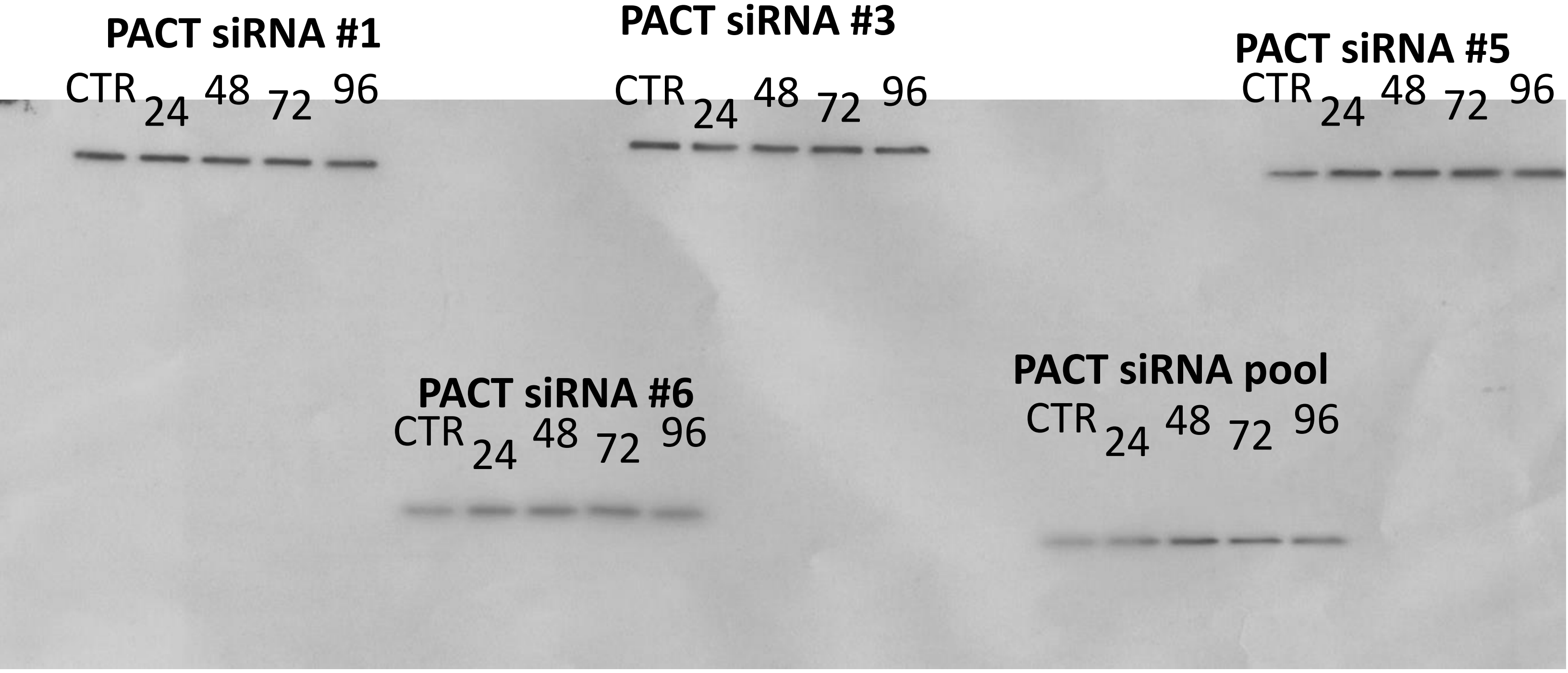

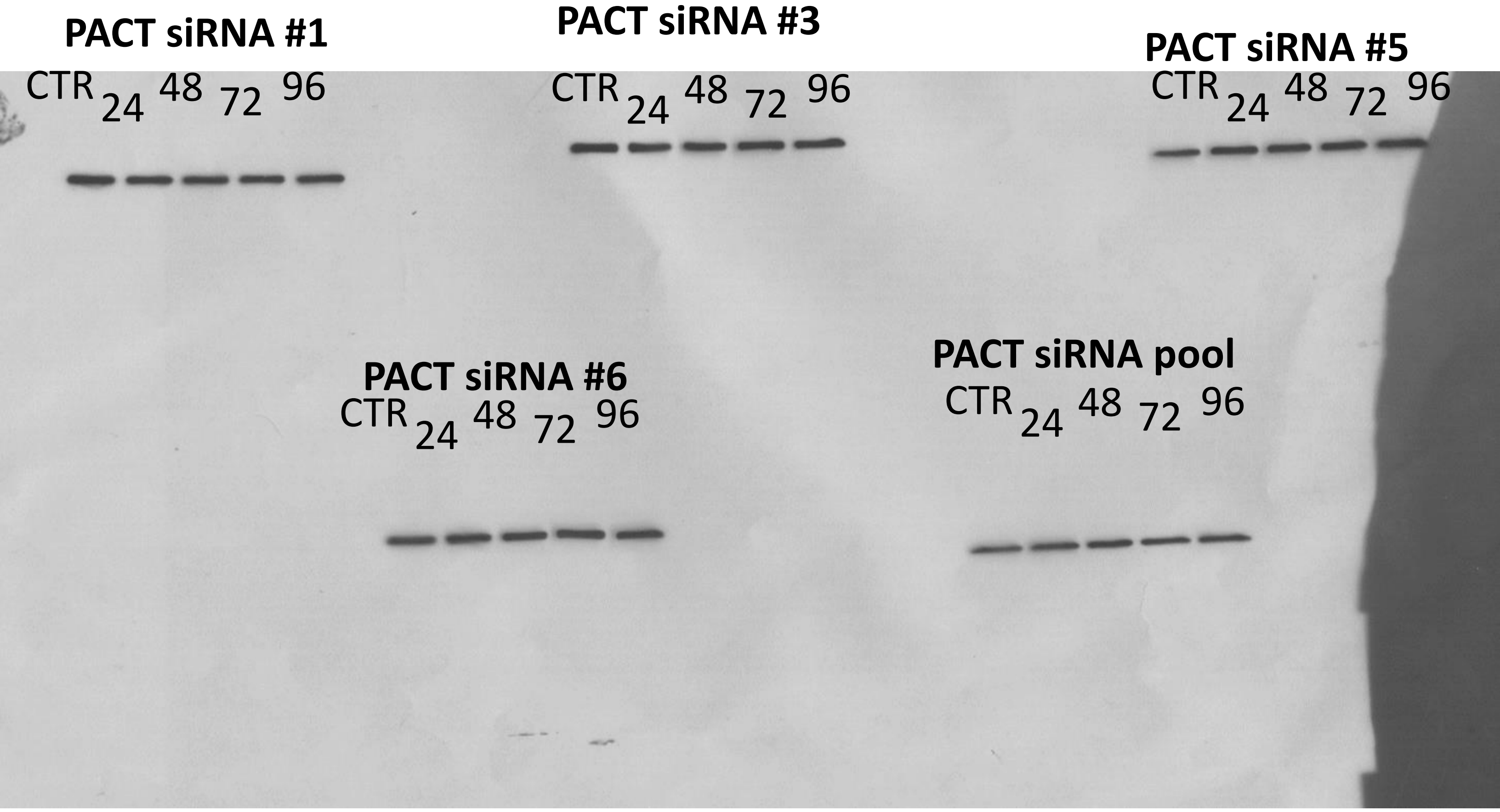

Supplementary Figure 3A-B

anti-PACT (film, chemiluminescence)

SS = Non-targeting siRNA
PACT = PACT siRNA

DM1$_{500CTG}$ HF

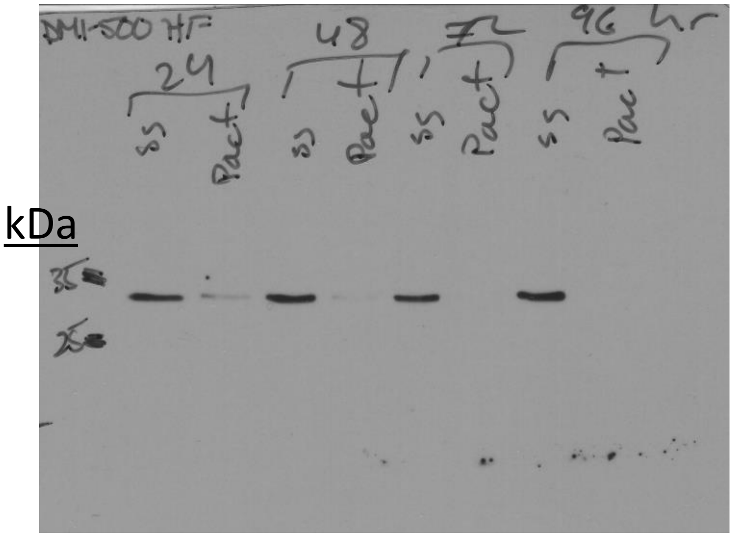

DM1$_{2000CTG}$ HF

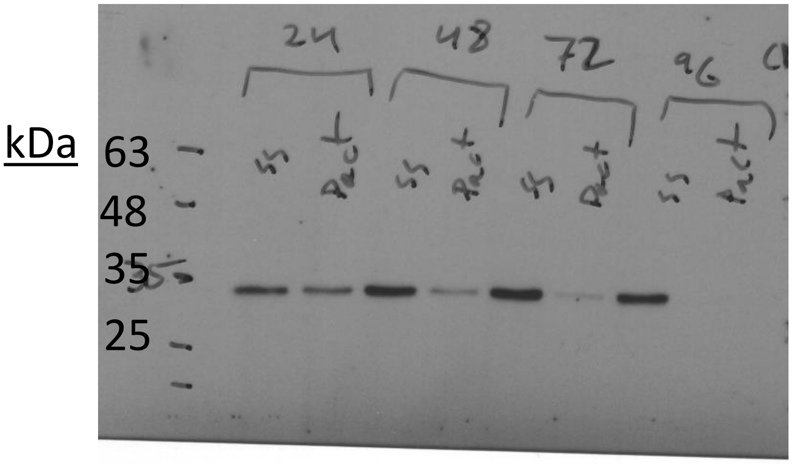

anti-HSP90 (film, chemiluminescence)

ss = Non-targeting siRNA
PACT = PACT siRNA

DM1$_{500CTG}$ HF

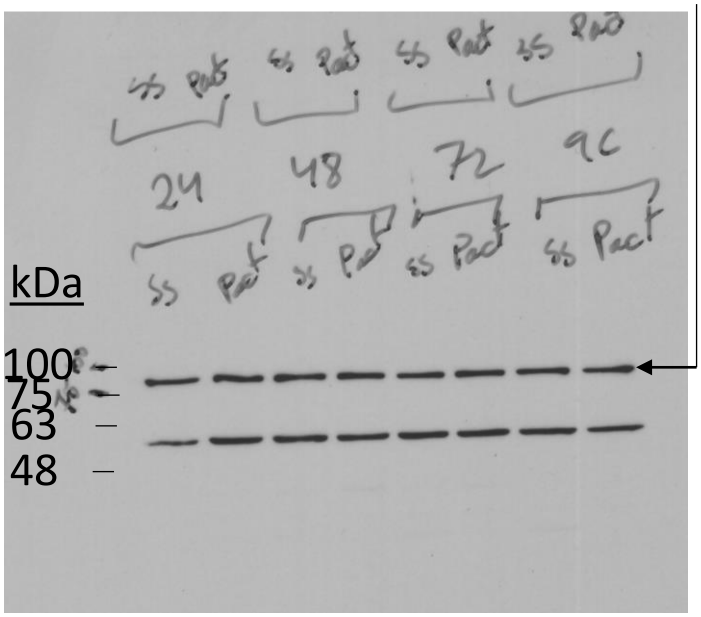

DM1$_{2000CTG}$ HF

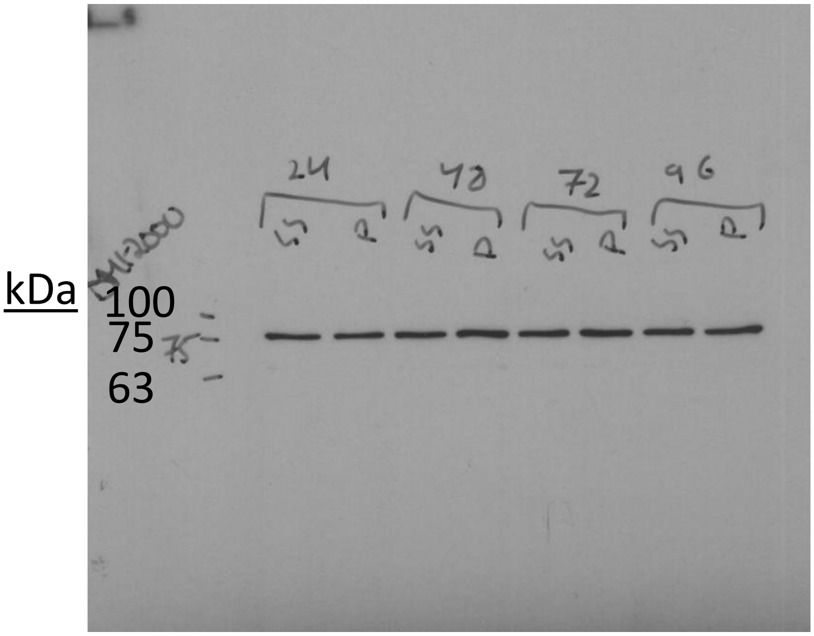

Supplementary Figure 7

anti-Pact (Chemidoc, chemiluminescence)

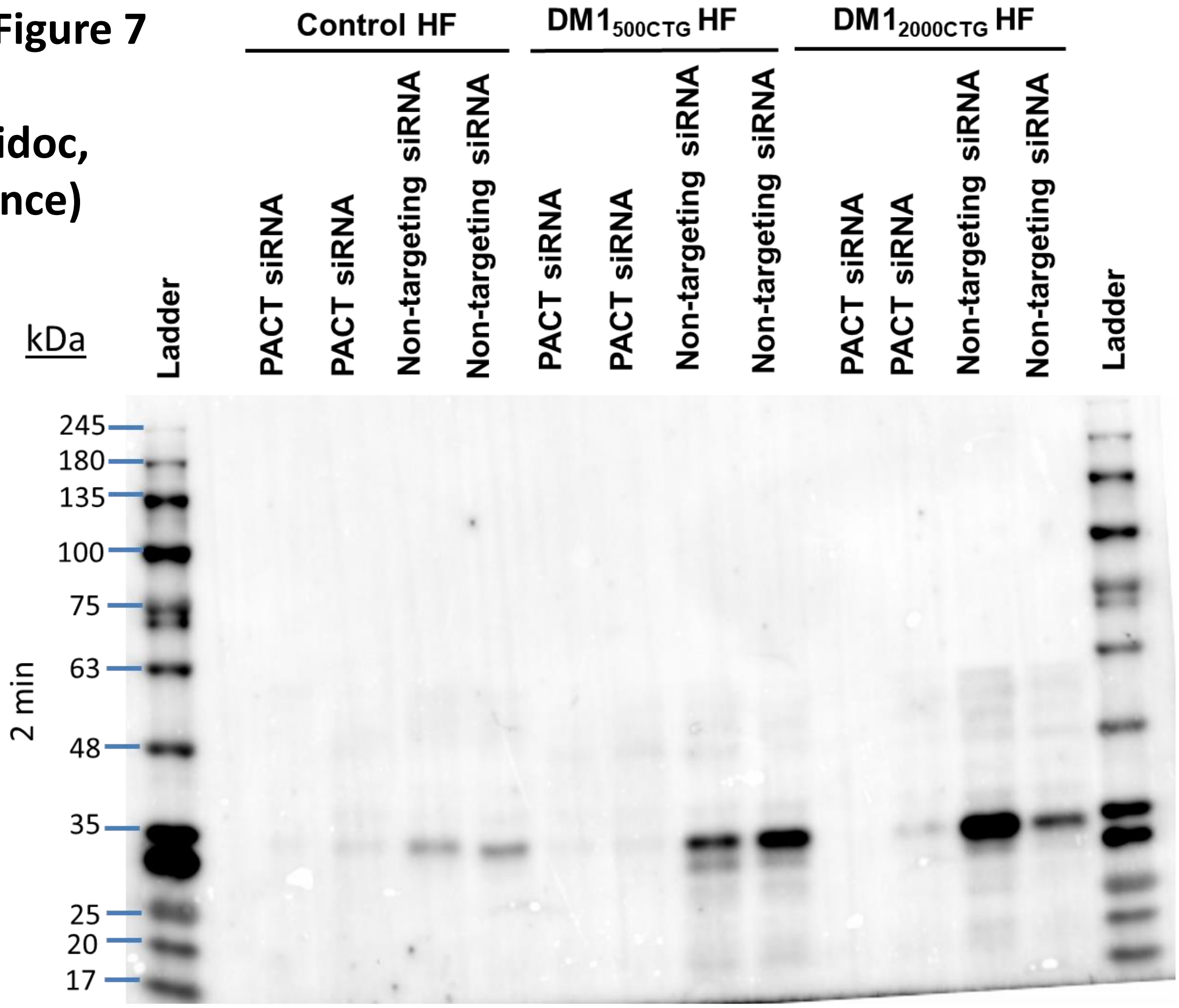

Stain-free for anti-Pact blot (Chemidoc)

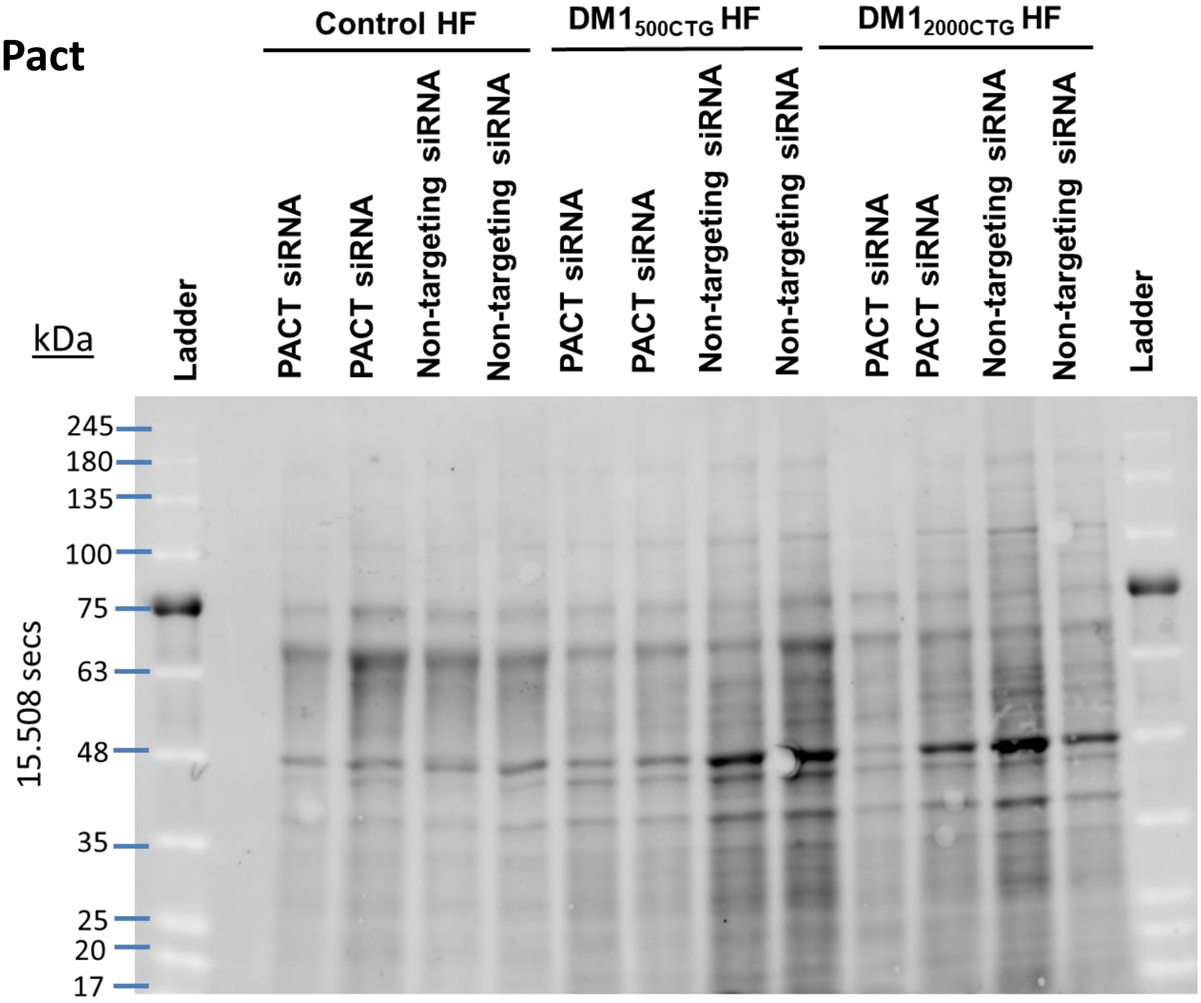

Supplementary Figure 7

anti-PKR (Chemidoc, chemiluminescence)

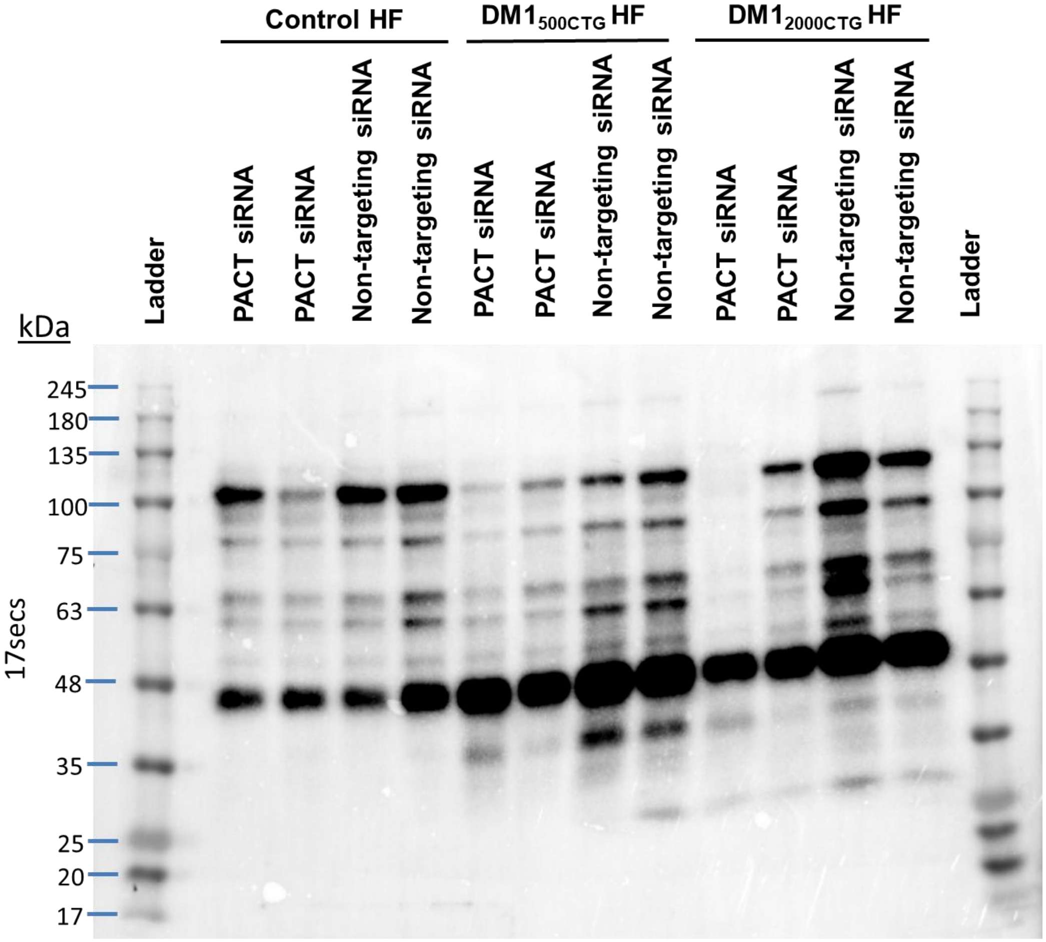

Stain-free for anti-PKR blot (Chemidoc)

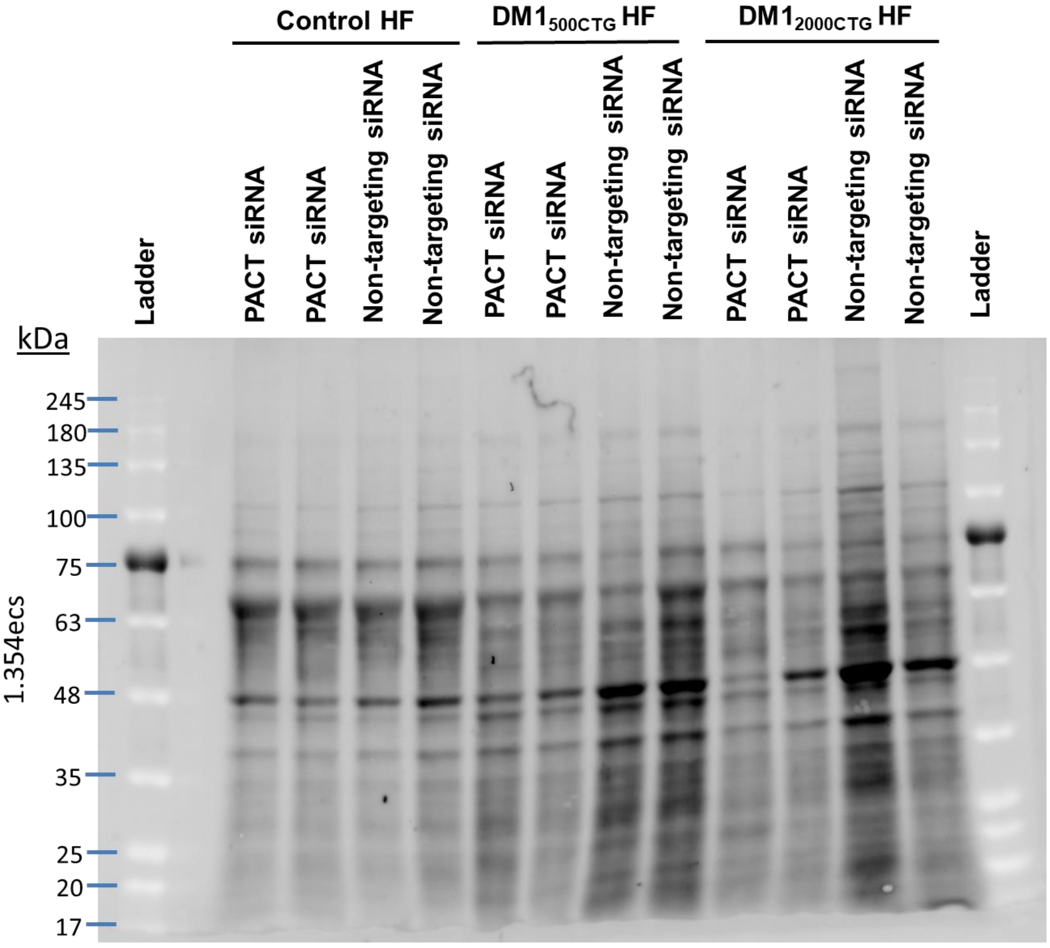

Supplementary Figure 7

anti-pPKR (T446) (chemidoc, chemiluminescence)

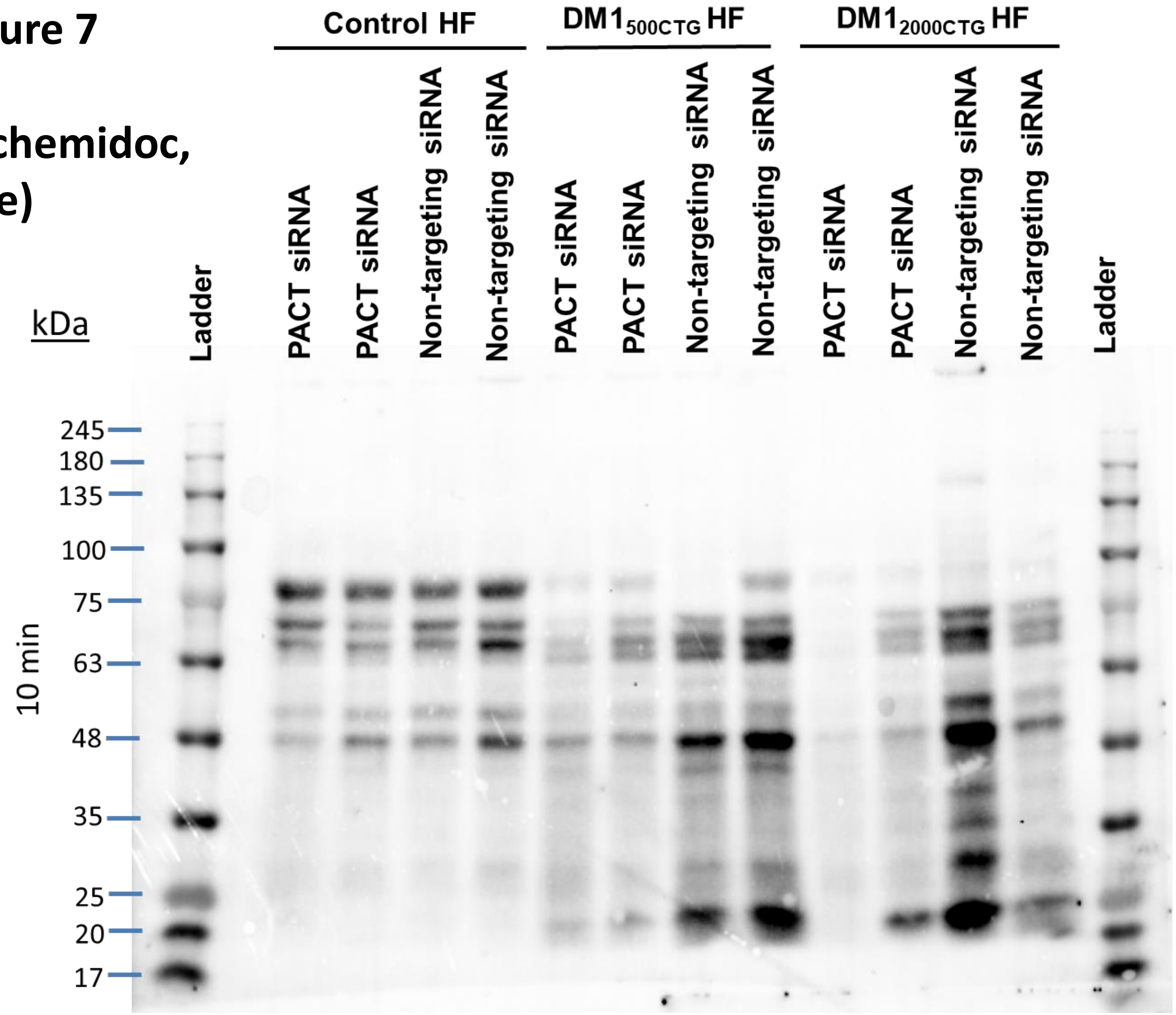

Stain-free for anti-pPKR (T446) blot (Chemidoc)

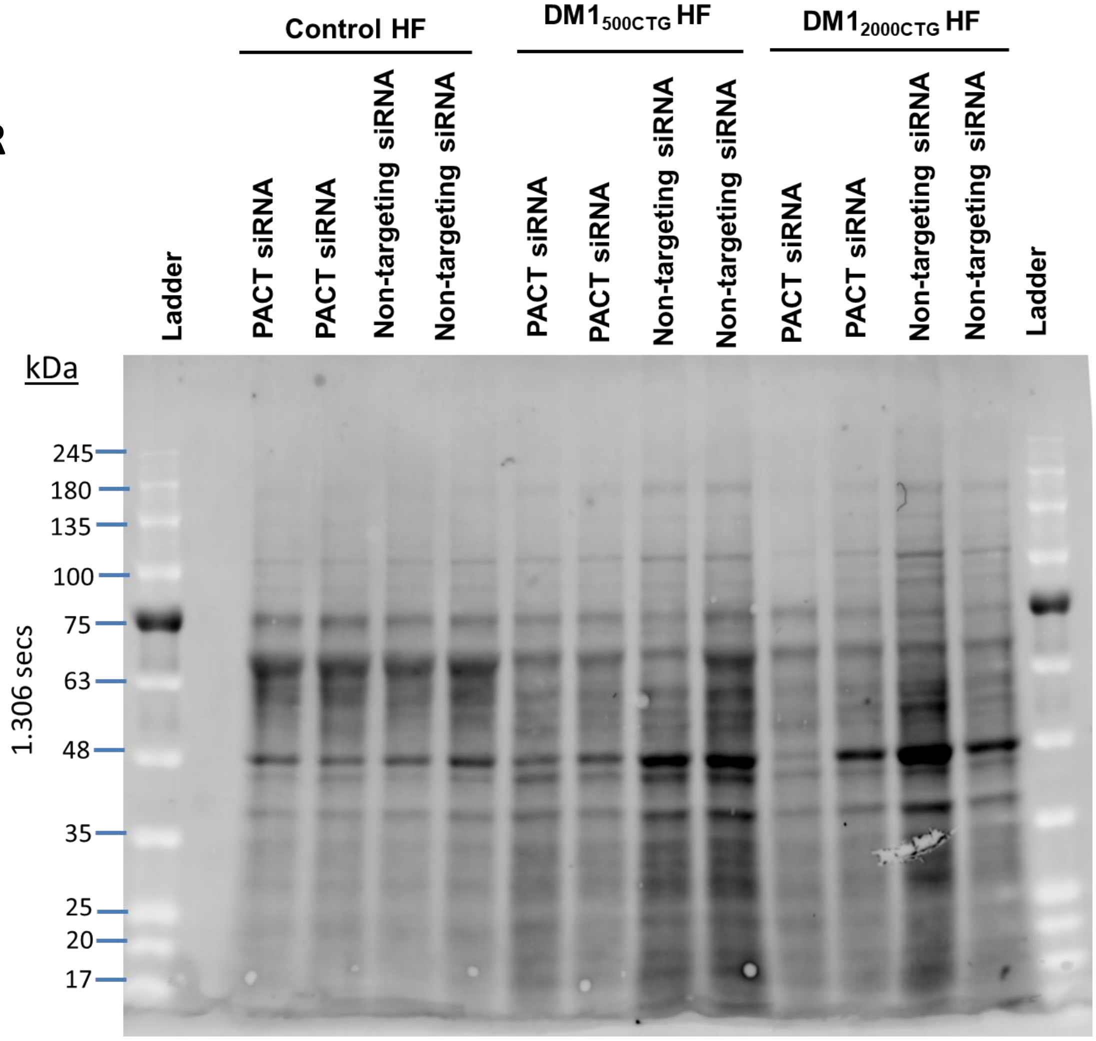

Supplement: S1 File — (PDF) [file pone.0256276.s014.pdf]
